# Usage of FT-ICR-MS Metabolomics for Characterizing the Chemical Signatures of Barrel-Aged Whisky

**DOI:** 10.3389/fchem.2018.00029

**Published:** 2018-02-22

**Authors:** Chloé Roullier-Gall, Julie Signoret, Daniel Hemmler, Michael A. Witting, Basem Kanawati, Bernhard Schäfer, Régis D. Gougeon, Philippe Schmitt-Kopplin

**Affiliations:** ^1^Comprehensive Foodomics Platform, Technische Universität München, Freising, Germany; ^2^Research Unit Analytical BioGeoChemistry, Department of Environmental Sciences, Helmholtz Zentrum München, Neuherberg, Germany; ^3^Independent Spirit Consultant, Nuremberg, Germany; ^4^UMR PAM Université de Bourgogne, AgroSupDijon, Institut Universitaire de la Vigne et du Vin, Dijon, France

**Keywords:** whisky, FT-ICR-MS, barrel, wood, rum, distillate, LC-MS

## Abstract

Whisky can be described as a complex matrix integrating the chemical history from the fermented cereals, the wooden barrels, the specific distillery processes, aging, and environmental factors. In this study, using Fourier transform ion cyclotron resonance mass spectrometry (FT-ICR-MS) and liquid chromatography coupled with tandem mass spectrometry (LC-MS/MS), we analyzed 150 whisky samples from 49 different distilleries, 7 countries, and ranging from 1 day new make spirit to 43 years of maturation with different types of barrel. Chemometrics revealed the unexpected impact of the wood history on the distillate's composition during barrel aging, regardless of the whisky origin. Flavonols, oligolignols, and fatty acids are examples of important chemical signatures for Bourbon casks, whereas a high number of polyphenol glycosides, including for instance quercetin-glucuronide or myricetin-glucoside as potential candidates, and carbohydrates would discriminate Sherry casks. However, the comparison of barrel aged rums and whiskies revealed specific signatures, highlighting the importance of the initial composition of the distillate and the distillery processes.

## Introduction

Among distilled beverages, whisky is definitely associated with a high value and long-lasting traditional product from Scotland and Ireland, although it is nowadays made in various countries around the world. Whiskies are produced by fermenting malted barley and other cereals including wheat and maize (Bringhurst and Brosnan, 2014). The fermented product is then distilled and matured in oak barrels (Liebmann and Scherl, 1949; Piggott et al., 1989; Mosedale and Puech, 1998). Only water and spirit caramel (E150) may then be added prior to bottling the whisky at an alcoholic strength of at least 40% *v/v* in the European Union [(The Scotch Whisky regulations, 2009) defines the characteristic for the Scotch Whiskies in dependence on the regulation (EC) No 110/2008 of the European parliament Annex 2 No. 2]. Scotch whiskies are matured in barrels of oak wood for a minimum of 3 years before consumption, but many whiskies are matured for 12 or more years. Traditionally, Scotch whiskies are aged in Spanish oak barrels (butt) which had previously contained Sherry wine (Conner et al., 1993; Piggott et al., 1993). In contrast, American Bourbon whiskies are matured in new oak barrels (the 180 liters barrel). However, it is a common practice for Scotch whisky distillers also to use barrels, which had previously contained Bourbon whisky or even wines other than Sherry (Piggott et al., 1993).

The composition of a matured whisky is thus the result of complex interplays between the various contributions from each step involved in the production process including cereals malting, distillation, and barrel aging (Piggott et al., 1989; Reid et al., 1993). However, the maturation in barrels is considered crucial for fine-tuning the sensory properties of the new-make spirit (distillate). Many aspects of the barrel aging process have been evaluated, like the effect of ethanol concentration on the extraction of aroma and flavor compounds from wood (Boruff and Rittschof, 1959; Lee et al., 2001; MacNamara et al., 2001, 2011) or the effect of temperature and humidity during aging (Liebmann and Scherl, 1949; Swan and Burtles, 1978). The impact of the barrel making processes including toasting and charring of the interior surfaces of the barrel (Conner et al., 1993; Reid et al., 1993; Mosedale and Puech, 1998; Labbé et al., 2006) and the impact of the aging duration have been evaluated as well (Liebmann and Scherl, 1949; Nose et al., 2004; MacNamara et al., 2011). During aging in barrels, various reactions between the wood and the distillate occur, which include in particular the extraction of wood volatiles (lactones, lignin, and hemicelluloses degradation products) and ellagitannins. Oak barrel components are therefore primary contributors to the composition of aged whisky (Puech, 1987; Piggott et al., 1993). Piggott et al. described differences in the chemical composition depending on barrel history (new or used barrel, cask size, barrel previously filled with bourbon or wine; Piggott et al., 1993). Used barrels release lower concentrations in most extractives and generally lead to a longer maturation time than new casks (Piggott et al., 1993; Singleton, 1995; Mosedale and Puech, 1998; Cerdán et al., 2002). This has particular consequences for the Scotch whisky industry, which rarely purchases new casks but depends on the re-use of casks after maturation of other alcoholic beverages.

The process of maturation and aging is characterized by a decline in both the volume and the alcoholic content and changes in color and flavor of the maturing spirit (Masuda and Nishimura, 1982; MacNamara et al., 2001; Alcarde et al., 2014; Holden, 2016). Several processes may be involved in these changes. Direct extraction of wood compounds and decomposition of wood macromolecules followed by the extraction of their products into the distillate may have some influence on maturation and aging. Reactions between wood components and aromas of the raw distillate, reactions involving only the distillate components and, finally, the evaporation of volatile compounds could also cause these changes (Nishimura et al., 1989).

Understanding the chemical composition of whisky and the impact of each step in the manufacturing process provides a basis for responding to the challenges of protecting the integrity from fraudulent activities, and for supporting the need to produce high quality spirits (Collins et al., 2014; Wiśniewska et al., 2015). To that respect, gas and liquid chromatography have been shown to be efficient methodologies for the analysis of whiskies and have contributed to a better knowledge of their composition (Piggott et al., 1993; Collins et al., 2014; Wiśniewska et al., 2015; Kew et al., 2016). Metabolomics approaches have recently shown great potential for exploring the complex chemistry of biological samples such as food, beer, wine, champagne, and whisky (Cevallos-Cevallos et al., 2009; Cajka et al., 2011; Garcia et al., 2013; Roullier-Gall et al., 2014; Jeandet et al., 2015; Kew et al., 2016). However, only few studies on whisky which compared the chemical signatures of Scotch and American whisky and considered the identification of counterfeit products have been reported so far (Møller et al., 2005). The study recently published by Kew et al. (2016), reporting the 12 Tesla FT-ICR-MS analysis of a series of whiskies, provides an unprecedented representation of the chemical diversity and complexity of these spirits. Multivariate statistical analysis of a series of Scotch whiskies showed the possibility to classify and discriminate whiskies according to their blending process and casks type used in maturation.

In this study, we further explored this chemical diversity by non-targeted FT-ICR-MS and LC-MS/MS based metabolomics, but considering up to 150 whisky samples from 49 distinct distilleries originating from seven different countries. This study not only highlights the impact of the wood and in particular the history of the barrel on the distillate composition during aging, but reveals that some particular features (impact of wood history) can be discriminated regardless of the origin, the composition and the aging time. In order to characterize specific wood and distillates contributions, barrel wood extracts, and barrel-aged rums were also analyzed.

## Materials and methods

### Whisky samples

One hundred and fifty whisky samples from 49 different distilleries, 7 countries (Scotland, USA but also France, Germany, Japan, Canada, and Austria), from 1 day new make spirit to 43 years of maturation, were analyzed. The whiskies were aged in different types of oak barrels (Sherry Casks, Bourbon barrel, or new make casks) and for different durations (from 1 day new make spirit to 43 years). Different bottling processes were also represented: single cask or blended (Supplemental Table 1). All samples were collected directly from the bottle and stored in 10 mL amber vials at room temperature prior to the preparation for analysis.

### Rum samples

Four Rum samples from France, Jamaica, Puerto Rico, and Barbados were analyzed (Supplemental Table 2). All samples were collected directly from the bottle and stored in 10 mL amber vials at room temperature prior to the preparation for analysis as described below.

### Wood samples

Twenty barrel extracts made of wood from nine different French forests and two oak wood species (sessile and pedunculated) were selected. All samples were prepared by hydro-alcoholic extraction, solubilizing 20 mg of the wood in 1 mL of a methanol/water (8:2 *v/v*) solution for 1 min in an ultrasonic bath at 20°C. After centrifugation (5 min, 25,400 g), 50 μL of the supernatant was diluted in 1 ml of methanol before injection. For additional details on the barrel elaboration and the oak wood extract used, see Gougeon et al. (2009b).

### Standards

Gallic acid and ellagic acid reference standards were purchased from Sigma-Aldrich, St. Louis, USA.

### FT-ICR-MS metabolome profiling

Ultrahigh-resolution mass spectra were acquired on a Bruker solariX Fourier transform ion cyclotron resonance mass spectrometer (FT-ICR-MS) (Bruker Daltonics GmbH, Bremen, Germany) equipped with a 12 Tesla superconducting magnet (Magnex Scientific Inc., Yarnton, GB) and an Apollo II ESI source (Bruker Daltonics GmbH, Bremen, Germany) operated in the negative ionization mode. Negative ionization has already been proven to be the preferred ionization mode in fingerprinting wines by FT-ICR-MS (Roullier-Gall et al., 2014, 2015). Fifty microliters of the samples were diluted in 1 mL of methanol prior to injection. Samples were introduced into the electrospray source at a flow rate of 120 μL h^−1^. FT-ICR-MS was externally calibrated on clusters of arginine (10 mg L^−1^ in methanol). Further internal calibration was performed for each sample by using a reference list containing ubiquitous fatty acids, yielding mass accuracies lower than 0.1 ppm in routine day-to-day measurements (Gougeon et al., 2009b; Roullier-Gall et al., 2014). Spectra were acquired with a time domain of 4 mega words over a mass range from *m/z* 100 to 1,000. Four hundred scans were accumulated for each sample corresponding to a run time of 18 min. In total, 50 μL diluted samples (2.5 μL original sample) were consumed for one mass spectrum, which makes the approach not only compatible for high sample throughput but also requires small sample amounts (Witting et al., 2014). Quality control (QC) samples were prepared by pooling equal amounts of all samples. QC samples were analyzed at the beginning and after every 10 samples to monitor the reproducibility of the measurements (Supplemental Figure 1).

### Statistical analysis

FT-ICR-MS raw data were first aligned in order to discover occurring patterns, to identify outliers and to reduce the dimensionality of the data (Lucio, 2009). Peak alignment (alignment window: 1 ppm) and filtering of ion masses detected in at least 10% of all samples were performed using an in-house developed software tool (Roullier-Gall et al., 2014). Partial least squares discriminated analysis (PLS-DA) was performed with Simca-P 9.0 (Umetrics, Sweden) and Hierarchical Cluster Analysis (HCA) with Perseus 1.5.1.6 (Max Planck Institute of Biochemistry, Germany).The repeatability overtime analysis of FT-ICR-MS for the whisky analysis during the all running time was measured using coefficient of variation of 10 QC whisky intensities and comparison of mass spectra diversity (Supplemental Figure 1). A QC sample was analyzed every 10 samples to confirm the stability of acquisition over running time. The 10 QC spectra obtained after the analysis were very consistent throughout confirming the repeatability over time of the FT-ICR-MS analysis (Supplemental Figure 1). The good similarity between QC spectra could also be visualized using principal component analysis (PCA—Supplemental Figure 1). All QC samples (in red) are grouped together according to the first and the second axis (explaining 18.3% of the total variability), and the whisky samples (in gray) are distributed around the all first two dimension of the PCA. A coefficient of variation over the run time was calculated based on the 10 injection of the QC sample throughout the sequence. Coefficients of variation of the QC revealed excellent homogenization profiles (Supplemental Figure 1) with coefficient of variation lower than 0.2 for 92.8% of elemental formulas. The count of formulas frequencies among the QC samples shown 2,409 formulas (out of 3,015 in total) which were detected in at least 50% of the 10 QC samples including 1,565 formulas detected in the entire QC samples (Supplemental Figure 1). These results demonstrate the excellent repeatability of FT-ICR-MS analysis over time.

### UHPLC-QToF-MS/MS experiments

Marker peaks from statistical analyses were subjected to tandem MS. Whisky samples were directly injected in the LC-MS/MS without any dilution. The preparation of standards consists of the dilution at 50 ppm using a solution water/acetonitrile (80/20 V/V). Target lists for fragmentation experiments were converted to MS/MS acquisition methods using MetShot (Neumann et al., 2013). Metabolites were separated using a Waters Acquity UPLC system coupled to a Bruker maXis UHR-ToF-MS. A reversed-phase (RP) separation method was employed which separates middle to non-polar metabolites using a BEH C18 (100 × 2.1 mm ID, 1.7 μm) from Waters. Under the optimized condition, the column oven was thermostated at 40°C. Eluent A consisted of 10% acetonitrile (ACN) in water and Eluent B of 100% ACN, both with 0.1% formic acid. Detection was carried out in negative ionization mode with the following parameters: Nebulizer pressure = 2.0 bar, dry gas flow = 8.0 l/min, dry gas temperature = 200°C, capillary voltage = 3,500 V, end plate offset = −500 V. The flow rate was 0.4 mL/min. UHR-ToF-MS acquisitions were carried out in profile spectra mode with 1 Hz accumulation time. Instrument tuning focused on detection and resolution of molecular weight compounds in the mass range of 50–2,000 Da. Mass calibration was carried with low concentration ESI Tuning Mix (Agilent, Waldbronn, Germany). After acquisition, MS/MS spectra were manually extracted using Bruker Data Analysis 4.1 (Bruker Daltonic, Bremen, Germany).

## Results and discussion

### CHONS chemical space in whiskies

**Figure 1:**
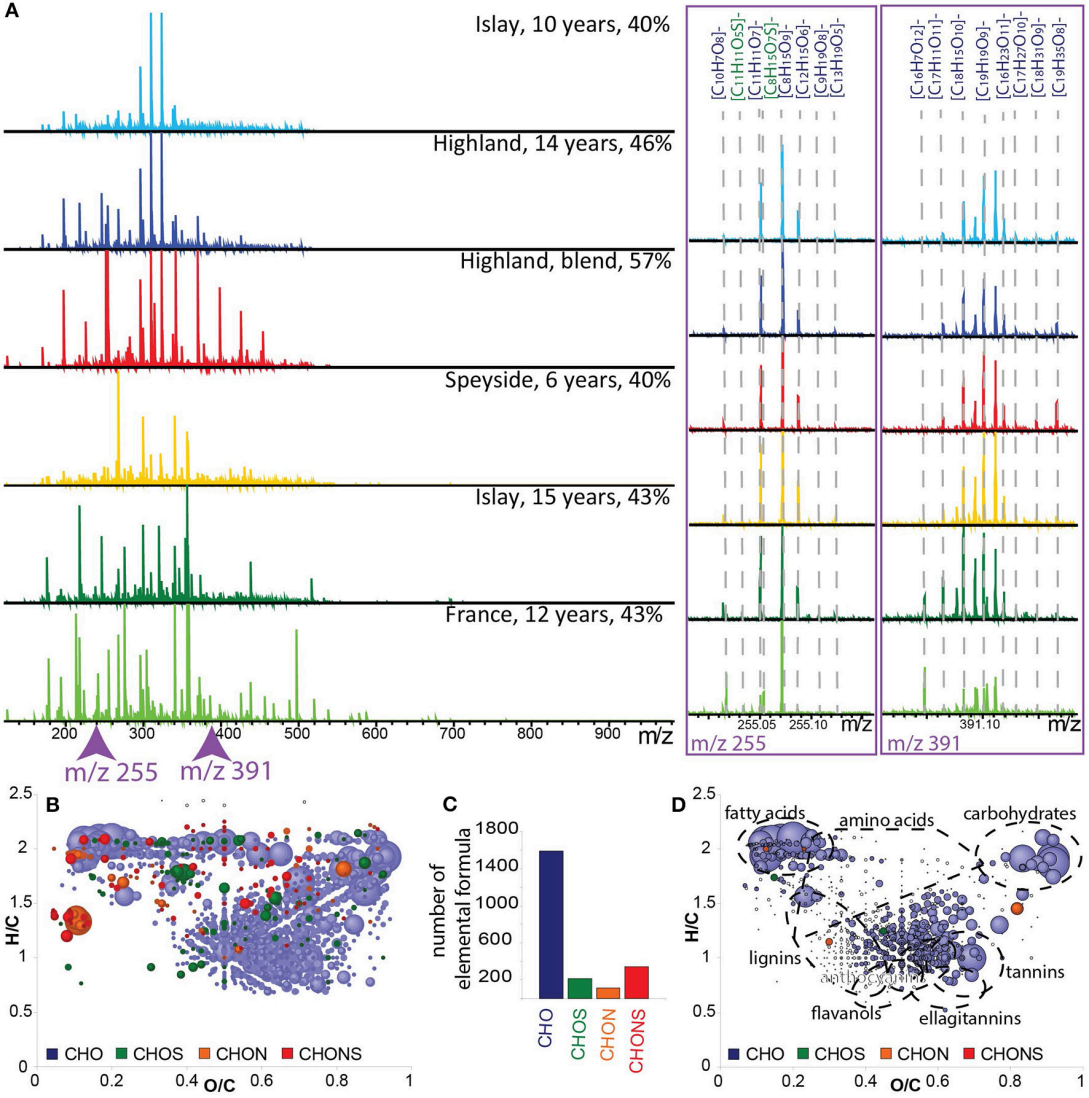
**(A)** Visualization of ESI(-) FT-ICR-MS spectra of six Whisky samples from various geographical origins, aging time in barrel and alcoholic strengths (% v/v), in the mass range from 100–1,000 Da together with enlargements of the nominal masses *m/z* 255 and *m/z* 391 showing the molecular formula assignments. **(B)** Van Krevelen diagram (H/C vs. O/C) of the global composition of 150 whisky samples. **(C)** Frequency histogram of CHO, CHOS, CHON, and CHONS elemental compositions displayed in **(B)**. **(D)** Van Krevelen diagram showing the 791 annotated metabolites from our home-built database. Points in van Krevelen diagrams are colored according to their elemental composition, CHO in blue, CHOS in green, CHON in orange, and CHONS in red, and scaled according to their peak intensities.

FT-ICR-MS spectra of six samples from various origins, ages, and alcoholic strengths (% *v/v*) exemplify the abundance of detected signals in a mass range from *m/z* 100 to 1,000. Several thousands of non-volatile signals were found between *m/z* 150 and 600 (Figure 1). Each spectrum (shown in Figure 1) can be considered as a representative fingerprint of that particular whisky. Full spectra and enlargements at 255 and 390 Da allow a direct visual comparison of the whisky samples. The observed differences intensities, which can be understood as a relative measure of the concentration, may originate from their distinct geographical origins or elaboration recipes, including the origin of the barrel, aging time, blending, or the final alcohol strength (Figure 1). For example the mass at *m/z* 255.05103 corresponding to the elemental formula [C_11_H_11_O_7_]^−^ is less intense in the whisky sample from France compared to all other whisky samples. However, Figure 1 also shows that there is a consistency in the chemical diversity regardless of the origin (Kew et al., 2016).

The initial screening using FT-ICR-MS resulted in a set of 5,969 *m/z* values (S/N ≥ 6) found across all samples (Table 1). Three thousand and fifteen out of the initial set of 5,969 features could be assigned to unambiguous molecular formulas (Table 1) containing the elements C, H, O, N, and S (0.2 ppm annotation tolerance). The corresponding van Krevelen diagram provides a qualitative visual description of the nature of non-volatile compounds in whiskies representative for all origins, ages, and alcohol strengths. A considerably high abundance of CHO formulas was observed (more than 1,500 formulas out of 3,015, Figures 1B,C) which is in agreement with the literature (Kew et al., 2016). Annotations with a home-built plant metabolite database gave 791 hits (Table 1). Mostly fatty acids, carbohydrates, amino acids, flavonols, ellagitannins, and lignin-derived metabolites were proposed as candidates by the database (Figure 1D).It is worth noticing that 64% of all chemical formulas did not result in a hit in the available databases (Roullier-Gall et al., 2016).

**Table 1:** Number of signals, assigned molecular formulae and database hits found for mass spectra of the distillate, the whisky from the same distillery and a wood extract.

**Sample type**	**Ion signals (S/N > 6)**	**Signals assigned to elemental formulae containing C,H,O,N, and S**	**Database annotations**
Distillate	1327	939	182 (19%)
Wood extract	3202	1537	391 (25%)
Whisky	5969	3015	791 (26%)

### Impact of wood maturation on whisky signatures

**Figure 2:**
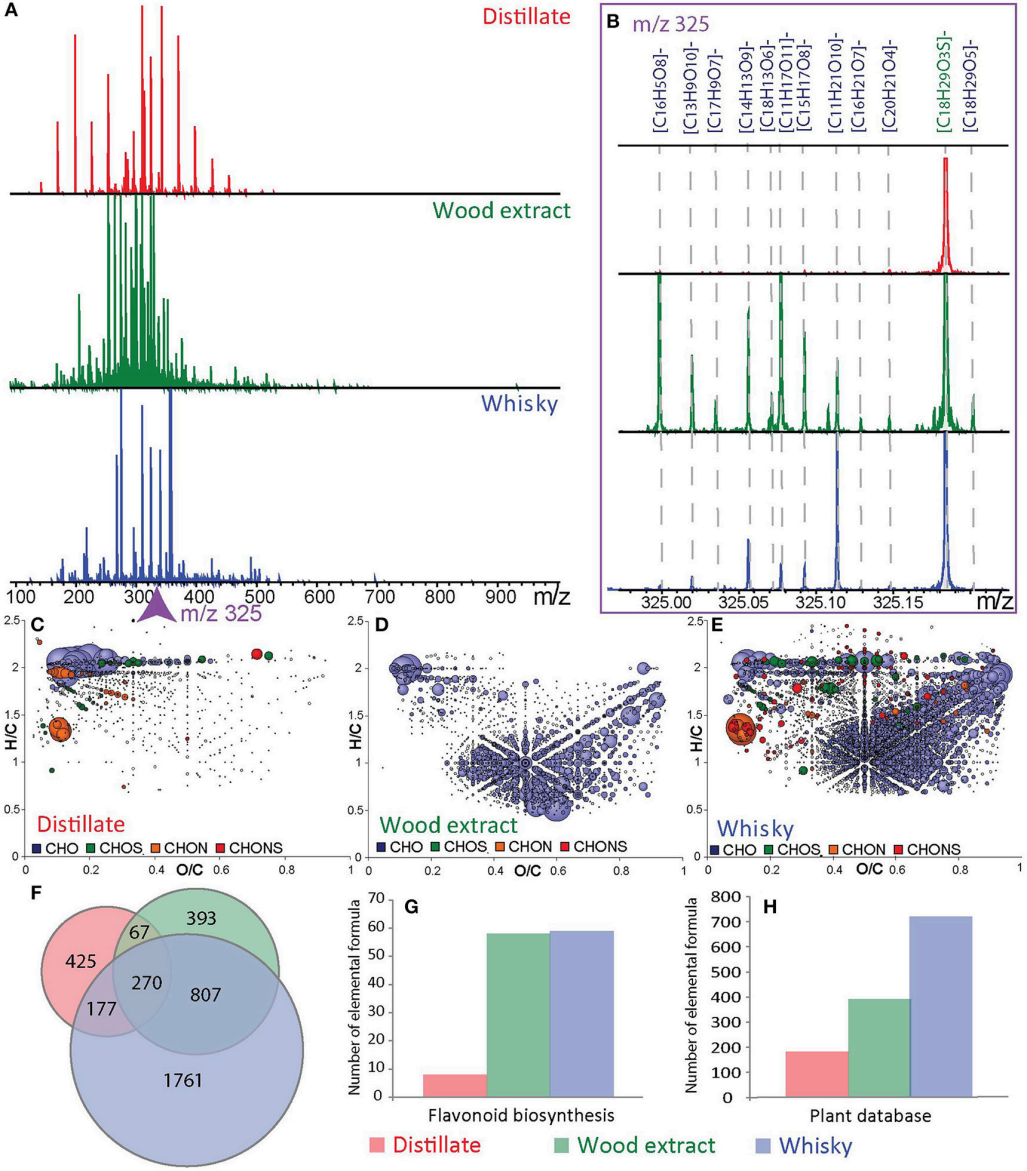
ESI (-) FT-ICR-MS spectra from a distillate, wood extract and whisky sample **(A)** in the mass range from 100 to 1,000 Da. Enlargements of the nominal mass *m/z* 325 **(B)** with molecular formulas assignments. Van Krevelen diagram (H/C vs. O/C atomic ratio) of the distillate **(C)**, wood extract **(D)**, and whisky **(E)**. The common and uncommon features from the distillate, wood extract and whisky are represented in a Venn diagram **(F)**. Histograms of annotated data in the flavonoid biosynthesis pathway **(G)** and in a home-build plant database **(H)**. Points in the van Krevelen diagrams are colored according to their elemental composition, CHO in blue, CHOS in green, CHON in orange, and CHONS in red, and scaled according to their relative peak intensities.

Wood contains a remarkable diversity of extractable organic compounds(Gougeon et al., 2009a,b), which can be potentially transferred to the distillate, including ellagitannins, lactones, coumarins, polysaccharides, hydrocarbons and fatty acids, terpenes, steroids, carotenoids, and furans (Rosso et al., 2008). In order to highlight the impact of barrel aging on the whisky composition, a fresh distillate (1 day old new-make spirit) and a matured whisky after 6 years of aging in a wood barrel, both from the same distillery, together with a toasted wood extract, were analyzed by FT-ICR-MS (Figure 2). The FT-ICR-MS spectrum and corresponding van Krevelen diagram of the distillate appeared to be relatively poor in signals compared to the very high diversity of both the wood extract and the whisky (Figure 2). Table 1 compares the number of signals found in the different spectra with the signals which could be assigned to elemental formulae containing C, H, O, N, and S, and those annotated with metabolites from databases. Van Krevelen diagrams of the distillate, wood extract, and whisky (Figures 2C–E) further highlight differences and similarities between the samples' compositions. The distillate showed many compounds with high intensities of higher alcohols (Figure 2C). In contrast, the wood extract revealed a high compound density in the area where polyphenols (flavonols, ellagitannins) and carbohydrates are anticipated (Figure 2D). The Whisky composition appeared more diverse supposing a high content of flavonols, ellagitannins, sugars, amino acids, and alcohols (Figure 2E). Before contact with in the oak barrels, the distillate already contains some of the compounds, which contribute to the final whisky flavorand some nitrogen and sulfur containing compounds. But as for many alcoholic beverages the influence of oak through aging in barrel is overriding, and the high alcohol content of the distillate certainly allows for the extraction of a large number of compounds from the wood barrel during maturation. As shown in the Venn diagram (Figure 2F), 1,077 elemental formulas (41% of the formulas found in the whisky) were found in both the wood extract and the whisky sample. On the opposite only 447 elemental formulas (15% of the whisky formulas) were found in the distillate and the whisky sample. However, a large number of elemental formulas present in the whisky were not present in the wood extract or in the distillate. This highlights the molecular diagenesis occurring during maturation. In total 1,761 elemental formulas were uniquely found in the whisky sample (Figures 2E,F), which can be the result of several processes. Extracted compounds from wood can certainly be involved in chemical reactions, but wood sorption of whisky compounds may also happen (Barrera-García et al., 2007; Canas et al., 2007; Rosso et al., 2008).

The number and the type of suggested candidates from the flavonoid metabolites pathway found in the wood extract and the whisky sample were very close. Thus, the number of total annotations found in database increased from the distillate samples to the wood extract samples and finally to the whisky samples (Figures 2G,H and Table 1). However, on the basis of existing databases, most of the detected metabolites observed in this study remain unknown (Table 1).We used LC-MS/MS for further structural confirmation of ellagic and gallic acid found in whiskies (Supplemental Figure 2). Barrel related markers are proposed in Table 2, including syringic acid, caffeic acid, catechin, and epigallocatechin. The low concentration of these compounds in whisky samples was not sufficient to detect them in an LC-MS/MS experiment compared to FT-ICR-MS.

**Table 2:** Subset of exact masses of the first and second (^13^C) isotope present in SB and/or BB barrelwood and whisky samples FT-ICR-MS spectra, which could be assigned to elemental formulae containing C, H, and O with associated absolute errors of assignment in ppm and possible compound annotation based on our database.

***m/z***	***m/z*^13^C isotope**	**Error (ppm)**	**Formula [M-H]^−^**	**Possible annotation**
179.03498	< S/N 6	0.00	C_9_H_7_${\text{O}}_{4}^{-}$	Caffeic acid
181.05062	182.05397	0.01	C_9_H_9_${\text{O}}_{4}^{-}$	Syringaldehyde
193.05064	194.05399	0.06	C_10_H_9_${\text{O}}_{4}^{-}$	Ferulic acid
195.06627	169.06963	0.09	C_10_H_11_${\text{O}}_{4}^{-}$	Ethyl vanillate
197.04555	198.04889	0.16	C_9_H_9_${\text{O}}_{5}^{-}$	Syringic acid
289.07176	290.07513	0.02	C_15_H_13_${\text{O}}_{6}^{-}$	Catechin
301.03538	>S/N 6	0.00	C_15_H_9_${\text{O}}_{7}^{-}$	Quercetin
457.07762	458.08072	0.02	C_22_H_17_${\text{O}}_{11}^{-}$	Epigallocatechin
463.0882	464.09158	0.01	C_21_H_19_${\text{O}}_{12}^{-}$	Isoquercetin
477.06742	478.07086	0.08	C_21_H_17_${\text{O}}_{13}^{-}$	Quercetin-glucuronide
479.08311	480.0864	0.01	C_21_H_19_${\text{O}}_{13}^{-}$	Myricetin-glucoside

### Impact of barrel history on whisky chemical spaces

For the making of whisky, the barrel can be new or previously used for wine aging (Piggott et al., 1993). In Europe, most whiskies are made using barrels, which had previously contained Bourbon (BB) or Sherry (SB). In order to highlight the influence of the barrel history, nine whiskies aged in BB and nine whiskies aged in SB were analyzed by FT-ICR-MS. Within each group, whiskies came from different distilleries at various ages and alcohol strengths (Supplemental Table 1).

**Figure 3:**
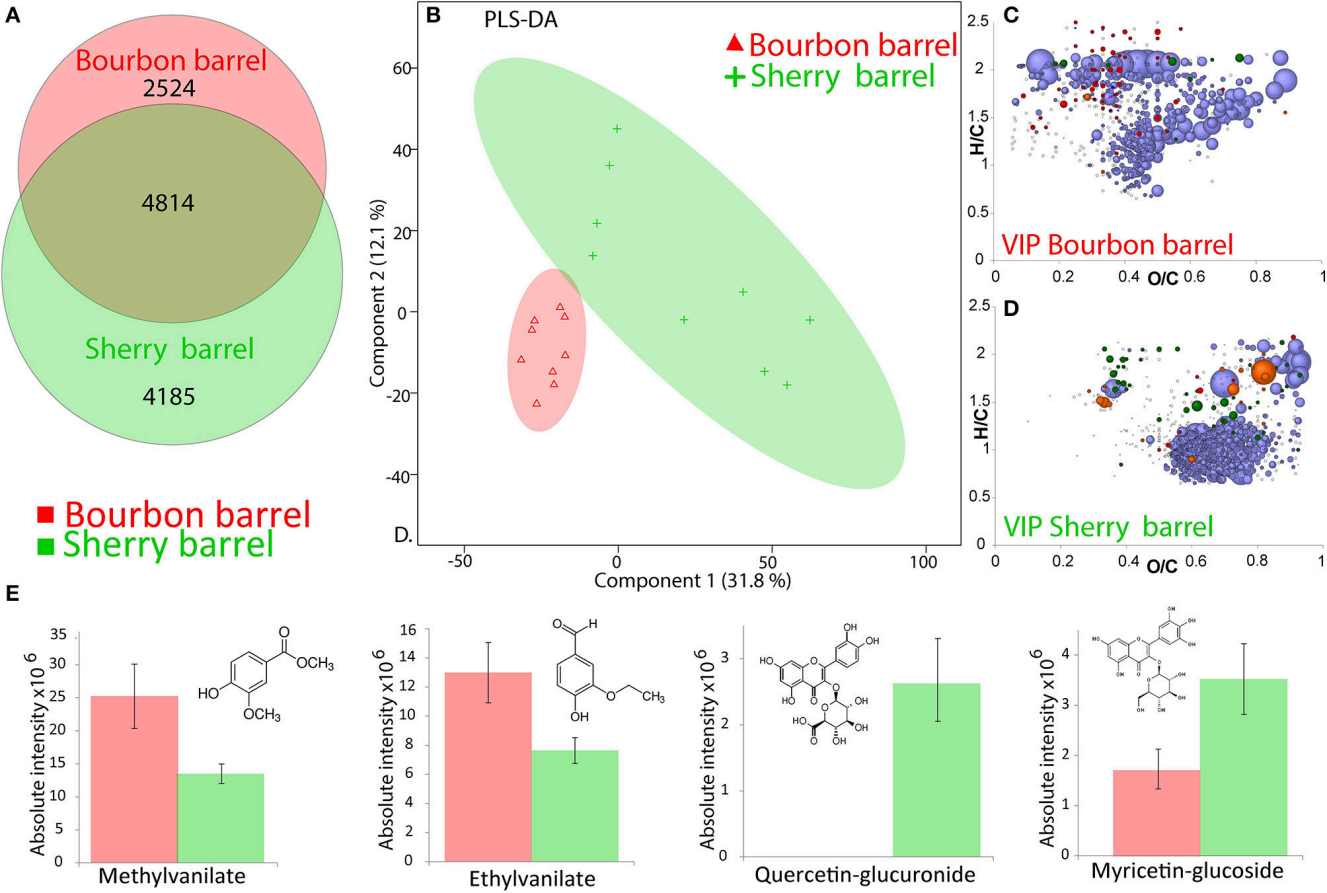
Visualization of the FT-ICR-MS data from Whiskies matured in Bourbon barrels (BB) and Sherry barrels (BB). **(A)** Common and unique features from SB and BB Whiskies, represented in a Venn diagram. **(B)** PLS-DA score plot for a model based on nine BB samples and nine SB samples (R2Y = 0.7 and Q2 = 0.4), the ellipses are showing the confidence intervals at 95%, the two first components explained 43.9% of the total variability. Van Krevelen diagrams for the “VIP” features extracted from the PLS-DA for whisky matured in **(C)** BB and **(D)** SB. **(E)** Bar chart of the absolute intensity of some wood biomarkers significantly more present either in BB Whiskies (red) or in SB Whiskies (green) with a *p* < 0.05. Points in van Krevelen diagrams are colored according to their elemental composition, CHO in blue, CHOS in green, CHON in orange, and CHONS in red, scaling according to their relative peak intensity.

According to the Venn diagram (Figure 3A), up to 4,814 elemental formulas were found in both whiskies aged in BB (representing 65% of the total elemental formulas annotated for these whiskies) and to whiskies aged in SB (representing 53% of the total elemental formulas annotated for these whiskies). Unique formulas were thus much higher for SB whiskies (4,185) than for BB whiskies (2,524). The PLS-DA score plot (Figure 3B) highlights that BB whiskies and SB whiskies could be separated using the first two components (explaining 43.9% of the variability; R2Y = 0.7 and Q2 = 0.4). Interestingly, our statistical analysis suggested a higher chemical diversity among SB whiskies, which is in contrast with results published by Kew et al. (2016). However, Kew et al. considered whisky samples made from malt only whereas our set of samples included grain and malt whiskies. Therefore, our results further show that ultra-high resolution MS data maintain chemical patterns which enabled the discrimination of whiskies according to the history of barrel used for the maturation, regardless of the cereal sources.

For each type of barrel, variable importance in projection (“VIP”) features filtered from the PLS-DA analysis were projected into a van Krevelen diagram (Figures 3C,D). “VIP” scores estimate the importance of each mass in the projection used in the PLS-DA model. Six hundred and ninety seven elemental formulas were significantly (*p* < 0.05) higher in intensities in BB whiskies and 1,346 in SB whiskies (Supplemental Table 3). Consistently with Kew et al. BB “VIP” features were not only found in the area of polyphenols (flavonols and oligolignols) but also in the area of saturated fatty acids, whereas SB “VIP” features were mostly in the area of higher oxidized polyphenols and carbohydrates (Figures 1D, 3C,D).

Only 257 masses (out of 1,346) from the SB“VIP” listed found hits in database (Supplemental Table 3). Most of these identifications were polyphenol glycosides. As it can be observed in the signal abundance from the van Krevelen diagrams in the transition zone between phenolic compounds and carbohydrates, glycoside conjugation could be an important chemical transformation related to SB maturation. Among the features, which were significantly more abundant in BB whiskies, we annotated esters such as methylvanilate or ethylvanilate (Figure 3E), which are wood markers (Pérez-Coello et al., 1998; Vichi et al., 2007). Elemental formulas corresponding to quercetin-glucuronide and myricetin-glucoside were found significantly more present in SB whiskies (*p* < 0.005). Interestingly, such barrel-related compounds would be actually wine biomarkers (Wightman et al., 1997; Villiers et al., 2005), which is consistent with the history of SB casks. Unfortunately, such compounds were not concentrated enough in our whisky samples to permit the structural identification by LC-MS/MS.

### The distillate nature and its relation to the final wood matured spirit: whisky vs. rum

**Figure 4:**
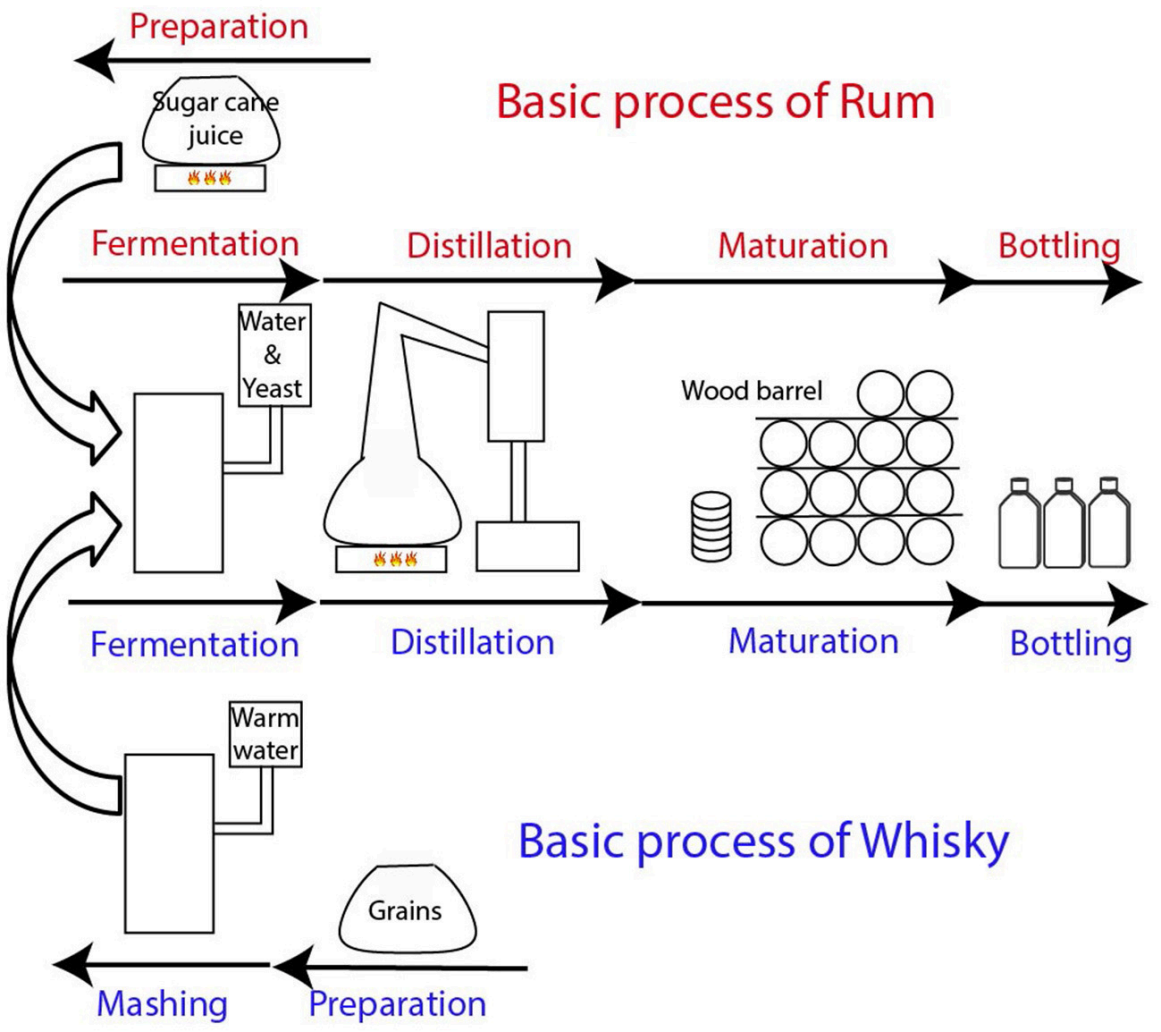
Representation of the basic elaboration process of rum and whisky.

Based on recent results, including the comparison of the new made spirit with mature whisky samples, it is obvious that the complexity of mature whisky mainly comes from the maturation in oak wood barrel (Kew et al., 2016). We hypothesized that other spirits matured in wood barrels, such as rum, exhibit a comparable chemical complexity reported for whisky. The production of whisky involves malting, where the barley is soaked in water and dried by heating, mashing (combining milled grain with hot water), fermentation after yeast addition, distillation in copper stills, maturation in oak barrel and bottling (Figure 4; Lea and Piggott, 2003; Agu et al., 2006; Russell and Stewart, 2014). The largest part of the whisky production process is comparable with rum production (Lea and Piggott, 2003). Rum production begins with sugar cane juice. The sugar cane juice can be directly fermented and distilled as practiced in Martinique and Guadalupe or concentrated into syrup or into molasses before fermentation and distillation (Arroyo, 1945; Fahrasmane and Ganou-Parfait, 1997; Lea and Piggott, 2003). Next steps are comparable with whisky processing including fermentation, distillation, maturation in wood barrel and bottling (Figure 4).

**Figure 5:**
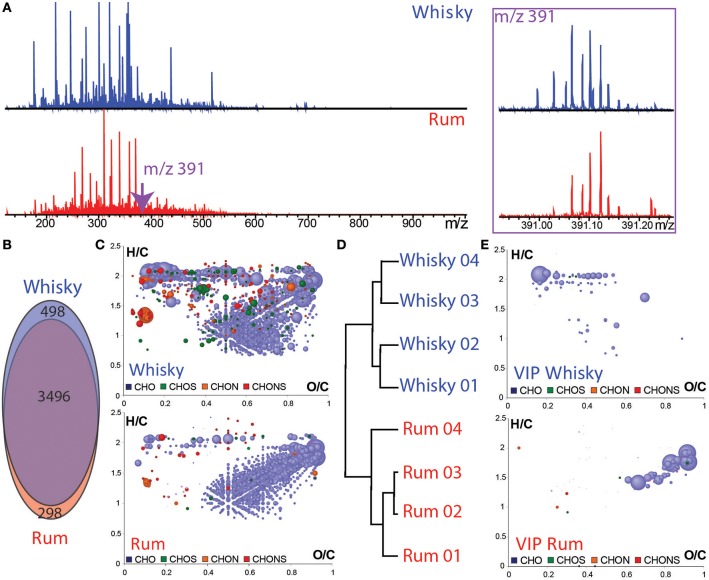
Raw FT-ICR-MS mass spectra of whisky and rum samples **(A)** in the mass range from *m/z* 100–1000 Da and enlargement of the nominal mass *m/z* 391. **(B)** Common and unique features found for whiskies and rums. **(C)** Van Krevelen diagrams (H/C vs. O/C atomic ratio) of the whisky and rum samples. **(D)** Hierarchical cluster analysis of whiskies and rums. Points in van Krevelen diagrams are colored according to their elemental composition, CHO in blue, CHOS in green, CHON in orange, and CHONS in red. Scaling according to the peak intensity. **(E)** Van Krevelen diagrams for the “VIP” features, which contribute to the HCA discrimination of whisky and rum samples.

To compare the chemical spaces of whisky with those of rum, four whiskies and four rums from various distilleries, ages, origins, and alcoholic percentages were analyzed by FT-ICR-MS. Spectrum demonstrates the chemical complexity with 3,994 peaks observed for whisky and 3,794 peaks for rum samples. Whiskies seemed to be relatively richer in small molecules (Figure 5A), predominantly more oxygenated compounds were observed (Figure 5A). Up to 12 peaks could be detected at *m/z* 391 for whiskies, and only 8 for rums. However, most peaks (3,496) were found in whisky and rum samples (Figure 5B). Van Krevelen diagrams, individual or each sample group revealed that whiskies and rums have a close CHO signature covering the polyphenol area (Figure 5C). However, whiskies clearly appeared to be richer in CHO high alcohols and fatty acids (Figure 5C). Despite the similarities between the two spirits as shown by the van Krevelen diagram and the high percentage of common masses, it is clear that there are differences amongst samples. A hierarchical cluster analysis of such high-dimensional MS data straightforwardly provided an excellent separation of the two spirits (Figure 5D). Van Krevelen diagrams revealed the specific distributions of elemental composition that are present in both distillate but in significantly higher intensity in whisky or in rum (Figure 5E). The specificity of whisky is characterized in particular by the presence of CHO compounds especially in the fatty acid and high alcohol area whereas rum is characterized by CHO elemental formulas in the carbohydrate area and Maillard reaction area of van Krevelen diagrams (Figure 5E; Hemmler et al., 2017).

## Conclusion

The combination of ultrahigh-resolution mass spectrometry (FT-ICR-MS and LC-MS/MS) and statistical analysis allowed differentiation and characterization of whisky samples regardless of their origins, with particular emphasis on the impact of the wood casks history before they were used for the maturation.

The impact of the wood and barrel history on the distillate's composition during maturation was revealed by the comparison of whiskies samples with new-make distillate and wood extract. We show that the influence of oak through aging in cask is overriding, as highlight by the high percentage of features found in both the wood extract and the whisky sample. Potential chemical markers corresponding to barrel history maturation have been extracted, enabled the discrimination of whiskies according to the history of barrel used for the maturation, regardless of the impact of others factors (distillery, aging, and environmental). Ellagic acid and gallic acid were likely examples as wood maturation markers, among the few annotated masses confirmed by LC-MS/MS analysis and comparison with standard compounds.

Our results further demonstrated that the large number of elemental formulas present in the whisky and not present in the wood extract or in the distillate point out the molecular diagenesis occurring during maturation. We also showed that the comparison of rum and whisky spirits revealed the specific whisky signature, highlighting the importance of the initial composition of the distillate and the distillery processes.

The combination of omics techniques showed great potential for the analysis of Whisky and could be used in future works for the investigation of distillery, origin, or aging impact on the whisky.

## Author contributions

CR-G, BS, RDG, and PS-K: Designed the research; CR-G, JS, DH, MW, BK, and PS-K: Performed the experiments and analyzed the data; CR-G, JS, DH, MW, BK, BS, RDG, and PS-K: Wrote the manuscript.

### Conflict of interest statement

The authors declare that the research was conducted in the absence of any commercial or financial relationships that could be construed as a potential conflict of interest.

## Supplementary material

Supplemental Figure 1 Evaluation of the repeatability of FT-ICR-MS analysis overtime. Comparison of spectra between *m/z* 100 and 600 Da and at nominal masses *m/z* 321 for the ten QC samples injected every ten samples during the running time. PCA scores plot of FT-ICR-MS of the 106 whiskies samples (gray) and the 10 QCs (red) showing the very high similarity of QCs. Coefficient of variation of intensities of formulas detected among the ten injections of QC sample and frequency of the common detected formulas over the 10 QCs (all the ten QC samples in green, higher than in 5 samples in blue and lower than 5 samples in gray).

Supplemental Figure 2 UV-Vis spectra of ellagic acid and gallic acid standards, and comparison of MS/MS spectra (10, 20, and 40 eV) from whisky, ellagic acid, and gallic acid standards.

Supplemental Table 1 Tables of the 150 whiskies samples.

Supplemental Table 2 Tables of the 4 rums samples.

Supplemental Table 3 Key features discriminating Bourbon (BB—Figure 3C) and Sherry (SB—Figure 3D) cask matured whiskies based on PLS-DA (Figure 3B).
