# Supplementary figures and images for: Usage of FT-ICR-MS Metabolomics for Characterizing the Chemical Signatures of Barrel-Aged Whisky
Source: Front Chem. 2018 Feb 22;6:29. doi: 10.3389/fchem.2018.00029 (PMC5827162; doi:10.3389/fchem.2018.00029)

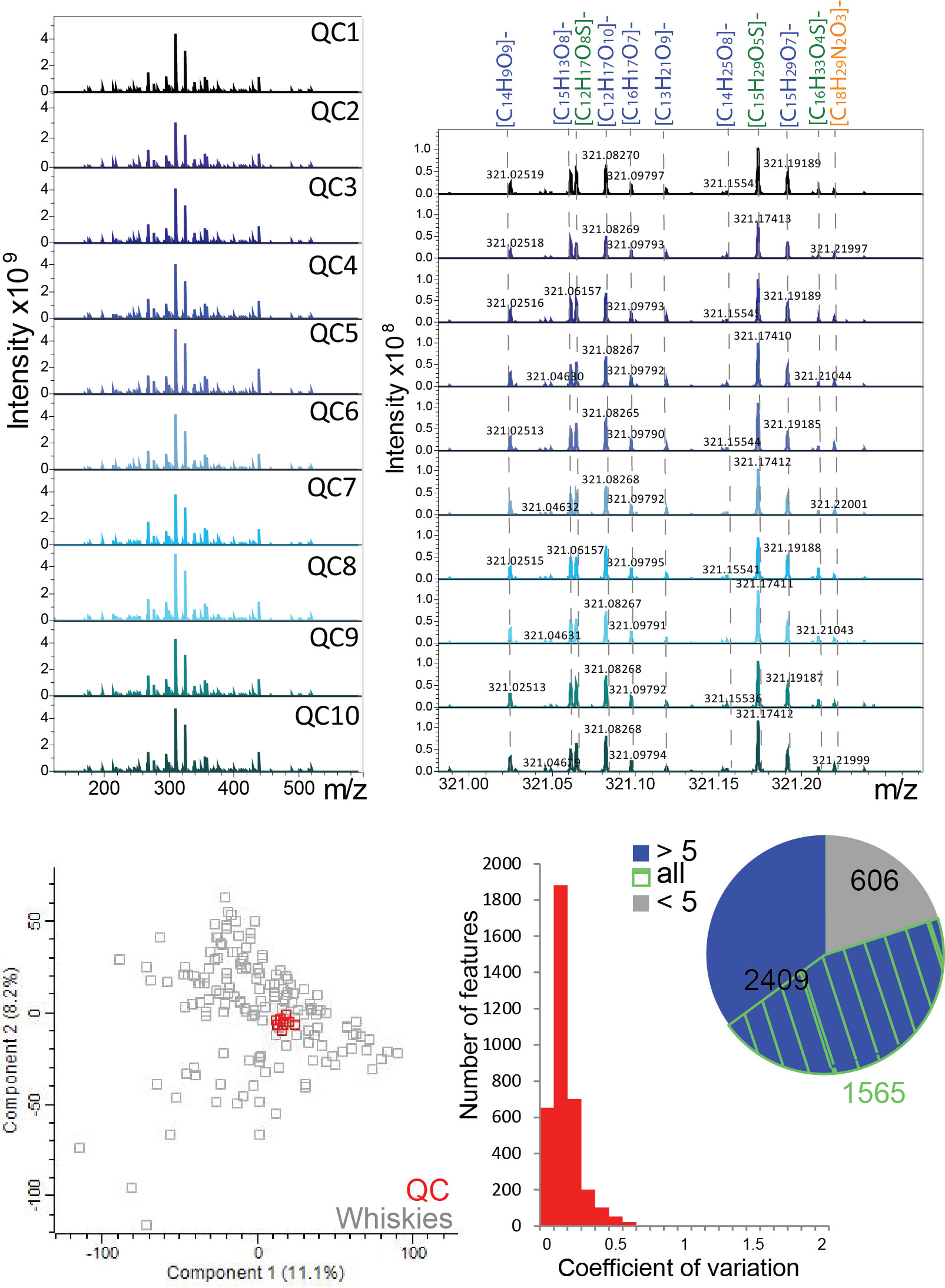

Supplement: Supplemental Figure 1 — Evaluation of the repeatability of FT-ICR-MS analysis overtime. Comparison of spectra between m/z 100 and 600 Da and at nominal masses m/z 321 for the ten QC samples injected every ten samples during the running time. PCA scores plot of FT-ICR-MS of the 106 whiskies samples (gray) and the 10 QCs (red) showing the very high similarity of QCs. Coefficient of variation of intensities of formulas detected among the ten injections of QC sample and frequency of the common detected formulas over the 10 QCs (all the ten QC samples in green, higher than in 5 samples in blue and lower than 5 samples in gray). [file Image1.JPEG]

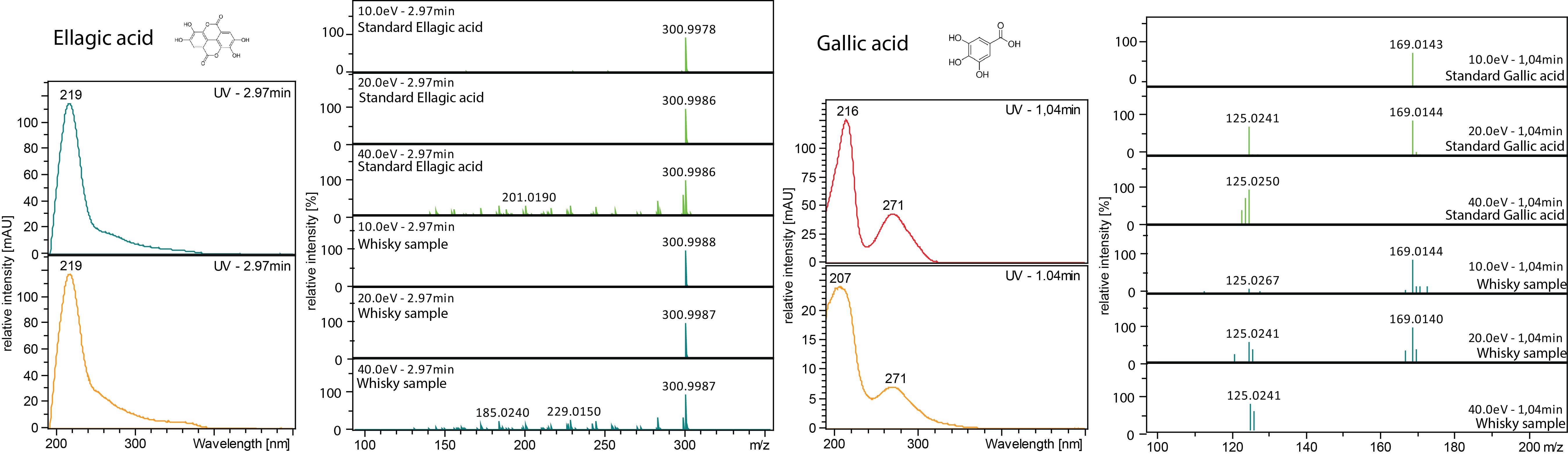

Supplement: Supplemental Figure 2 — UV-Vis spectra of ellagic acid and gallic acid standards, and comparison of MS/MS spectra (10, 20, and 40 eV) from whisky, ellagic acid, and gallic acid standards. [file Image2.JPEG]
