# Supplementary material for: Usage of FT-ICR-MS Metabolomics for Characterizing the Chemical Signatures of Barrel-Aged Whisky
Source: Front Chem. 2018 Feb 22;6:29. doi: 10.3389/fchem.2018.00029 (PMC5827162; doi:10.3389/fchem.2018.00029)
Supplement: Supplemental Table 1 — Tables of the 150 whiskies samples. [file Table1.DOCX]

Supplemental Table 1: Tables of the 150 whiskies samples

| **Distillery** | **Country** | **Region** | **Age** | **V%** | **Casks type** |
| --- | --- | --- | --- | --- | --- |
| 1 | Scotland | Speyside | 6 | 40 | - |
| 1 | Scotland | Speyside | 12 | 46 | - |
| 1 | Scotland | Speyside | 12 | 48 | - |
| 1 | Scotland | Speyside | 12 | 48 | - |
| 2 | Scotland | Islay | 6 | 58.3 | - |
| 2 | Scotland | Islay | 8 | 56.2 | - |
| 2 | Scotland | Islay | 9 | 54.1 | - |
| 2 | Scotland | Islay | 10 | 46 | - |
| 2 | Scotland | Islay | 12 | 40 | - |
| 3 | Scotland | Highlands | Blend | 46 | - |
| 4 | Scotland | Highlands | Blend | 58.1 | - |
| 5 | Scotland | Lowlands | 12 | 43 | - |
| 5 | Scotland | Lowlands | 12 | 40 | Bourbon and sherry casks |
| 5 | Scotland | Lowlands | 12 | 43 | Bourbon and sherry casks |
| 5 | Scotland | Lowlands | 12 | 40 | Bourbon and sherry casks |
| 5 | Scotland | Lowlands | 12 | 43 | Bourbon and sherry casks |
| 5 | Scotland | Lowlands | 18 | 40 | - |
| 5 | Scotland | Lowlands | 18 | 43 | Bourbon casks |
| 5 | Scotland | Lowlands | 18 | 43 | Bourbon casks |
| 5 | Scotland | Lowlands | 21 | 43 | Bourbon and sherry casks |
| 5 | Scotland | Lowlands | 21 | 43 | Bourbon and sherry casks |
| 5 | Scotland | Lowlands | Blend | 40 | various |
| 5 | Scotland | Lowlands | Blend | 40 | various |
| 6 | Scotland | Speyside | 14 | 47 | Finished in carridean rum casks |
| 7 | USA |  | 8 | 40 | new casks |
| 8 | Germany |  | Blend | 40 | - |
| 9 | Scotland | Islay | 12 | 40 | - |
| 9 | Scotland | Islay | 12 | 43 | Sherry casks |
| 9 | Scotland | Islay | 12 | 40 | various |
| 9 | Scotland | Islay | 12 | 43 | Sherry casks |
| 9 | Scotland | Islay | 12 | 40 | - |
| 9 | Scotland | Islay | 15 | 43 | various |
| 9 | Scotland | Islay | 15 | 43 | Bourbon and sherry casks |
| 9 | Scotland | Islay | 15 | 43 | various |
| 9 | Scotland | Islay | 15 | 43 | Bourbon and sherry casks |
| 9 | Scotland | Islay | 15 | 43 | Bourbon and sherry casks |
| 9 | Scotland | Islay | 17 | 43 | various |
| 9 | Scotland | Islay | 17 | 43 | various |
| 9 | Scotland | Islay | 18 | 43 | Bourbon and sherry casks |
| 9 | Scotland | Islay | 18 | 43 | Sherry casks |
| 9 | Scotland | Islay | Blend | 40.5 | Sherry casks |
| 9 | Scotland | Islay | Blend | 40 | Bourbon casks |
| 9 | Scotland | Islay | Blend | 40 | Bourbon casks |
| 10 | Scotland | Islay | Blend | 50 | - |
| 11 | Scotland | Lowlands | 21 | 46 | Sherry casks |
| 12 | Scotland | Lowlands | Blend | 40 | - |
| 13 | Scotland | Speyside | Blend | 40 | - |
| 14 | Scotland | Speyside | 12 |  | - |
| 15 | Scotland | Highlands | 14 | 46 | - |
| 15 | Scotland | Highlands | 14 | 46 | - |
| 15 | Scotland | Highlands | 15 | 46 | - |
| 15 | Scotland | Highlands | 1 day | 68 | - |
| 16 | Scotland | Islay | 12 | 43 | - |
| 17 | France |  | Blend | 43 | Sherry casks |
| 17 | France |  | Blend | 54 | Sherry casks |
| 17 | France |  | Blend | 43 | Sherry casks |
| 18 | Scotland | Speyside | 12 | 40 | - |
| 19 | USA |  | 12 | 47 | new casks |
| 19 | USA |  | 12 | 47 | new casks |
| 20 | Scotland | Highlands | 12 | 40 | - |
| 21 | Scotland | Speyside | 12 | 43 | - |
| 22 | Scotland | Highlands | 8 | 40 | Bourbon casks |
| 22 | Scotland | Highlands | 8 | 40 | Bourbon casks |
| 22 | Scotland | Highlands | 12 | 43 | Bourbon and Sherry casks |
| 22 | Scotland | Highlands | 12 | 43 | Bourbon and Sherry casks |
| 22 | Scotland | Highlands | 12 | 40 | - |
| 22 | Scotland | Highlands | 15 | 43 | various |
| 22 | Scotland | Highlands | 15 | 43 | various |
| 22 | Scotland | Highlands | 21 | 43 | various |
| 22 | Scotland | Highlands | 21 | 43 | various |
| 23 | Scotland | Speyside | 12 | 40 | - |
| 23 | Scotland | Speyside | 15 | 40 | - |
| 23 | Scotland | Speyside | 18 | 40 | - |
| 23 | Scotland | Speyside | 21 | 40 | - |
| 23 | Scotland | Speyside | 30 | 40 | - |
| 23 | Scotland | Speyside | Blend | 46 | - |
| 23 | Scotland | Speyside | Blend | 46 | - |
| 23 | Scotland | Speyside | Blend | 43 | - |
| 23 | Scotland | Speyside | Blend | 57.1 | - |
| 23 | Scotland | Speyside | Blend | 43 | - |
| 24 | Scotland | Lowlands | 10 | 43 | - |
| 24 | Scotland | Lowlands | 12 | 43 | - |
| 25 | Scotland | Highlands | 10 | 46 | Bourbon casks |
| 25 | Scotland | Highlands | 10 | 40 | - |
| 25 | Scotland | Highlands | 12 | 46 | Bourbon casks |
| 25 | Scotland | Highlands | Blend | 57.1 | Sherry casks |
| 25 | Scotland | Highlands | Blend | 46 | Sherry casks |
| 25 | Scotland | Highlands | Blend | 46 | Sherry casks |
| 25 | Scotland | Highlands | Blend | 43 | - |
| 26 | Scotland | Speyside | 27 | 55.6 | - |
| 27 | USA |  | 12 | 40 | new casks |
| 28 | Scotland | Highlands | 18 | 40 | - |
| 28 | Scotland | Highlands | Blend | 40 | - |
| 28 | Scotland | Highlands | Blend | 43 | - |
| 28 | Scotland | Highlands | Blend |  | - |
| 29 | Scotland | Islay | 12 | 48 | Sherry casks |
| 29 | Scotland | Islay | 16 | 43 | Sherry casks |
| 29 | Scotland | Islay | 16 | 43 | - |
| 29 | Scotland | Islay | 16 | 43 | - |
| 29 | Scotland | Islay | 21 | 56.5 | Sherry casks |
| 30 | Scotland | Islay | 10 | 40 | - |
| 30 | Scotland | Islay | 10 | 43 | - |
| 30 | Scotland | Islay | 12 | 53.1 | Sherry casks |
| 30 | Scotland | Islay | 25 | 40 | Bourbon and sherry casks |
| 30 | Scotland | Islay | 30 | 43 | - |
| 30 | Scotland | Islay | 31 | 49.7 | Sherry casks |
| 30 | Scotland | Islay | Blend | 43 | Sherry casks |
| 30 | Scotland | Islay | Blend | 43 | - |
| 31 | Scotland | Speyside | 7 | 40 | - |
| 31 | Scotland | Speyside | 12 | 40 | Sherry and bourbon casks |
| 31 | Scotland | Speyside | 12 | 40 | various |
| 31 | Scotland | Speyside | 18 | 43 | Sherry |
| 31 | Scotland | Speyside | 43 | 52.9 | Sherry casks |
| 32 | USA |  | 2 | >65 | new casks |
| 32 | USA |  | 1 day | >65 | new casks |
| 32 | USA |  | Blend | 45 | new casks |
| 33 | Scotland | Lowland | 6 | 40 | various |
| 33 | Scotland | Highland | 6 | 40 | various |
| 33 | Scotland | Speyside | 6 | 40 | various |
| 33 | Scotland | Islay | 6 | 40 | various |
| 33 | Scotland | Lowland | 6 | 40 | various |
| 33 | Scotland | Highland | 6 | 40 | various |
| 33 | Scotland | Speyside | 6 | 40 | various |
| 33 | Scotland | Islay | 6 | 40 | various |
| 34 | Scotland | Highland | 14 | 43 | - |
| 35 | Scotland | Speyside | 18 | 43 | - |
| 36 | Austria |  | 12 | 48 | - |
| 37 | Scotland | Highlands | 14 | 40 | - |
| 38 | Scotland | speyside | 12 | 40 | - |
| 38 | Scotland | Highlands | 12 | 40 | - |
| 38 | Scotland | Speyside | 12 | 40 | - |
| 39 | Scotland | Lowlands | Blend | 46 | - |
| 40 | Scotland | Speyside | 12 | 43 | - |
| 41 | Scotland | Highlands | Blend | 38 | - |
| 42 | Scotland | Highland | 3 | 60 | - |
| 42 | Scotland | Highland | 8 | 60 | - |
| 42 | Scotland | Highland | 10 | 60 | - |
| 42 | Scotland | Highlands | 18 | 45.8 | - |
| 42 | Scotland | Highlands | Blend | 57 | - |
| 43 | Scotland | Speyside | 15 | 50.4 | Sherry casks |
| 44 | Scotland | Speyside | 17 | 43 | Bourbon casks |
| 45 | Scotland | Speyside | 12 | 40 | Bourbon casks |
| 45 | Scotland | Speyside | 15 | 40 | Sherry casks |
| 45 | Scotland | Speyside | 16 | 57.7 | Bourbon casks |
| 45 | Scotland | Speyside | 18 | 43 | Bourbon and sherry casks |
| 46 | Scotland | Mull | 10 | 43 | - |
| 47 | USA |  | Blend | 50.5 | new casks |
| 48 | Canada |  | 10 | 40 | - |
| 49 | Japan |  | 18 | 40 | - |
| 49 | Japan |  | Blend | 60 | - |
