# Supplementary material for: Usage of FT-ICR-MS Metabolomics for Characterizing the Chemical Signatures of Barrel-Aged Whisky
Source: Front Chem. 2018 Feb 22;6:29. doi: 10.3389/fchem.2018.00029 (PMC5827162; doi:10.3389/fchem.2018.00029)
Supplement: Supplemental Table 2 — Tables of the 4 rums samples. [file Table2.DOCX]

**Supplementary table 2: Tables of the 4 rums samples**

| **Distillery** | **V%** | **Country** |
| --- | --- | --- |
| 1 | 40 | Barbades |
| 2 | 40 | Porto rico |
| 3 | 54 | France Martinique |
| 4 | 54 | Jamaique |
