# Supplementary material for: Usage of FT-ICR-MS Metabolomics for Characterizing the Chemical Signatures of Barrel-Aged Whisky
Source: Front Chem. 2018 Feb 22;6:29. doi: 10.3389/fchem.2018.00029 (PMC5827162; doi:10.3389/fchem.2018.00029)
Supplement: Supplemental Table 3 — Key features discriminating Bourbon (BB—Figure 3C) and Sherry (SB—Figure 3D) cask matured whiskies based on PLS-DA (Figure 3B). [file Table3.DOCX]

**Supplementary table 3: Key features discriminating Bourbon (BB – Figure 3C) and Sherry (SB – Figure 3D) cask matured whiskies based on PLS-DA (Figure 3B).**

| **m/z** | **H** | **C** | **O** | **N** | **S** | **P** | **Composition** | **Neutral mass** | **Cask** | **Number of hypothetical  annotation in databases** |
| --- | --- | --- | --- | --- | --- | --- | --- | --- | --- | --- |
| 133.014249 | 6 | 4 | 5 | 0 | 0 | 0 | CHO | 134.021525 | SB | 8 |
| 143.034984 | 8 | 6 | 4 | 0 | 0 | 0 | CHO | 144.04226 | SB | 12 |
| 143.107754 | 16 | 8 | 2 | 0 | 0 | 0 | CHO | 144.11503 | BB | 16 |
| 153.019334 | 6 | 7 | 4 | 0 | 0 | 0 | CHO | 154.02661 | SB | 8 |
| 161.045549 | 10 | 6 | 5 | 0 | 0 | 0 | CHO | 162.052825 | SB | 28 |
| 163.061199 | 12 | 6 | 5 | 0 | 0 | 0 | CHO | 164.068475 | BB | 33 |
| 169.123404 | 18 | 10 | 2 | 0 | 0 | 0 | CHO | 170.13068 | BB | 29 |
| 171.102669 | 16 | 9 | 3 | 0 | 0 | 0 | CHO | 172.109945 | BB | 13 |
| 173.009164 | 6 | 6 | 6 | 0 | 0 | 0 | CHO | 174.01644 | SB | 6 |
| 177.019334 | 6 | 9 | 4 | 0 | 0 | 0 | CHO | 178.02661 | BB | 4 |
| 179.05361 | 12 | 10 | 1 | 0 | 1 | 0 | CHOS | 180.060886 | SB | - |
| 181.071764 | 14 | 6 | 6 | 0 | 0 | 0 | CHO | 182.07904 | SB | 10 |
| 183.029899 | 8 | 8 | 5 | 0 | 0 | 0 | CHO | 184.037175 | BB | 7 |
| 183.102669 | 16 | 10 | 3 | 0 | 0 | 0 | CHO | 184.109945 | BB | 10 |
| 185.009164 | 6 | 7 | 6 | 0 | 0 | 0 | CHO | 186.01644 | SB | 12 |
| 185.081934 | 14 | 9 | 4 | 0 | 0 | 0 | CHO | 186.08921 | BB | 1 |
| 185.154704 | 22 | 11 | 2 | 0 | 0 | 0 | CHO | 186.16198 | BB | 18 |
| 187.061199 | 12 | 8 | 5 | 0 | 0 | 0 | CHO | 188.068475 | BB | 2 |
| 187.097584 | 16 | 9 | 4 | 0 | 0 | 0 | CHO | 188.10486 | BB | 4 |
| 187.133969 | 20 | 10 | 3 | 0 | 0 | 0 | CHO | 188.141245 | BB | 17 |
| 189.076849 | 14 | 8 | 5 | 0 | 0 | 0 | CHO | 190.084125 | BB | 7 |
| 191.019729 | 8 | 6 | 7 | 0 | 0 | 0 | CHO | 192.027005 | SB | 15 |
| 191.092499 | 16 | 8 | 5 | 0 | 0 | 0 | CHO | 192.099775 | BB | 8 |
| 193.035379 | 10 | 6 | 7 | 0 | 0 | 0 | CHO | 194.042655 | SB | 29 |
| 195.03327 | 12 | 6 | 5 | 0 | 1 | 0 | CHOS | 196.040546 | SB | 2 |
| 195.051029 | 12 | 6 | 7 | 0 | 0 | 0 | CHO | 196.058305 | SB | 13 |
| 196.97615 | 6 | 4 | 7 | 0 | 1 | 0 | CHOS | 197.983426 | SB | - |
| 197.012535 | 10 | 5 | 6 | 0 | 1 | 0 | CHOS | 198.019811 | SB | - |
| 197.081934 | 14 | 10 | 4 | 0 | 0 | 0 | CHO | 198.08921 | BB | 5 |
| 199.007055 | 8 | 8 | 4 | 0 | 1 | 0 | CHOS | 200.014331 | BB | - |
| 199.133969 | 20 | 11 | 3 | 0 | 0 | 0 | CHO | 200.141245 | BB | 9 |
| 202.072098 | 13 | 8 | 5 | 1 | 0 | 0 | CHNO | 203.079374 | SB | 29 |
| 205.050634 | 10 | 11 | 4 | 0 | 0 | 0 | CHO | 206.05791 | BB | 6 |
| 205.071764 | 14 | 8 | 6 | 0 | 0 | 0 | CHO | 206.07904 | BB | - |
| 205.159789 | 22 | 14 | 1 | 0 | 0 | 0 | CHO | 206.167065 | BB | 6 |
| 207.066284 | 12 | 11 | 4 | 0 | 0 | 0 | CHO | 208.07356 | BB | 4 |
| 207.087414 | 16 | 8 | 6 | 0 | 0 | 0 | CHO | 208.09469 | SB | - |
| 213.004079 | 6 | 8 | 7 | 0 | 0 | 0 | CHO | 214.011355 | SB | - |
| 213.040464 | 10 | 9 | 6 | 0 | 0 | 0 | CHO | 214.04774 | BB | 1 |
| 213.113234 | 18 | 11 | 4 | 0 | 0 | 0 | CHO | 214.12051 | BB | - |
| 215.030962 | 8 | 7 | 6 | 2 | 0 | 0 | CHNO | 216.038238 | SB | - |
| 215.056114 | 12 | 9 | 6 | 0 | 0 | 0 | CHO | 216.06339 | BB | - |
| 215.092499 | 16 | 10 | 5 | 0 | 0 | 0 | CHO | 216.099775 | BB | - |
| 215.165269 | 24 | 12 | 3 | 0 | 0 | 0 | CHO | 216.172545 | BB | 18 |
| 216.051363 | 11 | 8 | 6 | 1 | 0 | 0 | CHNO | 217.058639 | SB | 8 |
| 217.108149 | 18 | 10 | 5 | 0 | 0 | 0 | CHO | 218.115425 | BB | 5 |
| 217.144534 | 22 | 11 | 4 | 0 | 0 | 0 | CHO | 218.15181 | BB | - |
| 219.034137 | 16 | 9 | 0 | 0 | 3 | 0 | CHOS | 220.041413 | SB | - |
| 219.066284 | 12 | 12 | 4 | 0 | 0 | 0 | CHO | 220.07356 | BB | 3 |
| 219.175439 | 24 | 15 | 1 | 0 | 0 | 0 | CHO | 220.182715 | BB | 32 |
| 221.030294 | 10 | 7 | 8 | 0 | 0 | 0 | CHO | 222.03757 | SB | - |
| 221.045549 | 10 | 11 | 5 | 0 | 0 | 0 | CHO | 222.052825 | BB | 10 |
| 221.066679 | 14 | 8 | 7 | 0 | 0 | 0 | CHO | 222.073955 | SB | 6 |
| 222.040798 | 9 | 10 | 5 | 1 | 0 | 0 | CHNO | 223.048074 | SB | 13 |
| 223.028185 | 12 | 7 | 6 | 0 | 1 | 0 | CHOS | 224.035461 | SB | - |
| 223.082329 | 16 | 8 | 7 | 0 | 0 | 0 | CHO | 224.089605 | BB | - |
| 223.097584 | 16 | 12 | 4 | 0 | 0 | 0 | CHO | 224.10486 | BB | 1 |
| 224.056448 | 11 | 10 | 5 | 1 | 0 | 0 | CHNO | 225.063724 | SB | 4 |
| 225.061594 | 14 | 7 | 8 | 0 | 0 | 0 | CHO | 226.06887 | SB | - |
| 225.076849 | 14 | 11 | 5 | 0 | 0 | 0 | CHO | 226.084125 | BB | 8 |
| 225.113234 | 18 | 12 | 4 | 0 | 0 | 0 | CHO | 226.12051 | BB | 3 |
| 226.035713 | 9 | 9 | 6 | 1 | 0 | 0 | CHNO | 227.042989 | SB | 8 |
| 227.056114 | 12 | 10 | 6 | 0 | 0 | 0 | CHO | 228.06339 | BB | 2 |
| 227.077244 | 16 | 7 | 8 | 0 | 0 | 0 | CHO | 228.08452 | SB | - |
| 227.092499 | 16 | 11 | 5 | 0 | 0 | 0 | CHO | 228.099775 | BB | 3 |
| 227.201654 | 28 | 14 | 2 | 0 | 0 | 0 | CHO | 228.20893 | BB | 19 |
| 230.067013 | 13 | 9 | 6 | 1 | 0 | 0 | CHNO | 231.074289 | SB | 5 |
| 231.029899 | 8 | 12 | 5 | 0 | 0 | 0 | CHO | 232.037175 | BB | 2 |
| 231.051029 | 12 | 9 | 7 | 0 | 0 | 0 | CHO | 232.058305 | BB | - |
| 231.087414 | 16 | 10 | 6 | 0 | 0 | 0 | CHO | 232.09469 | BB | - |
| 231.123799 | 20 | 11 | 5 | 0 | 0 | 0 | CHO | 232.131075 | BB | - |
| 233.066679 | 14 | 9 | 7 | 0 | 0 | 0 | CHO | 234.073955 | BB | 2 |
| 235.045944 | 12 | 8 | 8 | 0 | 0 | 0 | CHO | 236.05322 | SB | - |
| 235.061199 | 12 | 12 | 5 | 0 | 0 | 0 | CHO | 236.068475 | BB | 2 |
| 235.082329 | 16 | 9 | 7 | 0 | 0 | 0 | CHO | 236.089605 | BB | - |
| 237.00745 | 10 | 7 | 7 | 0 | 1 | 0 | CHOS | 238.014726 | SB | - |
| 237.043835 | 14 | 8 | 6 | 0 | 1 | 0 | CHOS | 238.051111 | SB | 2 |
| 237.097979 | 18 | 9 | 7 | 0 | 0 | 0 | CHO | 238.105255 | BB | - |
| 237.186004 | 26 | 15 | 2 | 0 | 0 | 0 | CHO | 238.19328 | BB | 19 |
| 239.019729 | 8 | 10 | 7 | 0 | 0 | 0 | CHO | 240.027005 | SB | - |
| 239.0231 | 12 | 7 | 7 | 0 | 1 | 0 | CHOS | 240.030376 | SB | - |
| 239.059485 | 16 | 8 | 6 | 0 | 1 | 0 | CHOS | 240.066761 | BB | 10 |
| 239.128884 | 20 | 13 | 4 | 0 | 0 | 0 | CHO | 240.13616 | BB | - |
| 239.201654 | 28 | 15 | 2 | 0 | 0 | 0 | CHO | 240.20893 | BB | 20 |
| 240.033604 | 11 | 10 | 4 | 1 | 1 | 0 | CHNOS | 241.04088 | SB | 1 |
| 240.051363 | 11 | 10 | 6 | 1 | 0 | 0 | CHNO | 241.058639 | SB | 2 |
| 240.087748 | 15 | 11 | 5 | 1 | 0 | 0 | CHNO | 241.095024 | SB | - |
| 241.108149 | 18 | 12 | 5 | 0 | 0 | 0 | CHO | 242.115425 | BB | - |
| 243.087414 | 16 | 11 | 6 | 0 | 0 | 0 | CHO | 244.09469 | BB | - |
| 243.196569 | 28 | 14 | 3 | 0 | 0 | 0 | CHO | 244.203845 | BB | 15 |
| 245.012535 | 10 | 9 | 6 | 0 | 1 | 0 | CHOS | 246.019811 | SB | - |
| 245.066679 | 14 | 10 | 7 | 0 | 0 | 0 | CHO | 246.073955 | BB | - |
| 245.139449 | 22 | 12 | 5 | 0 | 0 | 0 | CHO | 246.146725 | BB | 2 |
| 245.175834 | 26 | 13 | 4 | 0 | 0 | 0 | CHO | 246.18311 | BB | - |
| 247.01293 | 12 | 5 | 9 | 0 | 1 | 0 | CHOS | 248.020206 | SB | - |
| 247.028185 | 12 | 9 | 6 | 0 | 1 | 0 | CHOS | 248.035461 | SB | 2 |
| 247.039418 | 12 | 8 | 5 | 2 | 1 | 0 | CHNOS | 248.046694 | SB | - |
| 247.082329 | 16 | 10 | 7 | 0 | 0 | 0 | CHO | 248.089605 | SB | - |
| 247.155099 | 24 | 12 | 5 | 0 | 0 | 0 | CHO | 248.162375 | BB | - |
| 248.02477 | 7 | 9 | 2 | 5 | 1 | 0 | CHNOS | 249.032046 | BB | 1 |
| 249.025209 | 10 | 8 | 9 | 0 | 0 | 0 | CHO | 250.032485 | SB | 1 |
| 249.097979 | 18 | 10 | 7 | 0 | 0 | 0 | CHO | 250.105255 | BB | - |
| 251.019729 | 8 | 11 | 7 | 0 | 0 | 0 | CHO | 252.027005 | SB | - |
| 251.0231 | 12 | 8 | 7 | 0 | 1 | 0 | CHOS | 252.030376 | SB | - |
| 251.040859 | 12 | 8 | 9 | 0 | 0 | 0 | CHO | 252.048135 | SB | - |
| 251.056114 | 12 | 12 | 6 | 0 | 0 | 0 | CHO | 252.06339 | BB | - |
| 251.077244 | 16 | 9 | 8 | 0 | 0 | 0 | CHO | 252.08452 | BB | - |
| 251.165269 | 24 | 15 | 3 | 0 | 0 | 0 | CHO | 252.172545 | BB | 11 |
| 252.087748 | 15 | 12 | 5 | 1 | 0 | 0 | CHNO | 253.095024 | SB | 2 |
| 252.095826 | 19 | 7 | 1 | 5 | 2 | 0 | CHNOS | 253.103102 | BB | - |
| 253.035379 | 10 | 11 | 7 | 0 | 0 | 0 | CHO | 254.042655 | SB | - |
| 253.056509 | 14 | 8 | 9 | 0 | 0 | 0 | CHO | 254.063785 | SB | - |
| 253.075135 | 18 | 9 | 6 | 0 | 1 | 0 | CHOS | 254.082411 | BB | 5 |
| 253.108149 | 18 | 13 | 5 | 0 | 0 | 0 | CHO | 254.115425 | BB | 2 |
| 254.030628 | 9 | 10 | 7 | 1 | 0 | 0 | CHNO | 255.037904 | SB | 6 |
| 254.067013 | 13 | 11 | 6 | 1 | 0 | 0 | CHNO | 255.074289 | SB | - |
| 255.014644 | 8 | 10 | 8 | 0 | 0 | 0 | CHO | 256.02192 | SB | - |
| 255.0544 | 16 | 8 | 7 | 0 | 1 | 0 | CHOS | 256.061676 | SB | 6 |
| 255.121295 | 20 | 17 | 0 | 0 | 1 | 0 | CHOS | 256.128571 | BB | - |
| 255.123799 | 20 | 13 | 5 | 0 | 0 | 0 | CHO | 256.131075 | BB | - |
| 255.196569 | 28 | 15 | 3 | 0 | 0 | 0 | CHO | 256.203845 | BB | 5 |
| 256.119048 | 19 | 12 | 5 | 1 | 0 | 0 | CHNO | 257.126324 | SB | - |
| 256.993909 | 6 | 9 | 9 | 0 | 0 | 0 | CHO | 258.001185 | SB | - |
| 257.066679 | 14 | 11 | 7 | 0 | 0 | 0 | CHO | 258.073955 | BB | - |
| 257.103064 | 18 | 12 | 6 | 0 | 0 | 0 | CHO | 258.11034 | BB | - |
| 257.139449 | 22 | 13 | 5 | 0 | 0 | 0 | CHO | 258.146725 | BB | - |
| 259.024814 | 8 | 13 | 6 | 0 | 0 | 0 | CHO | 260.03209 | BB | 3 |
| 259.082329 | 16 | 11 | 7 | 0 | 0 | 0 | CHO | 260.089605 | BB | - |
| 259.118714 | 20 | 12 | 6 | 0 | 0 | 0 | CHO | 260.12599 | BB | - |
| 259.191484 | 28 | 14 | 4 | 0 | 0 | 0 | CHO | 260.19876 | BB | 1 |
| 260.061155 | 11 | 11 | 1 | 5 | 1 | 0 | CHNOS | 261.068431 | BB | - |
| 260.077578 | 15 | 10 | 7 | 1 | 0 | 0 | CHNO | 261.084854 | SB | 4 |
| 261.00745 | 10 | 9 | 7 | 0 | 1 | 0 | CHOS | 262.014726 | SB | 1 |
| 261.025209 | 10 | 9 | 9 | 0 | 0 | 0 | CHO | 262.032485 | SB | - |
| 261.02858 | 14 | 6 | 9 | 0 | 1 | 0 | CHOS | 262.035856 | SB | 3 |
| 261.040464 | 10 | 13 | 6 | 0 | 0 | 0 | CHO | 262.04774 | BB | 2 |
| 261.043835 | 14 | 10 | 6 | 0 | 1 | 0 | CHOS | 262.051111 | SB | - |
| 261.055935 | 18 | 10 | 0 | 2 | 3 | 0 | CHNOS | 262.063211 | SB | 3 |
| 261.097979 | 18 | 11 | 7 | 0 | 0 | 0 | CHO | 262.105255 | BB | - |
| 261.134364 | 22 | 12 | 6 | 0 | 0 | 0 | CHO | 262.14164 | BB | - |
| 262.056843 | 13 | 9 | 8 | 1 | 0 | 0 | CHNO | 263.064119 | BB | - |
| 262.926045 | 4 | 6 | 4 | 2 | 3 | 0 | CHNOS | 263.933321 | BB | - |
| 262.935942 | 8 | 4 | 7 | 0 | 3 | 0 | CHOS | 263.943218 | BB | - |
| 262.943804 | 4 | 6 | 6 | 2 | 2 | 0 | CHNOS | 263.95108 | BB | - |
| 262.951197 | 8 | 8 | 4 | 0 | 3 | 0 | CHOS | 263.958473 | BB | - |
| 262.995444 | 8 | 11 | 2 | 2 | 2 | 0 | CHNOS | 264.00272 | BB | - |
| 262.998815 | 12 | 8 | 2 | 2 | 3 | 0 | CHNOS | 264.006091 | BB | - |
| 263.006208 | 16 | 10 | 0 | 0 | 4 | 0 | CHOS | 264.013484 | SB | - |
| 263.007845 | 12 | 5 | 10 | 0 | 1 | 0 | CHOS | 264.015121 | SB | - |
| 263.009181 | 8 | 6 | 6 | 4 | 1 | 0 | CHNOS | 264.016457 | SB | 7 |
| 263.019729 | 8 | 12 | 7 | 0 | 0 | 0 | CHO | 264.027005 | SB | - |
| 263.0231 | 12 | 9 | 7 | 0 | 1 | 0 | CHOS | 264.030376 | SB | 3 |
| 263.030962 | 8 | 11 | 6 | 2 | 0 | 0 | CHNO | 264.038238 | SB | - |
| 263.056114 | 12 | 13 | 6 | 0 | 0 | 0 | CHO | 264.06339 | BB | - |
| 263.064843 | 12 | 16 | 0 | 2 | 1 | 0 | CHNOS | 264.072119 | SB | - |
| 263.077244 | 16 | 10 | 8 | 0 | 0 | 0 | CHO | 264.08452 | BB | - |
| 263.113629 | 20 | 11 | 7 | 0 | 0 | 0 | CHO | 264.120905 | BB | - |
| 264.108878 | 19 | 10 | 7 | 1 | 0 | 0 | CHNO | 265.116154 | SB | - |
| 265.03875 | 14 | 9 | 7 | 0 | 1 | 0 | CHOS | 266.046026 | SB | - |
| 265.056509 | 14 | 9 | 9 | 0 | 0 | 0 | CHO | 266.063785 | SB | - |
| 265.129279 | 22 | 11 | 7 | 0 | 0 | 0 | CHO | 266.136555 | BB | - |
| 265.180919 | 26 | 16 | 3 | 0 | 0 | 0 | CHO | 266.188195 | BB | 15 |
| 266.067013 | 13 | 12 | 6 | 1 | 0 | 0 | CHNO | 267.074289 | SB | - |
| 267.014644 | 8 | 11 | 8 | 0 | 0 | 0 | CHO | 268.02192 | SB | - |
| 267.0544 | 16 | 9 | 7 | 0 | 1 | 0 | CHOS | 268.061676 | SB | 3 |
| 267.087414 | 16 | 13 | 6 | 0 | 0 | 0 | CHO | 268.09469 | BB | - |
| 267.090785 | 20 | 10 | 6 | 0 | 1 | 0 | CHOS | 268.098061 | BB | 8 |
| 267.123799 | 20 | 14 | 5 | 0 | 0 | 0 | CHO | 268.131075 | BB | - |
| 267.12717 | 24 | 11 | 5 | 0 | 1 | 0 | CHOS | 268.134446 | BB | 3 |
| 267.232954 | 32 | 17 | 2 | 0 | 0 | 0 | CHO | 268.24023 | BB | 33 |
| 268.017971 | 11 | 5 | 4 | 5 | 2 | 0 | CHNOS | 269.025247 | SB | - |
| 268.130281 | 19 | 12 | 4 | 3 | 0 | 0 | CHNO | 269.137557 | BB | 2 |
| 269.045549 | 10 | 15 | 5 | 0 | 0 | 0 | CHO | 270.052825 | BB | 42 |
| 269.081934 | 14 | 16 | 4 | 0 | 0 | 0 | CHO | 270.08921 | SB | 35 |
| 269.087809 | 18 | 9 | 9 | 0 | 0 | 0 | CHO | 270.095085 | SB | 3 |
| 269.103064 | 18 | 13 | 6 | 0 | 0 | 0 | CHO | 270.11034 | BB | - |
| 269.175834 | 26 | 15 | 4 | 0 | 0 | 0 | CHO | 270.18311 | BB | 1 |
| 270.074497 | 9 | 11 | 2 | 7 | 0 | 0 | CHNO | 271.081773 | BB | - |
| 271.009559 | 8 | 10 | 9 | 0 | 0 | 0 | CHO | 272.016835 | SB | - |
| 271.01293 | 12 | 7 | 9 | 0 | 1 | 0 | CHOS | 272.020206 | SB | - |
| 271.02503 | 16 | 7 | 3 | 2 | 3 | 0 | CHNOS | 272.032306 | BB | - |
| 271.049315 | 16 | 8 | 8 | 0 | 1 | 0 | CHOS | 272.056591 | SB | 4 |
| 271.082329 | 16 | 12 | 7 | 0 | 0 | 0 | CHO | 272.089605 | BB | 3 |
| 271.118714 | 20 | 13 | 6 | 0 | 0 | 0 | CHO | 272.12599 | BB | - |
| 271.155099 | 24 | 14 | 5 | 0 | 0 | 0 | CHO | 272.162375 | BB | - |
| 271.191484 | 28 | 15 | 4 | 0 | 0 | 0 | CHO | 272.19876 | BB | 3 |
| 272.0459 | 11 | 8 | 4 | 5 | 1 | 0 | CHNOS | 273.053176 | BB | 3 |
| 272.113963 | 19 | 12 | 6 | 1 | 0 | 0 | CHNO | 273.121239 | SB | 3 |
| 273.025209 | 10 | 10 | 9 | 0 | 0 | 0 | CHO | 274.032485 | SB | - |
| 273.040464 | 10 | 14 | 6 | 0 | 0 | 0 | CHO | 274.04774 | BB | 6 |
| 273.097979 | 18 | 12 | 7 | 0 | 0 | 0 | CHO | 274.105255 | BB | - |
| 273.134364 | 22 | 13 | 6 | 0 | 0 | 0 | CHO | 274.14164 | BB | - |
| 273.170749 | 26 | 14 | 5 | 0 | 0 | 0 | CHO | 274.178025 | BB | 2 |
| 275.0231 | 12 | 10 | 7 | 0 | 1 | 0 | CHOS | 276.030376 | SB | 1 |
| 275.04423 | 16 | 7 | 9 | 0 | 1 | 0 | CHOS | 276.051506 | SB | 3 |
| 275.056114 | 12 | 14 | 6 | 0 | 0 | 0 | CHO | 276.06339 | BB | 10 |
| 275.059485 | 16 | 11 | 6 | 0 | 1 | 0 | CHOS | 276.066761 | SB | - |
| 275.077244 | 16 | 11 | 8 | 0 | 0 | 0 | CHO | 276.08452 | BB | 1 |
| 275.092499 | 16 | 15 | 5 | 0 | 0 | 0 | CHO | 276.099775 | BB | 5 |
| 275.113629 | 20 | 12 | 7 | 0 | 0 | 0 | CHO | 276.120905 | BB | - |
| 275.16864 | 28 | 14 | 3 | 0 | 1 | 0 | CHOS | 276.175916 | BB | - |
| 276.059441 | 15 | 8 | 2 | 5 | 2 | 0 | CHNOS | 277.066717 | BB | 1 |
| 276.072493 | 15 | 10 | 8 | 1 | 0 | 0 | CHNO | 277.079769 | SB | - |
| 276.108878 | 19 | 11 | 7 | 1 | 0 | 0 | CHNO | 277.116154 | SB | - |
| 277.01762 | 10 | 13 | 5 | 0 | 1 | 0 | CHOS | 278.024896 | SB | - |
| 277.023495 | 14 | 6 | 10 | 0 | 1 | 0 | CHOS | 278.030771 | SB | - |
| 277.03875 | 14 | 10 | 7 | 0 | 1 | 0 | CHOS | 278.046026 | SB | - |
| 277.05085 | 18 | 10 | 1 | 2 | 3 | 0 | CHNOS | 278.058126 | BB | 5 |
| 277.071764 | 14 | 14 | 6 | 0 | 0 | 0 | CHO | 278.07904 | BB | - |
| 277.092894 | 18 | 11 | 8 | 0 | 0 | 0 | CHO | 278.10017 | BB | 7 |
| 277.108149 | 18 | 15 | 5 | 0 | 0 | 0 | CHO | 278.115425 | BB | 6 |
| 277.180919 | 26 | 17 | 3 | 0 | 0 | 0 | CHO | 278.188195 | BB | 7 |
| 278.075091 | 17 | 8 | 2 | 5 | 2 | 0 | CHNOS | 279.082367 | BB | - |
| 278.103398 | 17 | 14 | 5 | 1 | 0 | 0 | CHNO | 279.110674 | BB | - |
| 279.014644 | 8 | 12 | 8 | 0 | 0 | 0 | CHO | 280.02192 | SB | 1 |
| 279.019351 | 8 | 10 | 4 | 4 | 1 | 0 | CHNOS | 280.026627 | SB | - |
| 279.03711 | 8 | 10 | 6 | 4 | 0 | 0 | CHNO | 280.044386 | SB | - |
| 279.048525 | 12 | 17 | 2 | 0 | 1 | 0 | CHOS | 280.055801 | SB | - |
| 279.087414 | 16 | 14 | 6 | 0 | 0 | 0 | CHO | 280.09469 | BB | - |
| 279.144929 | 24 | 12 | 7 | 0 | 0 | 0 | CHO | 280.152205 | BB | - |
| 280.049649 | 15 | 9 | 7 | 1 | 1 | 0 | CHNOS | 281.056925 | SB | - |
| 280.082663 | 15 | 13 | 6 | 1 | 0 | 0 | CHNO | 281.089939 | SB | 10 |
| 280.090741 | 19 | 8 | 2 | 5 | 2 | 0 | CHNOS | 281.098017 | BB | - |
| 281.030294 | 10 | 12 | 8 | 0 | 0 | 0 | CHO | 282.03757 | SB | - |
| 281.033665 | 14 | 9 | 8 | 0 | 1 | 0 | CHOS | 282.040941 | SB | - |
| 281.035001 | 10 | 10 | 4 | 4 | 1 | 0 | CHNOS | 282.042277 | SB | - |
| 281.07005 | 18 | 10 | 7 | 0 | 1 | 0 | CHOS | 282.077326 | SB | 3 |
| 281.103064 | 18 | 14 | 6 | 0 | 0 | 0 | CHO | 282.11034 | BB | - |
| 281.139449 | 22 | 15 | 5 | 0 | 0 | 0 | CHO | 282.146725 | BB | 6 |
| 282.025543 | 9 | 11 | 8 | 1 | 0 | 0 | CHNO | 283.032819 | SB | - |
| 282.033621 | 13 | 6 | 4 | 5 | 2 | 0 | CHNOS | 283.040897 | SB | - |
| 282.061928 | 13 | 12 | 7 | 1 | 0 | 0 | CHNO | 283.069204 | SB | - |
| 282.106391 | 21 | 8 | 2 | 5 | 2 | 0 | CHNOS | 283.113667 | BB | - |
| 283.049315 | 16 | 9 | 8 | 0 | 1 | 0 | CHOS | 284.056591 | SB | 3 |
| 283.067074 | 16 | 9 | 10 | 0 | 0 | 0 | CHO | 284.07435 | SB | 2 |
| 283.082329 | 16 | 13 | 7 | 0 | 0 | 0 | CHO | 284.089605 | BB | 3 |
| 283.0857 | 20 | 10 | 7 | 0 | 1 | 0 | CHOS | 284.092976 | SB | 2 |
| 283.152595 | 24 | 19 | 0 | 0 | 1 | 0 | CHOS | 284.159871 | BB | - |
| 283.155099 | 24 | 15 | 5 | 0 | 0 | 0 | CHO | 284.162375 | BB | - |
| 284.113963 | 19 | 13 | 6 | 1 | 0 | 0 | CHNO | 285.121239 | SB | - |
| 285.025209 | 10 | 11 | 9 | 0 | 0 | 0 | CHO | 286.032485 | SB | - |
| 285.040464 | 10 | 15 | 6 | 0 | 0 | 0 | CHO | 286.04774 | BB | 35 |
| 285.055935 | 18 | 12 | 0 | 2 | 3 | 0 | CHNOS | 286.063211 | SB | - |
| 285.097979 | 18 | 13 | 7 | 0 | 0 | 0 | CHO | 286.105255 | BB | 4 |
| 285.119109 | 22 | 10 | 9 | 0 | 0 | 0 | CHO | 286.126385 | SB | - |
| 285.243519 | 34 | 17 | 3 | 0 | 0 | 0 | CHO | 286.250795 | BB | 7 |
| 286.076805 | 13 | 13 | 1 | 5 | 1 | 0 | CHNOS | 287.084081 | BB | - |
| 287.004474 | 8 | 10 | 10 | 0 | 0 | 0 | CHO | 288.01175 | SB | - |
| 287.019945 | 16 | 7 | 4 | 2 | 3 | 0 | CHNOS | 288.027221 | BB | - |
| 287.04423 | 16 | 8 | 9 | 0 | 1 | 0 | CHOS | 288.051506 | SB | 5 |
| 287.056114 | 12 | 15 | 6 | 0 | 0 | 0 | CHO | 288.06339 | BB | 33 |
| 287.077244 | 16 | 12 | 8 | 0 | 0 | 0 | CHO | 288.08452 | BB | - |
| 287.113629 | 20 | 13 | 7 | 0 | 0 | 0 | CHO | 288.120905 | BB | - |
| 288.080571 | 19 | 6 | 4 | 5 | 2 | 0 | CHNOS | 289.087847 | BB | - |
| 288.98711 | 10 | 6 | 11 | 0 | 1 | 0 | CHOS | 289.994386 | SB | - |
| 288.99921 | 14 | 6 | 5 | 2 | 3 | 0 | CHNOS | 290.006486 | BB | - |
| 289.023495 | 14 | 7 | 10 | 0 | 1 | 0 | CHOS | 290.030771 | SB | - |
| 289.05085 | 18 | 11 | 1 | 2 | 3 | 0 | CHNOS | 290.058126 | BB | - |
| 289.165664 | 26 | 14 | 6 | 0 | 0 | 0 | CHO | 290.17294 | BB | - |
| 290.07172 | 13 | 12 | 2 | 5 | 1 | 0 | CHNOS | 291.078996 | BB | 5 |
| 290.088143 | 17 | 11 | 8 | 1 | 0 | 0 | CHNO | 291.095419 | SB | 5 |
| 291.018015 | 12 | 10 | 8 | 0 | 1 | 0 | CHOS | 292.025291 | SB | - |
| 291.091652 | 24 | 13 | 1 | 0 | 3 | 0 | CHOS | 292.098928 | SB | 3 |
| 291.108544 | 20 | 12 | 8 | 0 | 0 | 0 | CHO | 292.11582 | BB | - |
| 291.144929 | 24 | 13 | 7 | 0 | 0 | 0 | CHO | 292.152205 | BB | 2 |
| 292.090741 | 19 | 9 | 2 | 5 | 2 | 0 | CHNOS | 293.098017 | BB | - |
| 292.994125 | 14 | 5 | 6 | 2 | 3 | 0 | CHNOS | 294.001401 | SB | - |
| 292.99728 | 10 | 9 | 9 | 0 | 1 | 0 | CHOS | 294.004556 | SB | - |
| 293.01841 | 14 | 6 | 11 | 0 | 1 | 0 | CHOS | 294.025686 | SB | - |
| 293.030294 | 10 | 13 | 8 | 0 | 0 | 0 | CHO | 294.03757 | SB | 3 |
| 293.033665 | 14 | 10 | 8 | 0 | 1 | 0 | CHOS | 294.040941 | SB | 1 |
| 293.103064 | 18 | 15 | 6 | 0 | 0 | 0 | CHO | 294.11034 | BB | 1 |
| 293.160579 | 26 | 13 | 7 | 0 | 0 | 0 | CHO | 294.167855 | BB | - |
| 293.175834 | 26 | 17 | 4 | 0 | 0 | 0 | CHO | 294.18311 | BB | 5 |
| 293.196964 | 30 | 14 | 6 | 0 | 0 | 0 | CHO | 294.20424 | BB | - |
| 293.997236 | 9 | 6 | 5 | 5 | 2 | 0 | CHNOS | 295.004512 | SB | - |
| 294.019884 | 13 | 12 | 0 | 3 | 3 | 0 | CHNOS | 295.02716 | SB | - |
| 294.025543 | 9 | 12 | 8 | 1 | 0 | 0 | CHNO | 295.032819 | SB | - |
| 294.033621 | 13 | 7 | 4 | 5 | 2 | 0 | CHNOS | 295.040897 | SB | 3 |
| 294.065299 | 17 | 10 | 7 | 1 | 1 | 0 | CHNOS | 295.072575 | BB | - |
| 294.074497 | 9 | 13 | 2 | 7 | 0 | 0 | CHNO | 295.081773 | BB | - |
| 294.106391 | 21 | 9 | 2 | 5 | 2 | 0 | CHNOS | 295.113667 | BB | 1 |
| 295.009559 | 8 | 12 | 9 | 0 | 0 | 0 | CHO | 296.016835 | SB | - |
| 295.01293 | 12 | 9 | 9 | 0 | 1 | 0 | CHOS | 296.020206 | SB | - |
| 295.036914 | 12 | 16 | 0 | 2 | 2 | 0 | CHNOS | 296.04419 | SB | - |
| 295.049315 | 16 | 10 | 8 | 0 | 1 | 0 | CHOS | 296.056591 | SB | - |
| 295.067074 | 16 | 10 | 10 | 0 | 0 | 0 | CHO | 296.07435 | SB | - |
| 295.082329 | 16 | 14 | 7 | 0 | 0 | 0 | CHO | 296.089605 | BB | 4 |
| 295.118714 | 20 | 15 | 6 | 0 | 0 | 0 | CHO | 296.12599 | BB | 6 |
| 295.122085 | 24 | 12 | 6 | 0 | 1 | 0 | CHOS | 296.129361 | BB | 2 |
| 295.139844 | 24 | 12 | 8 | 0 | 0 | 0 | CHO | 296.14712 | SB | - |
| 295.15847 | 28 | 13 | 5 | 0 | 1 | 0 | CHOS | 296.165746 | BB | 2 |
| 296.012886 | 11 | 6 | 5 | 5 | 2 | 0 | CHNOS | 297.020162 | SB | - |
| 296.161581 | 23 | 14 | 4 | 3 | 0 | 0 | CHNO | 297.168857 | BB | 2 |
| 297.016179 | 10 | 15 | 1 | 2 | 2 | 0 | CHNOS | 298.023455 | SB | - |
| 297.025209 | 10 | 12 | 9 | 0 | 0 | 0 | CHO | 298.032485 | SB | 3 |
| 297.02858 | 14 | 9 | 9 | 0 | 1 | 0 | CHOS | 298.035856 | SB | 3 |
| 297.047206 | 18 | 10 | 6 | 0 | 2 | 0 | CHOS | 298.054482 | BB | - |
| 297.061594 | 14 | 13 | 8 | 0 | 0 | 0 | CHO | 298.06887 | SB | 2 |
| 297.064965 | 18 | 10 | 8 | 0 | 1 | 0 | CHOS | 298.072241 | SB | - |
| 297.097979 | 18 | 14 | 7 | 0 | 0 | 0 | CHO | 298.105255 | BB | 4 |
| 297.119109 | 22 | 11 | 9 | 0 | 0 | 0 | CHO | 298.126385 | SB | 1 |
| 297.243519 | 34 | 18 | 3 | 0 | 0 | 0 | CHO | 298.250795 | BB | 48 |
| 298.028536 | 13 | 6 | 5 | 5 | 2 | 0 | CHNOS | 299.035812 | SB | - |
| 298.069412 | 9 | 12 | 3 | 7 | 0 | 0 | CHNO | 299.076688 | BB | - |
| 299.004474 | 8 | 11 | 10 | 0 | 0 | 0 | CHO | 300.01175 | SB | - |
| 299.019945 | 16 | 8 | 4 | 2 | 3 | 0 | CHNOS | 300.027221 | BB | 5 |
| 299.040859 | 12 | 12 | 9 | 0 | 0 | 0 | CHO | 300.048135 | SB | - |
| 299.056114 | 12 | 16 | 6 | 0 | 0 | 0 | CHO | 300.06339 | BB | 77 |
| 299.089344 | 20 | 13 | 2 | 2 | 2 | 0 | CHNOS | 300.09662 | SB | - |
| 299.092499 | 16 | 17 | 5 | 0 | 0 | 0 | CHO | 300.099775 | BB | 55 |
| 299.113629 | 20 | 14 | 7 | 0 | 0 | 0 | CHO | 300.120905 | BB | 1 |
| 299.134759 | 24 | 11 | 9 | 0 | 0 | 0 | CHO | 300.142035 | SB | - |
| 299.186399 | 28 | 16 | 5 | 0 | 0 | 0 | CHO | 300.193675 | BB | 1 |
| 299.259169 | 36 | 18 | 3 | 0 | 0 | 0 | CHO | 300.266445 | BB | 29 |
| 300.044186 | 15 | 6 | 5 | 5 | 2 | 0 | CHNOS | 301.051462 | SB | - |
| 300.048677 | 7 | 11 | 4 | 7 | 0 | 0 | CHNO | 301.055953 | SB | - |
| 301.020124 | 10 | 11 | 10 | 0 | 0 | 0 | CHO | 302.0274 | SB | - |
| 301.023495 | 14 | 8 | 10 | 0 | 1 | 0 | CHOS | 302.030771 | SB | - |
| 301.03875 | 14 | 12 | 7 | 0 | 1 | 0 | CHOS | 302.046026 | SB | 3 |
| 301.071764 | 14 | 16 | 6 | 0 | 0 | 0 | CHO | 302.07904 | BB | 37 |
| 301.104127 | 18 | 12 | 7 | 2 | 0 | 0 | CHNO | 302.111403 | SB | 5 |
| 301.140512 | 22 | 13 | 6 | 2 | 0 | 0 | CHNO | 302.147788 | SB | - |
| 301.165664 | 26 | 15 | 6 | 0 | 0 | 0 | CHO | 302.17294 | BB | 1 |
| 301.217304 | 30 | 20 | 2 | 0 | 0 | 0 | CHO | 302.22458 | BB | 50 |
| 302.051758 | 13 | 11 | 9 | 1 | 0 | 0 | CHNO | 303.059034 | SB | - |
| 302.07172 | 13 | 13 | 2 | 5 | 1 | 0 | CHNOS | 303.078996 | BB | - |
| 302.075091 | 17 | 10 | 2 | 5 | 2 | 0 | CHNOS | 303.082367 | BB | 37 |
| 303.00276 | 12 | 7 | 11 | 0 | 1 | 0 | CHOS | 304.010036 | SB | - |
| 303.018015 | 12 | 11 | 8 | 0 | 1 | 0 | CHOS | 304.025291 | SB | 1 |
| 303.035774 | 12 | 11 | 10 | 0 | 0 | 0 | CHO | 304.04305 | SB | 2 |
| 303.039145 | 16 | 8 | 10 | 0 | 1 | 0 | CHOS | 304.046421 | SB | 3 |
| 303.051029 | 12 | 15 | 7 | 0 | 0 | 0 | CHO | 304.058305 | BB | 8 |
| 303.0544 | 16 | 12 | 7 | 0 | 1 | 0 | CHOS | 304.061676 | SB | 3 |
| 303.087414 | 16 | 16 | 6 | 0 | 0 | 0 | CHO | 304.09469 | BB | 12 |
| 303.144929 | 24 | 14 | 7 | 0 | 0 | 0 | CHO | 304.152205 | BB | - |
| 303.19994 | 32 | 16 | 3 | 0 | 1 | 0 | CHOS | 304.207216 | BB | - |
| 304.090741 | 19 | 10 | 2 | 5 | 2 | 0 | CHNOS | 305.098017 | BB | 12 |
| 305.033665 | 14 | 11 | 8 | 0 | 1 | 0 | CHOS | 306.040941 | SB | 3 |
| 305.054795 | 18 | 8 | 10 | 0 | 1 | 0 | CHOS | 306.062071 | SB | - |
| 305.066679 | 14 | 15 | 7 | 0 | 0 | 0 | CHO | 306.073955 | BB | 16 |
| 305.07005 | 18 | 12 | 7 | 0 | 1 | 0 | CHOS | 306.077326 | SB | 4 |
| 305.087809 | 18 | 12 | 9 | 0 | 0 | 0 | CHO | 306.095085 | BB | - |
| 305.103064 | 18 | 16 | 6 | 0 | 0 | 0 | CHO | 306.11034 | BB | 1 |
| 305.124194 | 22 | 13 | 8 | 0 | 0 | 0 | CHO | 306.13147 | BB | - |
| 305.175834 | 26 | 18 | 4 | 0 | 0 | 0 | CHO | 306.18311 | BB | 2 |
| 305.997236 | 9 | 7 | 5 | 5 | 2 | 0 | CHNOS | 307.004512 | SB | - |
| 306.033621 | 13 | 8 | 4 | 5 | 2 | 0 | CHNOS | 307.040897 | SB | - |
| 306.070006 | 17 | 9 | 3 | 5 | 2 | 0 | CHNOS | 307.077282 | BB | 12 |
| 306.074497 | 9 | 14 | 2 | 7 | 0 | 0 | CHNO | 307.081773 | BB | - |
| 306.083058 | 17 | 11 | 9 | 1 | 0 | 0 | CHNO | 307.090334 | SB | - |
| 306.098313 | 17 | 15 | 6 | 1 | 0 | 0 | CHNO | 307.105589 | SB | - |
| 307.009559 | 8 | 13 | 9 | 0 | 0 | 0 | CHO | 308.016835 | SB | - |
| 307.049315 | 16 | 11 | 8 | 0 | 1 | 0 | CHOS | 308.056591 | SB | 1 |
| 307.082329 | 16 | 15 | 7 | 0 | 0 | 0 | CHO | 308.089605 | BB | 1 |
| 307.103459 | 20 | 12 | 9 | 0 | 0 | 0 | CHO | 308.110735 | BB | - |
| 307.139844 | 24 | 13 | 8 | 0 | 0 | 0 | CHO | 308.14712 | BB | - |
| 307.176229 | 28 | 14 | 7 | 0 | 0 | 0 | CHO | 308.183505 | BB | - |
| 308.012886 | 11 | 7 | 5 | 5 | 2 | 0 | CHNOS | 309.020162 | SB | - |
| 308.041193 | 11 | 13 | 8 | 1 | 0 | 0 | CHNO | 309.048469 | SB | - |
| 308.077578 | 15 | 14 | 7 | 1 | 0 | 0 | CHNO | 309.084854 | SB | 26 |
| 308.085656 | 19 | 9 | 3 | 5 | 2 | 0 | CHNOS | 309.092932 | BB | 2 |
| 308.090147 | 11 | 14 | 2 | 7 | 0 | 0 | CHNO | 309.097423 | BB | - |
| 308.098708 | 19 | 11 | 9 | 1 | 0 | 0 | CHNO | 309.105984 | SB | 4 |
| 308.135093 | 23 | 12 | 8 | 1 | 0 | 0 | CHNO | 309.142369 | SB | - |
| 309.025209 | 10 | 13 | 9 | 0 | 0 | 0 | CHO | 310.032485 | SB | - |
| 309.061594 | 14 | 14 | 8 | 0 | 0 | 0 | CHO | 310.06887 | SB | - |
| 309.082724 | 18 | 11 | 10 | 0 | 0 | 0 | CHO | 310.09 | SB | - |
| 309.097979 | 18 | 15 | 7 | 0 | 0 | 0 | CHO | 310.105255 | BB | 5 |
| 309.102217 | 26 | 13 | 2 | 0 | 3 | 0 | CHOS | 310.109493 | SB | 4 |
| 309.137735 | 26 | 13 | 6 | 0 | 1 | 0 | CHOS | 310.145011 | BB | - |
| 310.028536 | 13 | 7 | 5 | 5 | 2 | 0 | CHNOS | 311.035812 | SB | - |
| 310.064921 | 17 | 8 | 4 | 5 | 2 | 0 | CHNOS | 311.072197 | SB | - |
| 310.069412 | 9 | 13 | 3 | 7 | 0 | 0 | CHNO | 311.076688 | BB | - |
| 310.101306 | 21 | 9 | 3 | 5 | 2 | 0 | CHNOS | 311.108582 | BB | 4 |
| 310.114358 | 21 | 11 | 9 | 1 | 0 | 0 | CHNO | 311.121634 | SB | - |
| 311.004474 | 8 | 12 | 10 | 0 | 0 | 0 | CHO | 312.01175 | SB | - |
| 311.061989 | 16 | 10 | 11 | 0 | 0 | 0 | CHO | 312.069265 | SB | - |
| 311.113629 | 20 | 15 | 7 | 0 | 0 | 0 | CHO | 312.120905 | BB | 5 |
| 311.161247 | 24 | 15 | 5 | 2 | 0 | 0 | CHNO | 312.168523 | SB | - |
| 312.030449 | 15 | 12 | 1 | 3 | 3 | 0 | CHNOS | 313.037725 | SB | - |
| 312.116956 | 23 | 9 | 3 | 5 | 2 | 0 | CHNOS | 313.124232 | BB | 4 |
| 313.056509 | 14 | 13 | 9 | 0 | 0 | 0 | CHO | 314.063785 | SB | 2 |
| 313.071764 | 14 | 17 | 6 | 0 | 0 | 0 | CHO | 314.07904 | BB | 74 |
| 313.077639 | 18 | 10 | 11 | 0 | 0 | 0 | CHO | 314.084915 | SB | - |
| 313.078506 | 22 | 11 | 6 | 0 | 2 | 0 | CHOS | 314.085782 | BB | - |
| 313.129279 | 22 | 15 | 7 | 0 | 0 | 0 | CHO | 314.136555 | BB | - |
| 313.165664 | 26 | 16 | 6 | 0 | 0 | 0 | CHO | 314.17294 | BB | - |
| 314.017576 | 9 | 13 | 1 | 5 | 2 | 0 | CHNOS | 315.024852 | SB | - |
| 314.046099 | 17 | 12 | 1 | 3 | 3 | 0 | CHNOS | 315.053375 | SB | - |
| 314.059836 | 17 | 7 | 5 | 5 | 2 | 0 | CHNOS | 315.067112 | SB | 3 |
| 314.078246 | 13 | 15 | 5 | 3 | 0 | 0 | CHNO | 315.085522 | BB | - |
| 315.035774 | 12 | 12 | 10 | 0 | 0 | 0 | CHO | 316.04305 | SB | - |
| 315.051029 | 12 | 16 | 7 | 0 | 0 | 0 | CHO | 316.058305 | BB | 46 |
| 315.093289 | 20 | 10 | 11 | 0 | 0 | 0 | CHO | 316.100565 | SB | - |
| 315.108544 | 20 | 14 | 8 | 0 | 0 | 0 | CHO | 316.11582 | BB | - |
| 315.129674 | 24 | 11 | 10 | 0 | 0 | 0 | CHO | 316.13695 | SB | - |
| 315.144929 | 24 | 15 | 7 | 0 | 0 | 0 | CHO | 316.152205 | BB | - |
| 316.017971 | 11 | 9 | 4 | 5 | 2 | 0 | CHNOS | 317.025247 | BB | - |
| 316.069611 | 15 | 14 | 0 | 5 | 2 | 0 | CHNOS | 317.076887 | BB | - |
| 316.08737 | 15 | 14 | 2 | 5 | 1 | 0 | CHNOS | 317.094646 | BB | - |
| 316.111871 | 23 | 8 | 4 | 5 | 2 | 0 | CHNOS | 317.119147 | BB | - |
| 316.212948 | 31 | 16 | 5 | 1 | 0 | 0 | CHNO | 317.220224 | SB | - |
| 317.01841 | 14 | 8 | 11 | 0 | 1 | 0 | CHOS | 318.025686 | SB | - |
| 317.033665 | 14 | 12 | 8 | 0 | 1 | 0 | CHOS | 318.040941 | SB | 1 |
| 317.054795 | 18 | 9 | 10 | 0 | 1 | 0 | CHOS | 318.062071 | SB | - |
| 317.066679 | 14 | 16 | 7 | 0 | 0 | 0 | CHO | 318.073955 | BB | 10 |
| 317.07005 | 18 | 13 | 7 | 0 | 1 | 0 | CHOS | 318.077326 | SB | 1 |
| 317.103064 | 18 | 17 | 6 | 0 | 0 | 0 | CHO | 318.11034 | BB | 7 |
| 317.196964 | 30 | 16 | 6 | 0 | 0 | 0 | CHO | 318.20424 | BB | 3 |
| 317.233349 | 34 | 17 | 5 | 0 | 0 | 0 | CHO | 318.240625 | BB | - |
| 318.03025 | 9 | 12 | 4 | 5 | 1 | 0 | CHNOS | 319.037526 | SB | 3 |
| 318.050044 | 17 | 8 | 10 | 1 | 1 | 0 | CHNOS | 319.05732 | SB | 2 |
| 318.070006 | 17 | 10 | 3 | 5 | 2 | 0 | CHNOS | 319.077282 | BB | 9 |
| 318.083058 | 17 | 12 | 9 | 1 | 0 | 0 | CHNO | 319.090334 | SB | - |
| 318.085261 | 17 | 14 | 0 | 5 | 2 | 0 | CHNOS | 319.092537 | BB | - |
| 318.106391 | 21 | 11 | 2 | 5 | 2 | 0 | CHNOS | 319.113667 | BB | 7 |
| 319.009775 | 16 | 7 | 6 | 2 | 3 | 0 | CHNOS | 320.017051 | SB | - |
| 319.01293 | 12 | 11 | 9 | 0 | 1 | 0 | CHOS | 320.020206 | SB | - |
| 319.082329 | 16 | 16 | 7 | 0 | 0 | 0 | CHO | 320.089605 | BB | 14 |
| 319.0857 | 20 | 13 | 7 | 0 | 1 | 0 | CHOS | 320.092976 | SB | - |
| 319.103459 | 20 | 13 | 9 | 0 | 0 | 0 | CHO | 320.110735 | BB | - |
| 319.139844 | 24 | 14 | 8 | 0 | 0 | 0 | CHO | 320.14712 | BB | 2 |
| 319.212614 | 32 | 16 | 6 | 0 | 0 | 0 | CHO | 320.21989 | BB | - |
| 319.23124 | 36 | 17 | 3 | 0 | 1 | 0 | CHOS | 320.238516 | BB | - |
| 320.085656 | 19 | 10 | 3 | 5 | 2 | 0 | CHNOS | 321.092932 | BB | 8 |
| 320.098708 | 19 | 12 | 9 | 1 | 0 | 0 | CHNO | 321.105984 | SB | - |
| 320.122041 | 23 | 11 | 2 | 5 | 2 | 0 | CHNOS | 321.129317 | BB | - |
| 321.025209 | 10 | 14 | 9 | 0 | 0 | 0 | CHO | 322.032485 | SB | 1 |
| 321.02858 | 14 | 11 | 9 | 0 | 1 | 0 | CHOS | 322.035856 | SB | - |
| 321.04971 | 18 | 8 | 11 | 0 | 1 | 0 | CHOS | 322.056986 | SB | - |
| 321.097979 | 18 | 16 | 7 | 0 | 0 | 0 | CHO | 322.105255 | BB | - |
| 321.119109 | 22 | 13 | 9 | 0 | 0 | 0 | CHO | 322.126385 | BB | - |
| 321.134364 | 22 | 17 | 6 | 0 | 0 | 0 | CHO | 322.14164 | BB | 2 |
| 321.191879 | 30 | 15 | 7 | 0 | 0 | 0 | CHO | 322.199155 | BB | - |
| 321.207134 | 30 | 19 | 4 | 0 | 0 | 0 | CHO | 322.21441 | BB | 3 |
| 321.243519 | 34 | 20 | 3 | 0 | 0 | 0 | CHO | 322.250795 | BB | 12 |
| 322.028536 | 13 | 8 | 5 | 5 | 2 | 0 | CHNOS | 323.035812 | SB | 3 |
| 322.056843 | 13 | 14 | 8 | 1 | 0 | 0 | CHNO | 323.064119 | SB | - |
| 322.069412 | 9 | 14 | 3 | 7 | 0 | 0 | CHNO | 323.076688 | BB | - |
| 322.093228 | 17 | 15 | 7 | 1 | 0 | 0 | CHNO | 323.100504 | SB | - |
| 322.101306 | 21 | 10 | 3 | 5 | 2 | 0 | CHNOS | 323.108582 | BB | - |
| 322.114358 | 21 | 12 | 9 | 1 | 0 | 0 | CHNO | 323.121634 | SB | - |
| 323.004474 | 8 | 13 | 10 | 0 | 0 | 0 | CHO | 324.01175 | SB | - |
| 323.022449 | 16 | 6 | 9 | 2 | 2 | 0 | CHNOS | 324.029725 | SB | - |
| 323.040859 | 12 | 14 | 9 | 0 | 0 | 0 | CHO | 324.048135 | SB | - |
| 323.055463 | 16 | 10 | 8 | 2 | 1 | 0 | CHNOS | 324.062739 | SB | - |
| 323.113629 | 20 | 16 | 7 | 0 | 0 | 0 | CHO | 324.120905 | BB | - |
| 323.153385 | 28 | 14 | 6 | 0 | 1 | 0 | CHOS | 324.160661 | BB | - |
| 323.207529 | 32 | 15 | 7 | 0 | 0 | 0 | CHO | 324.214805 | BB | - |
| 323.259169 | 36 | 20 | 3 | 0 | 0 | 0 | CHO | 324.266445 | BB | 5 |
| 324.007801 | 11 | 7 | 6 | 5 | 2 | 0 | CHNOS | 325.015077 | SB | 1 |
| 324.044186 | 15 | 8 | 5 | 5 | 2 | 0 | CHNOS | 325.051462 | SB | - |
| 324.072493 | 15 | 14 | 8 | 1 | 0 | 0 | CHNO | 325.079769 | SB | 15 |
| 324.085062 | 11 | 14 | 3 | 7 | 0 | 0 | CHNO | 325.092338 | BB | - |
| 324.093623 | 19 | 11 | 10 | 1 | 0 | 0 | CHNO | 325.100899 | SB | 3 |
| 324.116956 | 23 | 10 | 3 | 5 | 2 | 0 | CHNOS | 325.124232 | BB | - |
| 324.99921 | 14 | 9 | 5 | 2 | 3 | 0 | CHNOS | 326.006486 | BB | - |
| 325.020124 | 10 | 13 | 10 | 0 | 0 | 0 | CHO | 326.0274 | SB | - |
| 325.047479 | 14 | 17 | 1 | 2 | 2 | 0 | CHNOS | 326.054755 | SB | - |
| 325.056509 | 14 | 14 | 9 | 0 | 0 | 0 | CHO | 326.063785 | SB | 1 |
| 325.077639 | 18 | 11 | 11 | 0 | 0 | 0 | CHO | 326.084915 | SB | - |
| 325.104994 | 22 | 15 | 2 | 2 | 2 | 0 | CHNOS | 326.11227 | SB | - |
| 325.129279 | 22 | 16 | 7 | 0 | 0 | 0 | CHO | 326.136555 | BB | 1 |
| 325.202049 | 30 | 18 | 5 | 0 | 0 | 0 | CHO | 326.209325 | BB | 7 |
| 325.274819 | 38 | 20 | 3 | 0 | 0 | 0 | CHO | 326.282095 | BB | 8 |
| 325.311204 | 42 | 21 | 2 | 0 | 0 | 0 | CHO | 326.31848 | BB | 8 |
| 326.023451 | 13 | 7 | 6 | 5 | 2 | 0 | CHNOS | 327.030727 | SB | - |
| 326.051758 | 13 | 13 | 9 | 1 | 0 | 0 | CHNO | 327.059034 | SB | - |
| 326.059836 | 17 | 8 | 5 | 5 | 2 | 0 | CHNOS | 327.067112 | SB | 3 |
| 326.07172 | 13 | 15 | 2 | 5 | 1 | 0 | CHNOS | 327.078996 | BB | - |
| 326.109273 | 21 | 11 | 10 | 1 | 0 | 0 | CHNO | 327.116549 | SB | - |
| 326.999389 | 8 | 12 | 11 | 0 | 0 | 0 | CHO | 328.006665 | SB | - |
| 327.035774 | 12 | 13 | 10 | 0 | 0 | 0 | CHO | 328.04305 | SB | - |
| 327.063129 | 16 | 17 | 1 | 2 | 2 | 0 | CHNOS | 328.070405 | SB | - |
| 327.117958 | 20 | 11 | 0 | 8 | 2 | 0 | CHNOS | 328.125234 | SB | - |
| 327.120644 | 24 | 15 | 2 | 2 | 2 | 0 | CHNOS | 328.12792 | SB | - |
| 327.144929 | 24 | 16 | 7 | 0 | 0 | 0 | CHO | 328.152205 | BB | 4 |
| 327.181314 | 28 | 17 | 6 | 0 | 0 | 0 | CHO | 328.18859 | BB | - |
| 327.217699 | 32 | 18 | 5 | 0 | 0 | 0 | CHO | 328.224975 | BB | 7 |
| 328.033226 | 11 | 14 | 1 | 5 | 2 | 0 | CHNOS | 329.040502 | SB | - |
| 328.039101 | 15 | 7 | 6 | 5 | 2 | 0 | CHNOS | 329.046377 | SB | - |
| 328.069611 | 15 | 15 | 0 | 5 | 2 | 0 | CHNOS | 329.076887 | BB | - |
| 329.015039 | 10 | 12 | 11 | 0 | 0 | 0 | CHO | 330.022315 | SB | - |
| 329.051424 | 14 | 13 | 10 | 0 | 0 | 0 | CHO | 330.0587 | SB | - |
| 329.097223 | 18 | 10 | 1 | 8 | 2 | 0 | CHNOS | 330.104499 | SB | - |
| 329.103064 | 18 | 18 | 6 | 0 | 0 | 0 | CHO | 330.11034 | BB | 34 |
| 329.14282 | 26 | 16 | 5 | 0 | 1 | 0 | CHOS | 330.150096 | BB | 5 |
| 329.160579 | 26 | 16 | 7 | 0 | 0 | 0 | CHO | 330.167855 | BB | 11 |
| 329.196964 | 30 | 17 | 6 | 0 | 0 | 0 | CHO | 330.20424 | BB | - |
| 329.233349 | 34 | 18 | 5 | 0 | 0 | 0 | CHO | 330.240625 | BB | 13 |
| 329.269734 | 38 | 19 | 4 | 0 | 0 | 0 | CHO | 330.27701 | BB | 5 |
| 330.054751 | 17 | 7 | 6 | 5 | 2 | 0 | CHNOS | 331.062027 | SB | - |
| 330.083058 | 17 | 13 | 9 | 1 | 0 | 0 | CHNO | 331.090334 | SB | - |
| 330.085261 | 17 | 15 | 0 | 5 | 2 | 0 | CHNOS | 331.092537 | BB | - |
| 331.01293 | 12 | 12 | 9 | 0 | 1 | 0 | CHOS | 332.020206 | SB | - |
| 331.030689 | 12 | 12 | 11 | 0 | 0 | 0 | CHO | 332.037965 | SB | - |
| 331.03406 | 16 | 9 | 11 | 0 | 1 | 0 | CHOS | 332.041336 | SB | - |
| 331.092911 | 20 | 8 | 8 | 4 | 1 | 0 | CHNOS | 332.100187 | BB | 3 |
| 331.114692 | 20 | 13 | 8 | 2 | 0 | 0 | CHNO | 332.121968 | SB | - |
| 331.124589 | 24 | 11 | 11 | 0 | 0 | 0 | CHO | 332.131865 | SB | - |
| 332.082285 | 15 | 14 | 3 | 5 | 1 | 0 | CHNOS | 333.089561 | BB | - |
| 332.085656 | 19 | 11 | 3 | 5 | 2 | 0 | CHNOS | 333.092932 | BB | - |
| 333.064965 | 18 | 13 | 8 | 0 | 1 | 0 | CHOS | 334.072241 | SB | - |
| 333.097979 | 18 | 17 | 7 | 0 | 0 | 0 | CHO | 334.105255 | BB | 4 |
| 333.10135 | 22 | 14 | 7 | 0 | 1 | 0 | CHOS | 334.108626 | SB | - |
| 333.119109 | 22 | 14 | 9 | 0 | 0 | 0 | CHO | 334.126385 | BB | - |
| 333.24689 | 38 | 18 | 3 | 0 | 1 | 0 | CHOS | 334.254166 | BB | - |
| 334.056843 | 13 | 15 | 8 | 1 | 0 | 0 | CHNO | 335.064119 | SB | - |
| 334.068076 | 13 | 14 | 7 | 3 | 0 | 0 | CHNO | 335.075352 | SB | - |
| 334.097935 | 17 | 14 | 3 | 5 | 1 | 0 | CHNOS | 335.105211 | BB | - |
| 334.104461 | 17 | 15 | 6 | 3 | 0 | 0 | CHNO | 335.111737 | SB | - |
| 334.114358 | 21 | 13 | 9 | 1 | 0 | 0 | CHNO | 335.121634 | SB | - |
| 334.223513 | 33 | 16 | 6 | 1 | 0 | 0 | CHNO | 335.230789 | SB | - |
| 335.00469 | 16 | 7 | 7 | 2 | 3 | 0 | CHNOS | 336.011966 | SB | - |
| 335.022449 | 16 | 7 | 9 | 2 | 2 | 0 | CHNOS | 336.029725 | SB | 2 |
| 335.040859 | 12 | 15 | 9 | 0 | 0 | 0 | CHO | 336.048135 | SB | 1 |
| 335.080615 | 20 | 13 | 8 | 0 | 1 | 0 | CHOS | 336.087891 | SB | 4 |
| 335.113629 | 20 | 17 | 7 | 0 | 0 | 0 | CHO | 336.120905 | BB | - |
| 335.134759 | 24 | 14 | 9 | 0 | 0 | 0 | CHO | 336.142035 | BB | - |
| 335.171144 | 28 | 15 | 8 | 0 | 0 | 0 | CHO | 336.17842 | BB | - |
| 335.207529 | 32 | 16 | 7 | 0 | 0 | 0 | CHO | 336.214805 | BB | - |
| 336.007801 | 11 | 8 | 6 | 5 | 2 | 0 | CHNOS | 337.015077 | SB | - |
| 336.044186 | 15 | 9 | 5 | 5 | 2 | 0 | CHNOS | 337.051462 | SB | 1 |
| 336.072493 | 15 | 15 | 8 | 1 | 0 | 0 | CHNO | 337.079769 | SB | 58 |
| 336.085062 | 11 | 15 | 3 | 7 | 0 | 0 | CHNO | 337.092338 | BB | - |
| 336.108878 | 19 | 16 | 7 | 1 | 0 | 0 | CHNO | 337.116154 | SB | 12 |
| 336.130008 | 23 | 13 | 9 | 1 | 0 | 0 | CHNO | 337.137284 | SB | 3 |
| 337.020124 | 10 | 14 | 10 | 0 | 0 | 0 | CHO | 338.0274 | SB | - |
| 337.056509 | 14 | 15 | 9 | 0 | 0 | 0 | CHO | 338.063785 | SB | - |
| 337.071113 | 18 | 11 | 8 | 2 | 1 | 0 | CHNOS | 338.078389 | SB | - |
| 337.077639 | 18 | 12 | 11 | 0 | 0 | 0 | CHO | 338.084915 | SB | 1 |
| 337.129279 | 22 | 17 | 7 | 0 | 0 | 0 | CHO | 338.136555 | BB | 1 |
| 337.150409 | 26 | 14 | 9 | 0 | 0 | 0 | CHO | 338.157685 | BB | - |
| 338.023451 | 13 | 8 | 6 | 5 | 2 | 0 | CHNOS | 339.030727 | SB | - |
| 338.051758 | 13 | 14 | 9 | 1 | 0 | 0 | CHNO | 339.059034 | SB | - |
| 338.059836 | 17 | 9 | 5 | 5 | 2 | 0 | CHNOS | 339.067112 | SB | - |
| 338.082484 | 21 | 15 | 0 | 3 | 3 | 0 | CHNOS | 339.08976 | BB | - |
| 338.088143 | 17 | 15 | 8 | 1 | 0 | 0 | CHNO | 339.095419 | SB | 25 |
| 338.999389 | 8 | 13 | 11 | 0 | 0 | 0 | CHO | 340.006665 | SB | - |
| 339.035774 | 12 | 14 | 10 | 0 | 0 | 0 | CHO | 340.04305 | SB | - |
| 339.051029 | 12 | 18 | 7 | 0 | 0 | 0 | CHO | 340.058305 | BB | 2 |
| 339.056904 | 16 | 11 | 12 | 0 | 0 | 0 | CHO | 340.06418 | SB | - |
| 339.087414 | 16 | 19 | 6 | 0 | 0 | 0 | CHO | 340.09469 | BB | 3 |
| 339.093289 | 20 | 12 | 11 | 0 | 0 | 0 | CHO | 340.100565 | SB | 4 |
| 339.108544 | 20 | 16 | 8 | 0 | 0 | 0 | CHO | 340.11582 | BB | - |
| 339.181314 | 28 | 18 | 6 | 0 | 0 | 0 | CHO | 340.18859 | BB | - |
| 339.326854 | 44 | 22 | 2 | 0 | 0 | 0 | CHO | 340.33413 | BB | 7 |
| 340.039101 | 15 | 8 | 6 | 5 | 2 | 0 | CHNOS | 341.046377 | SB | - |
| 340.046278 | 11 | 17 | 7 | 1 | 0 | 0 | CHNO | 341.053554 | BB | 2 |
| 340.111871 | 23 | 10 | 4 | 5 | 2 | 0 | CHNOS | 341.119147 | BB | - |
| 341.015039 | 10 | 13 | 11 | 0 | 0 | 0 | CHO | 342.022315 | SB | - |
| 341.051424 | 14 | 14 | 10 | 0 | 0 | 0 | CHO | 342.0587 | SB | - |
| 341.072554 | 18 | 11 | 12 | 0 | 0 | 0 | CHO | 342.07983 | SB | - |
| 341.097223 | 18 | 11 | 1 | 8 | 2 | 0 | CHNOS | 342.104499 | SB | 7 |
| 341.108939 | 22 | 12 | 11 | 0 | 0 | 0 | CHO | 342.116215 | SB | 46 |
| 341.145324 | 26 | 13 | 10 | 0 | 0 | 0 | CHO | 342.1526 | SB | - |
| 341.196964 | 30 | 18 | 6 | 0 | 0 | 0 | CHO | 342.20424 | BB | 4 |
| 342.041014 | 17 | 13 | 2 | 3 | 3 | 0 | CHNOS | 343.04829 | SB | - |
| 342.054751 | 17 | 8 | 6 | 5 | 2 | 0 | CHNOS | 343.062027 | SB | - |
| 342.119443 | 21 | 15 | 8 | 1 | 0 | 0 | CHNO | 343.126719 | SB | - |
| 342.127521 | 25 | 10 | 4 | 5 | 2 | 0 | CHNOS | 343.134797 | BB | - |
| 343.030689 | 12 | 13 | 11 | 0 | 0 | 0 | CHO | 344.037965 | SB | - |
| 343.03406 | 16 | 10 | 11 | 0 | 1 | 0 | CHOS | 344.041336 | SB | - |
| 343.061415 | 20 | 14 | 2 | 2 | 3 | 0 | CHNOS | 344.068691 | BB | - |
| 343.067074 | 16 | 14 | 10 | 0 | 0 | 0 | CHO | 344.07435 | SB | 1 |
| 343.088204 | 20 | 11 | 12 | 0 | 0 | 0 | CHO | 344.09548 | SB | - |
| 343.092911 | 20 | 9 | 8 | 4 | 1 | 0 | CHNOS | 344.100187 | BB | - |
| 343.112873 | 20 | 11 | 1 | 8 | 2 | 0 | CHNOS | 344.120149 | SB | 60 |
| 343.115559 | 24 | 15 | 3 | 2 | 2 | 0 | CHNOS | 344.122835 | SB | - |
| 343.124589 | 24 | 12 | 11 | 0 | 0 | 0 | CHO | 344.131865 | SB | 4 |
| 344.012886 | 11 | 10 | 5 | 5 | 2 | 0 | CHNOS | 345.020162 | SB | - |
| 344.049271 | 15 | 11 | 4 | 5 | 2 | 0 | CHNOS | 345.056547 | BB | - |
| 344.100911 | 19 | 16 | 0 | 5 | 2 | 0 | CHNOS | 345.108187 | BB | - |
| 345.046339 | 14 | 13 | 11 | 0 | 0 | 0 | CHO | 346.053615 | SB | - |
| 345.04971 | 18 | 10 | 11 | 0 | 1 | 0 | CHOS | 346.056986 | SB | - |
| 345.064965 | 18 | 14 | 8 | 0 | 1 | 0 | CHOS | 346.072241 | SB | - |
| 345.103854 | 22 | 11 | 12 | 0 | 0 | 0 | CHO | 346.11113 | SB | - |
| 345.155494 | 26 | 16 | 8 | 0 | 0 | 0 | CHO | 346.16277 | BB | - |
| 345.191879 | 30 | 17 | 7 | 0 | 0 | 0 | CHO | 346.199155 | BB | 2 |
| 346.025165 | 9 | 13 | 5 | 5 | 1 | 0 | CHNOS | 347.032441 | SB | - |
| 346.028536 | 13 | 10 | 5 | 5 | 2 | 0 | CHNOS | 347.035812 | SB | - |
| 346.080176 | 17 | 15 | 1 | 5 | 2 | 0 | CHNOS | 347.087452 | BB | - |
| 346.093228 | 17 | 17 | 7 | 1 | 0 | 0 | CHNO | 347.100504 | SB | - |
| 346.114358 | 21 | 14 | 9 | 1 | 0 | 0 | CHNO | 347.121634 | SB | - |
| 346.974831 | 12 | 8 | 11 | 0 | 2 | 0 | CHOS | 347.982107 | BB | - |
| 347.007845 | 12 | 12 | 10 | 0 | 1 | 0 | CHOS | 348.015121 | SB | - |
| 347.022449 | 16 | 8 | 9 | 2 | 2 | 0 | CHNOS | 348.029725 | SB | - |
| 347.04423 | 16 | 13 | 9 | 0 | 1 | 0 | CHOS | 348.051506 | SB | - |
| 347.087826 | 20 | 8 | 9 | 4 | 1 | 0 | CHNOS | 348.095102 | BB | - |
| 347.117 | 24 | 15 | 7 | 0 | 1 | 0 | CHOS | 348.124276 | SB | 2 |
| 347.134759 | 24 | 15 | 9 | 0 | 0 | 0 | CHO | 348.142035 | BB | 3 |
| 347.150014 | 24 | 19 | 6 | 0 | 0 | 0 | CHO | 348.15729 | BB | 16 |
| 347.207529 | 32 | 17 | 7 | 0 | 0 | 0 | CHO | 348.214805 | BB | - |
| 347.234702 | 32 | 13 | 1 | 8 | 1 | 0 | CHNOS | 348.241978 | BB | - |
| 347.243914 | 36 | 18 | 6 | 0 | 0 | 0 | CHO | 348.25119 | BB | 1 |
| 348.040815 | 11 | 13 | 5 | 5 | 1 | 0 | CHNOS | 349.048091 | SB | - |
| 348.072493 | 15 | 16 | 8 | 1 | 0 | 0 | CHNO | 349.079769 | SB | - |
| 349.020124 | 10 | 15 | 10 | 0 | 0 | 0 | CHO | 350.0274 | SB | - |
| 349.114024 | 22 | 14 | 10 | 0 | 0 | 0 | CHO | 350.1213 | BB | 2 |
| 349.150409 | 26 | 15 | 9 | 0 | 0 | 0 | CHO | 350.157685 | BB | - |
| 349.223179 | 34 | 17 | 7 | 0 | 0 | 0 | CHO | 350.230455 | BB | - |
| 350.023451 | 13 | 9 | 6 | 5 | 2 | 0 | CHNOS | 351.030727 | SB | - |
| 350.051758 | 13 | 15 | 9 | 1 | 0 | 0 | CHNO | 351.059034 | SB | - |
| 350.082484 | 21 | 16 | 0 | 3 | 3 | 0 | CHNOS | 351.08976 | BB | - |
| 350.088143 | 17 | 16 | 8 | 1 | 0 | 0 | CHNO | 351.095419 | SB | - |
| 350.999605 | 16 | 7 | 8 | 2 | 3 | 0 | CHNOS | 352.006881 | SB | - |
| 351.035774 | 12 | 15 | 10 | 0 | 0 | 0 | CHO | 352.04305 | SB | - |
| 351.072159 | 16 | 16 | 9 | 0 | 0 | 0 | CHO | 352.079435 | SB | 1 |
| 351.086763 | 20 | 12 | 8 | 2 | 1 | 0 | CHNOS | 352.094039 | SB | - |
| 351.108544 | 20 | 17 | 8 | 0 | 0 | 0 | CHO | 352.11582 | BB | - |
| 351.144929 | 24 | 18 | 7 | 0 | 0 | 0 | CHO | 352.152205 | BB | - |
| 351.166059 | 28 | 15 | 9 | 0 | 0 | 0 | CHO | 352.173335 | BB | - |
| 352.039101 | 15 | 9 | 6 | 5 | 2 | 0 | CHNOS | 353.046377 | SB | - |
| 352.067408 | 15 | 15 | 9 | 1 | 0 | 0 | CHNO | 353.074684 | SB | - |
| 352.079977 | 11 | 15 | 4 | 7 | 0 | 0 | CHNO | 353.087253 | BB | - |
| 352.111871 | 23 | 11 | 4 | 5 | 2 | 0 | CHNOS | 353.119147 | BB | - |
| 352.124923 | 23 | 13 | 10 | 1 | 0 | 0 | CHNO | 353.132199 | SB | - |
| 353.015039 | 10 | 14 | 11 | 0 | 0 | 0 | CHO | 354.022315 | SB | - |
| 353.051424 | 14 | 15 | 10 | 0 | 0 | 0 | CHO | 354.0587 | SB | 1 |
| 353.072554 | 18 | 12 | 12 | 0 | 0 | 0 | CHO | 354.07983 | SB | - |
| 353.124194 | 22 | 17 | 8 | 0 | 0 | 0 | CHO | 354.13147 | BB | - |
| 353.181709 | 30 | 15 | 9 | 0 | 0 | 0 | CHO | 354.188985 | BB | - |
| 353.212219 | 30 | 23 | 3 | 0 | 0 | 0 | CHO | 354.219495 | BB | 2 |
| 353.342504 | 46 | 23 | 2 | 0 | 0 | 0 | CHO | 354.34978 | BB | 4 |
| 354.018366 | 13 | 8 | 7 | 5 | 2 | 0 | CHNOS | 355.025642 | SB | - |
| 354.054751 | 17 | 9 | 6 | 5 | 2 | 0 | CHNOS | 355.062027 | SB | 1 |
| 354.077399 | 21 | 15 | 1 | 3 | 3 | 0 | CHNOS | 355.084675 | BB | - |
| 354.083058 | 17 | 15 | 9 | 1 | 0 | 0 | CHNO | 355.090334 | SB | - |
| 354.085261 | 17 | 17 | 0 | 5 | 2 | 0 | CHNOS | 355.092537 | BB | - |
| 354.095627 | 13 | 15 | 4 | 7 | 0 | 0 | CHNO | 355.102903 | BB | - |
| 354.104188 | 21 | 12 | 11 | 1 | 0 | 0 | CHNO | 355.111464 | SB | - |
| 354.119443 | 21 | 16 | 8 | 1 | 0 | 0 | CHNO | 355.126719 | SB | - |
| 354.127521 | 25 | 11 | 4 | 5 | 2 | 0 | CHNOS | 355.134797 | BB | - |
| 355.009775 | 16 | 10 | 6 | 2 | 3 | 0 | CHNOS | 356.017051 | SB | - |
| 355.030689 | 12 | 14 | 11 | 0 | 0 | 0 | CHO | 356.037965 | SB | - |
| 355.058044 | 16 | 18 | 2 | 2 | 2 | 0 | CHNOS | 356.06532 | SB | - |
| 355.067074 | 16 | 15 | 10 | 0 | 0 | 0 | CHO | 356.07435 | SB | - |
| 355.088204 | 20 | 12 | 12 | 0 | 0 | 0 | CHO | 356.09548 | SB | - |
| 355.103459 | 20 | 16 | 9 | 0 | 0 | 0 | CHO | 356.110735 | BB | 3 |
| 355.139844 | 24 | 17 | 8 | 0 | 0 | 0 | CHO | 356.14712 | BB | - |
| 355.194855 | 32 | 19 | 4 | 0 | 1 | 0 | CHOS | 356.202131 | BB | - |
| 355.248999 | 36 | 20 | 5 | 0 | 0 | 0 | CHO | 356.256275 | BB | 9 |
| 356.028141 | 11 | 15 | 2 | 5 | 2 | 0 | CHNOS | 357.035417 | SB | 2 |
| 356.034016 | 15 | 8 | 7 | 5 | 2 | 0 | CHNOS | 357.041292 | SB | - |
| 356.106786 | 23 | 10 | 5 | 5 | 2 | 0 | CHNOS | 357.114062 | BB | 4 |
| 357.009954 | 10 | 13 | 12 | 0 | 0 | 0 | CHO | 358.01723 | SB | - |
| 357.046339 | 14 | 14 | 11 | 0 | 0 | 0 | CHO | 358.053615 | SB | - |
| 357.103854 | 22 | 12 | 12 | 0 | 0 | 0 | CHO | 358.11113 | SB | - |
| 357.134364 | 22 | 20 | 6 | 0 | 0 | 0 | CHO | 358.14164 | SB | 17 |
| 357.140239 | 26 | 13 | 11 | 0 | 0 | 0 | CHO | 358.147515 | SB | - |
| 357.191879 | 30 | 18 | 7 | 0 | 0 | 0 | CHO | 358.199155 | BB | - |
| 357.228264 | 34 | 19 | 6 | 0 | 0 | 0 | CHO | 358.23554 | BB | - |
| 357.264649 | 38 | 20 | 5 | 0 | 0 | 0 | CHO | 358.271925 | BB | 6 |
| 357.301034 | 42 | 21 | 4 | 0 | 0 | 0 | CHO | 358.30831 | BB | 3 |
| 358.025165 | 9 | 14 | 5 | 5 | 1 | 0 | CHNOS | 359.032441 | BB | 2 |
| 358.049666 | 17 | 8 | 7 | 5 | 2 | 0 | CHNOS | 359.056942 | SB | - |
| 358.104461 | 17 | 17 | 6 | 3 | 0 | 0 | CHNO | 359.111737 | BB | - |
| 359.025604 | 12 | 13 | 12 | 0 | 0 | 0 | CHO | 360.03288 | SB | - |
| 359.04423 | 16 | 14 | 9 | 0 | 1 | 0 | CHOS | 360.051506 | SB | - |
| 359.061989 | 16 | 14 | 11 | 0 | 0 | 0 | CHO | 360.069265 | SB | - |
| 359.06536 | 20 | 11 | 11 | 0 | 1 | 0 | CHOS | 360.072636 | SB | 1 |
| 359.077244 | 16 | 18 | 8 | 0 | 0 | 0 | CHO | 360.08452 | BB | 63 |
| 359.119504 | 24 | 12 | 12 | 0 | 0 | 0 | CHO | 360.12678 | SB | - |
| 359.134759 | 24 | 16 | 9 | 0 | 0 | 0 | CHO | 360.142035 | BB | 6 |
| 359.150014 | 24 | 20 | 6 | 0 | 0 | 0 | CHO | 360.15729 | BB | 11 |
| 359.207529 | 32 | 18 | 7 | 0 | 0 | 0 | CHO | 360.214805 | BB | - |
| 359.243914 | 36 | 19 | 6 | 0 | 0 | 0 | CHO | 360.25119 | BB | - |
| 359.280299 | 40 | 20 | 5 | 0 | 0 | 0 | CHO | 360.287575 | BB | - |
| 360.066834 | 19 | 17 | 0 | 3 | 3 | 0 | CHNOS | 361.07411 | BB | - |
| 360.072493 | 15 | 17 | 8 | 1 | 0 | 0 | CHNO | 361.079769 | SB | - |
| 360.0772 | 15 | 15 | 4 | 5 | 1 | 0 | CHNOS | 361.084476 | SB | - |
| 360.085062 | 11 | 17 | 3 | 7 | 0 | 0 | CHNO | 361.092338 | BB | - |
| 360.093623 | 19 | 14 | 10 | 1 | 0 | 0 | CHNO | 361.100899 | SB | - |
| 360.095826 | 19 | 16 | 1 | 5 | 2 | 0 | CHNOS | 361.103102 | BB | - |
| 360.096994 | 23 | 11 | 10 | 1 | 1 | 0 | CHNOS | 361.10427 | SB | - |
| 360.138086 | 27 | 10 | 5 | 5 | 2 | 0 | CHNOS | 361.145362 | BB | 6 |
| 361.020124 | 10 | 16 | 10 | 0 | 0 | 0 | CHO | 362.0274 | SB | - |
| 361.041254 | 14 | 13 | 12 | 0 | 0 | 0 | CHO | 362.04853 | SB | - |
| 361.07198 | 22 | 14 | 3 | 2 | 3 | 0 | CHNOS | 362.079256 | BB | 5 |
| 361.092894 | 18 | 18 | 8 | 0 | 0 | 0 | CHO | 362.10017 | BB | 1 |
| 361.102308 | 18 | 14 | 0 | 8 | 2 | 0 | CHNOS | 362.109584 | SB | - |
| 361.123438 | 22 | 11 | 2 | 8 | 2 | 0 | CHNOS | 362.130714 | SB | - |
| 361.126124 | 26 | 15 | 4 | 2 | 2 | 0 | CHNOS | 362.1334 | SB | - |
| 361.150409 | 26 | 16 | 9 | 0 | 0 | 0 | CHO | 362.157685 | BB | - |
| 361.186794 | 30 | 17 | 8 | 0 | 0 | 0 | CHO | 362.19407 | BB | - |
| 361.27819 | 42 | 20 | 3 | 0 | 1 | 0 | CHOS | 362.285466 | BB | - |
| 362.023451 | 13 | 10 | 6 | 5 | 2 | 0 | CHNOS | 363.030727 | SB | 5 |
| 362.038706 | 13 | 14 | 3 | 5 | 2 | 0 | CHNOS | 363.045982 | SB | - |
| 362.051758 | 13 | 16 | 9 | 1 | 0 | 0 | CHNO | 363.059034 | SB | - |
| 362.088143 | 17 | 17 | 8 | 1 | 0 | 0 | CHNO | 363.095419 | SB | - |
| 362.09285 | 17 | 15 | 4 | 5 | 1 | 0 | CHNOS | 363.100126 | BB | - |
| 362.096221 | 21 | 12 | 4 | 5 | 2 | 0 | CHNOS | 363.103497 | BB | 2 |
| 362.117351 | 25 | 9 | 6 | 5 | 2 | 0 | CHNOS | 363.124627 | BB | - |
| 362.999605 | 16 | 8 | 8 | 2 | 3 | 0 | CHNOS | 364.006881 | SB | - |
| 363.035774 | 12 | 16 | 10 | 0 | 0 | 0 | CHO | 364.04305 | SB | - |
| 363.056904 | 16 | 13 | 12 | 0 | 0 | 0 | CHO | 364.06418 | SB | - |
| 363.111915 | 24 | 15 | 8 | 0 | 1 | 0 | CHOS | 364.119191 | SB | - |
| 363.129674 | 24 | 15 | 10 | 0 | 0 | 0 | CHO | 364.13695 | BB | - |
| 363.144929 | 24 | 19 | 7 | 0 | 0 | 0 | CHO | 364.152205 | BB | 4 |
| 363.238829 | 36 | 18 | 7 | 0 | 0 | 0 | CHO | 364.246105 | BB | - |
| 363.290469 | 40 | 23 | 3 | 0 | 0 | 0 | CHO | 364.297745 | BB | 8 |
| 364.025364 | 15 | 15 | 2 | 3 | 3 | 0 | CHNOS | 365.03264 | SB | - |
| 364.039101 | 15 | 10 | 6 | 5 | 2 | 0 | CHNOS | 365.046377 | SB | - |
| 364.054356 | 15 | 14 | 3 | 5 | 2 | 0 | CHNOS | 365.061632 | SB | - |
| 364.096616 | 23 | 8 | 7 | 5 | 2 | 0 | CHNOS | 365.103892 | BB | - |
| 365.015039 | 10 | 15 | 11 | 0 | 0 | 0 | CHO | 366.022315 | SB | - |
| 365.01841 | 14 | 12 | 11 | 0 | 1 | 0 | CHOS | 366.025686 | SB | - |
| 365.051424 | 14 | 16 | 10 | 0 | 0 | 0 | CHO | 366.0587 | SB | - |
| 365.102413 | 22 | 13 | 8 | 2 | 1 | 0 | CHNOS | 366.109689 | SB | - |
| 365.181709 | 30 | 16 | 9 | 0 | 0 | 0 | CHO | 366.188985 | BB | - |
| 365.200335 | 34 | 17 | 6 | 0 | 1 | 0 | CHOS | 366.207611 | BB | - |
| 365.218094 | 34 | 17 | 8 | 0 | 0 | 0 | CHO | 366.22537 | BB | - |
| 365.23672 | 38 | 18 | 5 | 0 | 1 | 0 | CHOS | 366.243996 | BB | - |
| 366.018366 | 13 | 9 | 7 | 5 | 2 | 0 | CHNOS | 367.025642 | SB | - |
| 366.054751 | 17 | 10 | 6 | 5 | 2 | 0 | CHNOS | 367.062027 | SB | - |
| 366.077399 | 21 | 16 | 1 | 3 | 3 | 0 | CHNOS | 367.084675 | BB | - |
| 366.083058 | 17 | 16 | 9 | 1 | 0 | 0 | CHNO | 367.090334 | SB | 36 |
| 366.095627 | 13 | 16 | 4 | 7 | 0 | 0 | CHNO | 367.102903 | BB | - |
| 366.104188 | 21 | 13 | 11 | 1 | 0 | 0 | CHNO | 367.111464 | SB | - |
| 366.127521 | 25 | 12 | 4 | 5 | 2 | 0 | CHNOS | 367.134797 | BB | - |
| 366.140573 | 25 | 14 | 10 | 1 | 0 | 0 | CHNO | 367.147849 | SB | 15 |
| 366.99452 | 16 | 7 | 9 | 2 | 3 | 0 | CHNOS | 368.001796 | SB | - |
| 367.030689 | 12 | 15 | 11 | 0 | 0 | 0 | CHO | 368.037965 | SB | - |
| 367.03406 | 16 | 12 | 11 | 0 | 1 | 0 | CHOS | 368.041336 | SB | - |
| 367.045944 | 12 | 19 | 8 | 0 | 0 | 0 | CHO | 368.05322 | BB | - |
| 367.048664 | 20 | 8 | 10 | 2 | 2 | 0 | CHNOS | 368.05594 | SB | - |
| 367.051819 | 16 | 12 | 13 | 0 | 0 | 0 | CHO | 368.059095 | SB | - |
| 367.067074 | 16 | 16 | 10 | 0 | 0 | 0 | CHO | 368.07435 | SB | - |
| 367.067941 | 20 | 17 | 5 | 0 | 2 | 0 | CHOS | 368.075217 | SB | - |
| 367.118714 | 20 | 21 | 6 | 0 | 0 | 0 | CHO | 368.12599 | BB | 38 |
| 367.139844 | 24 | 18 | 8 | 0 | 0 | 0 | CHO | 368.14712 | BB | - |
| 367.206773 | 32 | 12 | 1 | 8 | 2 | 0 | CHNOS | 368.214049 | SB | - |
| 367.358154 | 48 | 24 | 2 | 0 | 0 | 0 | CHO | 368.36543 | BB | 10 |
| 368.034016 | 15 | 9 | 7 | 5 | 2 | 0 | CHNOS | 369.041292 | SB | 2 |
| 368.062323 | 15 | 15 | 10 | 1 | 0 | 0 | CHNO | 369.069599 | SB | - |
| 368.070401 | 19 | 10 | 6 | 5 | 2 | 0 | CHNOS | 369.077677 | SB | - |
| 368.083453 | 19 | 12 | 12 | 1 | 0 | 0 | CHNO | 369.090729 | BB | - |
| 368.093049 | 23 | 16 | 1 | 3 | 3 | 0 | CHNOS | 369.100325 | BB | - |
| 368.098708 | 19 | 16 | 9 | 1 | 0 | 0 | CHNO | 369.105984 | SB | - |
| 368.119838 | 23 | 13 | 11 | 1 | 0 | 0 | CHNO | 369.127114 | SB | - |
| 368.143171 | 27 | 12 | 4 | 5 | 2 | 0 | CHNOS | 369.150447 | BB | - |
| 369.009954 | 10 | 14 | 12 | 0 | 0 | 0 | CHO | 370.01723 | SB | - |
| 369.046339 | 14 | 15 | 11 | 0 | 0 | 0 | CHO | 370.053615 | SB | - |
| 369.061594 | 14 | 19 | 8 | 0 | 0 | 0 | CHO | 370.06887 | BB | 5 |
| 369.155494 | 26 | 18 | 8 | 0 | 0 | 0 | CHO | 370.16277 | BB | - |
| 369.191879 | 30 | 19 | 7 | 0 | 0 | 0 | CHO | 370.199155 | BB | - |
| 369.228264 | 34 | 20 | 6 | 0 | 0 | 0 | CHO | 370.23554 | BB | 11 |
| 369.264649 | 38 | 21 | 5 | 0 | 0 | 0 | CHO | 370.271925 | BB | - |
| 370.049666 | 17 | 9 | 7 | 5 | 2 | 0 | CHNOS | 371.056942 | SB | - |
| 371.025604 | 12 | 14 | 12 | 0 | 0 | 0 | CHO | 372.03288 | SB | - |
| 371.061989 | 16 | 15 | 11 | 0 | 0 | 0 | CHO | 372.069265 | SB | 1 |
| 371.077244 | 16 | 19 | 8 | 0 | 0 | 0 | CHO | 372.08452 | BB | 9 |
| 371.083119 | 20 | 12 | 13 | 0 | 0 | 0 | CHO | 372.090395 | SB | - |
| 371.119504 | 24 | 13 | 12 | 0 | 0 | 0 | CHO | 372.12678 | SB | - |
| 371.134759 | 24 | 17 | 9 | 0 | 0 | 0 | CHO | 372.142035 | BB | 3 |
| 371.155889 | 28 | 14 | 11 | 0 | 0 | 0 | CHO | 372.163165 | SB | - |
| 372.023056 | 11 | 15 | 3 | 5 | 2 | 0 | CHNOS | 373.030332 | SB | - |
| 372.044186 | 15 | 12 | 5 | 5 | 2 | 0 | CHNOS | 373.051462 | BB | - |
| 372.093623 | 19 | 15 | 10 | 1 | 0 | 0 | CHNO | 373.100899 | SB | 2 |
| 372.095826 | 19 | 17 | 1 | 5 | 2 | 0 | CHNOS | 373.103102 | BB | - |
| 372.116956 | 23 | 14 | 3 | 5 | 2 | 0 | CHNOS | 373.124232 | BB | - |
| 372.120111 | 19 | 18 | 6 | 3 | 0 | 0 | CHNO | 373.127387 | BB | - |
| 372.138086 | 27 | 11 | 5 | 5 | 2 | 0 | CHNOS | 373.145362 | BB | 3 |
| 373.02034 | 18 | 10 | 7 | 2 | 3 | 0 | CHNOS | 374.027616 | SB | - |
| 373.041254 | 14 | 14 | 12 | 0 | 0 | 0 | CHO | 374.04853 | SB | - |
| 373.077639 | 18 | 15 | 11 | 0 | 0 | 0 | CHO | 374.084915 | SB | - |
| 373.098769 | 22 | 12 | 13 | 0 | 0 | 0 | CHO | 374.106045 | SB | - |
| 373.150409 | 26 | 17 | 9 | 0 | 0 | 0 | CHO | 374.157685 | BB | 7 |
| 373.161642 | 26 | 16 | 8 | 2 | 0 | 0 | CHNO | 374.168918 | SB | - |
| 373.186794 | 30 | 18 | 8 | 0 | 0 | 0 | CHO | 374.19407 | BB | - |
| 373.223179 | 34 | 19 | 7 | 0 | 0 | 0 | CHO | 374.230455 | BB | - |
| 373.259564 | 38 | 20 | 6 | 0 | 0 | 0 | CHO | 374.26684 | BB | - |
| 373.987066 | 9 | 10 | 7 | 5 | 2 | 0 | CHNOS | 374.994342 | SB | - |
| 374.051758 | 13 | 17 | 9 | 1 | 0 | 0 | CHNO | 375.059034 | SB | - |
| 374.09285 | 17 | 16 | 4 | 5 | 1 | 0 | CHNOS | 375.100126 | BB | - |
| 374.096221 | 21 | 13 | 4 | 5 | 2 | 0 | CHNOS | 375.103497 | BB | - |
| 374.109273 | 21 | 15 | 10 | 1 | 0 | 0 | CHNO | 375.116549 | SB | - |
| 374.132606 | 25 | 14 | 3 | 5 | 2 | 0 | CHNOS | 375.139882 | BB | 16 |
| 374.999605 | 16 | 9 | 8 | 2 | 3 | 0 | CHNOS | 376.006881 | SB | - |
| 375.114419 | 24 | 12 | 13 | 0 | 0 | 0 | CHO | 376.121695 | SB | - |
| 375.129674 | 24 | 16 | 10 | 0 | 0 | 0 | CHO | 376.13695 | BB | 4 |
| 375.238829 | 36 | 19 | 7 | 0 | 0 | 0 | CHO | 376.246105 | BB | - |
| 375.29384 | 44 | 21 | 3 | 0 | 1 | 0 | CHOS | 376.301116 | BB | - |
| 376.025364 | 15 | 16 | 2 | 3 | 3 | 0 | CHNOS | 377.03264 | SB | - |
| 376.039101 | 15 | 11 | 6 | 5 | 2 | 0 | CHNOS | 377.046377 | SB | 3 |
| 376.079977 | 11 | 17 | 4 | 7 | 0 | 0 | CHNO | 377.087253 | BB | - |
| 376.090741 | 19 | 16 | 2 | 5 | 2 | 0 | CHNOS | 377.098017 | BB | - |
| 376.111871 | 23 | 13 | 4 | 5 | 2 | 0 | CHNOS | 377.119147 | BB | - |
| 376.124923 | 23 | 15 | 10 | 1 | 0 | 0 | CHNO | 377.132199 | SB | - |
| 376.133001 | 27 | 10 | 6 | 5 | 2 | 0 | CHNOS | 377.140277 | BB | 3 |
| 377.015039 | 10 | 16 | 11 | 0 | 0 | 0 | CHO | 378.022315 | SB | 3 |
| 377.033014 | 18 | 9 | 10 | 2 | 2 | 0 | CHNOS | 378.04029 | SB | - |
| 377.051424 | 14 | 17 | 10 | 0 | 0 | 0 | CHO | 378.0587 | SB | - |
| 377.124194 | 22 | 19 | 8 | 0 | 0 | 0 | CHO | 378.13147 | BB | 1 |
| 377.218094 | 34 | 18 | 8 | 0 | 0 | 0 | CHO | 378.22537 | BB | - |
| 377.254479 | 38 | 19 | 7 | 0 | 0 | 0 | CHO | 378.261755 | BB | - |
| 378.046673 | 13 | 16 | 10 | 1 | 0 | 0 | CHNO | 379.053949 | SB | - |
| 378.054751 | 17 | 11 | 6 | 5 | 2 | 0 | CHNOS | 379.062027 | SB | 2 |
| 378.077399 | 21 | 17 | 1 | 3 | 3 | 0 | CHNOS | 379.084675 | BB | - |
| 378.127521 | 25 | 13 | 4 | 5 | 2 | 0 | CHNOS | 379.134797 | BB | 2 |
| 379.030689 | 12 | 16 | 11 | 0 | 0 | 0 | CHO | 380.037965 | SB | - |
| 379.088204 | 20 | 14 | 12 | 0 | 0 | 0 | CHO | 380.09548 | SB | - |
| 379.139844 | 24 | 19 | 8 | 0 | 0 | 0 | CHO | 380.14712 | BB | - |
| 379.215985 | 36 | 18 | 6 | 0 | 1 | 0 | CHOS | 380.223261 | SB | 4 |
| 380.034016 | 15 | 10 | 7 | 5 | 2 | 0 | CHNOS | 381.041292 | SB | - |
| 380.062323 | 15 | 16 | 10 | 1 | 0 | 0 | CHNO | 381.069599 | SB | - |
| 380.119838 | 23 | 14 | 11 | 1 | 0 | 0 | CHNO | 381.127114 | SB | 5 |
| 380.143171 | 27 | 13 | 4 | 5 | 2 | 0 | CHNOS | 381.150447 | BB | - |
| 381.009954 | 10 | 15 | 12 | 0 | 0 | 0 | CHO | 382.01723 | SB | - |
| 381.046339 | 14 | 16 | 11 | 0 | 0 | 0 | CHO | 382.053615 | SB | - |
| 381.061594 | 14 | 20 | 8 | 0 | 0 | 0 | CHO | 382.06887 | BB | 3 |
| 381.082724 | 18 | 17 | 10 | 0 | 0 | 0 | CHO | 382.09 | SB | - |
| 381.097979 | 18 | 21 | 7 | 0 | 0 | 0 | CHO | 382.105255 | BB | 17 |
| 381.103854 | 22 | 14 | 12 | 0 | 0 | 0 | CHO | 382.11113 | SB | - |
| 381.155494 | 26 | 19 | 8 | 0 | 0 | 0 | CHO | 382.16277 | BB | 1 |
| 381.240182 | 34 | 13 | 3 | 8 | 1 | 0 | CHNOS | 382.247458 | BB | - |
| 381.249394 | 38 | 18 | 8 | 0 | 0 | 0 | CHO | 382.25667 | SB | - |
| 381.264649 | 38 | 22 | 5 | 0 | 0 | 0 | CHO | 382.271925 | BB | 7 |
| 381.373804 | 50 | 25 | 2 | 0 | 0 | 0 | CHO | 382.38108 | BB | 7 |
| 382.035929 | 17 | 15 | 3 | 3 | 3 | 0 | CHNOS | 383.043205 | SB | - |
| 382.043791 | 13 | 17 | 2 | 5 | 2 | 0 | CHNOS | 383.051067 | SB | - |
| 382.049666 | 17 | 10 | 7 | 5 | 2 | 0 | CHNOS | 383.056942 | SB | - |
| 382.077973 | 17 | 16 | 10 | 1 | 0 | 0 | CHNO | 383.085249 | SB | - |
| 382.086051 | 21 | 11 | 6 | 5 | 2 | 0 | CHNOS | 383.093327 | SB | - |
| 382.090542 | 13 | 16 | 5 | 7 | 0 | 0 | CHNO | 383.097818 | BB | - |
| 382.135488 | 25 | 14 | 11 | 1 | 0 | 0 | CHNO | 383.142764 | SB | 16 |
| 383.025604 | 12 | 15 | 12 | 0 | 0 | 0 | CHO | 384.03288 | SB | - |
| 383.061989 | 16 | 16 | 11 | 0 | 0 | 0 | CHO | 384.069265 | SB | - |
| 383.077244 | 16 | 20 | 8 | 0 | 0 | 0 | CHO | 384.08452 | BB | 1 |
| 383.083119 | 20 | 13 | 13 | 0 | 0 | 0 | CHO | 384.090395 | SB | - |
| 383.087826 | 20 | 11 | 9 | 4 | 1 | 0 | CHNOS | 384.095102 | BB | - |
| 383.089344 | 20 | 20 | 2 | 2 | 2 | 0 | CHNOS | 384.09662 | SB | - |
| 383.119504 | 24 | 14 | 12 | 0 | 0 | 0 | CHO | 384.12678 | BB | 1 |
| 383.171144 | 28 | 19 | 8 | 0 | 0 | 0 | CHO | 384.17842 | BB | - |
| 383.192274 | 32 | 16 | 10 | 0 | 0 | 0 | CHO | 384.19955 | BB | - |
| 384.028931 | 15 | 9 | 8 | 5 | 2 | 0 | CHNOS | 385.036207 | SB | - |
| 384.040815 | 11 | 16 | 5 | 5 | 1 | 0 | CHNOS | 385.048091 | SB | 3 |
| 384.051579 | 19 | 15 | 3 | 3 | 3 | 0 | CHNOS | 385.058855 | SB | - |
| 384.065316 | 19 | 10 | 7 | 5 | 2 | 0 | CHNOS | 385.072592 | SB | - |
| 384.093623 | 19 | 16 | 10 | 1 | 0 | 0 | CHNO | 385.100899 | SB | - |
| 384.138086 | 27 | 12 | 5 | 5 | 2 | 0 | CHNOS | 385.145362 | BB | - |
| 385.041254 | 14 | 15 | 12 | 0 | 0 | 0 | CHO | 386.04853 | SB | - |
| 385.065923 | 14 | 15 | 1 | 8 | 2 | 0 | CHNOS | 386.073199 | SB | - |
| 385.077639 | 18 | 16 | 11 | 0 | 0 | 0 | CHO | 386.084915 | SB | 3 |
| 385.092894 | 18 | 20 | 8 | 0 | 0 | 0 | CHO | 386.10017 | BB | 13 |
| 385.135154 | 26 | 14 | 12 | 0 | 0 | 0 | CHO | 386.14243 | SB | - |
| 385.150409 | 26 | 18 | 9 | 0 | 0 | 0 | CHO | 386.157685 | BB | - |
| 385.171539 | 30 | 15 | 11 | 0 | 0 | 0 | CHO | 386.178815 | SB | - |
| 385.259564 | 38 | 21 | 6 | 0 | 0 | 0 | CHO | 386.26684 | BB | 2 |
| 385.295949 | 42 | 22 | 5 | 0 | 0 | 0 | CHO | 386.303225 | BB | - |
| 386.075091 | 17 | 17 | 2 | 5 | 2 | 0 | CHNOS | 387.082367 | BB | - |
| 386.080966 | 21 | 10 | 7 | 5 | 2 | 0 | CHNOS | 387.088242 | SB | 2 |
| 387.056904 | 16 | 15 | 12 | 0 | 0 | 0 | CHO | 388.06418 | SB | - |
| 387.077733 | 20 | 18 | 0 | 4 | 3 | 0 | CHNOS | 388.085009 | SB | - |
| 387.139088 | 24 | 13 | 2 | 8 | 2 | 0 | CHNOS | 388.146364 | SB | - |
| 387.166059 | 28 | 18 | 9 | 0 | 0 | 0 | CHO | 388.173335 | BB | 2 |
| 387.202444 | 32 | 19 | 8 | 0 | 0 | 0 | CHO | 388.20972 | BB | 5 |
| 387.238829 | 36 | 20 | 7 | 0 | 0 | 0 | CHO | 388.246105 | BB | 1 |
| 387.275214 | 40 | 21 | 6 | 0 | 0 | 0 | CHO | 388.28249 | BB | - |
| 388.060231 | 19 | 9 | 8 | 5 | 2 | 0 | CHNOS | 389.067507 | SB | - |
| 388.079977 | 11 | 18 | 4 | 7 | 0 | 0 | CHNO | 389.087253 | BB | - |
| 388.090741 | 19 | 17 | 2 | 5 | 2 | 0 | CHNOS | 389.098017 | BB | - |
| 389.036169 | 14 | 14 | 13 | 0 | 0 | 0 | CHO | 390.043445 | SB | - |
| 389.130069 | 26 | 13 | 13 | 0 | 0 | 0 | CHO | 390.137345 | SB | - |
| 389.145324 | 26 | 17 | 10 | 0 | 0 | 0 | CHO | 390.1526 | BB | 15 |
| 389.218094 | 34 | 19 | 8 | 0 | 0 | 0 | CHO | 390.22537 | BB | 2 |
| 389.254479 | 38 | 20 | 7 | 0 | 0 | 0 | CHO | 390.261755 | BB | - |
| 389.30949 | 46 | 22 | 3 | 0 | 1 | 0 | CHOS | 390.316766 | BB | - |
| 390.041014 | 17 | 17 | 2 | 3 | 3 | 0 | CHNOS | 391.04829 | SB | - |
| 390.05138 | 13 | 15 | 6 | 5 | 1 | 0 | CHNOS | 391.058656 | SB | - |
| 390.077399 | 21 | 18 | 1 | 3 | 3 | 0 | CHNOS | 391.084675 | BB | - |
| 390.083058 | 17 | 18 | 9 | 1 | 0 | 0 | CHNO | 391.090334 | SB | - |
| 390.095627 | 13 | 18 | 4 | 7 | 0 | 0 | CHNO | 391.102903 | BB | - |
| 390.106391 | 21 | 17 | 2 | 5 | 2 | 0 | CHNOS | 391.113667 | BB | - |
| 390.119443 | 21 | 19 | 8 | 1 | 0 | 0 | CHNO | 391.126719 | SB | 41 |
| 390.127521 | 25 | 14 | 4 | 5 | 2 | 0 | CHNOS | 391.134797 | BB | - |
| 390.148651 | 29 | 11 | 6 | 5 | 2 | 0 | CHNOS | 391.155927 | BB | 13 |
| 390.997675 | 12 | 13 | 12 | 0 | 1 | 0 | CHOS | 392.004951 | SB | - |
| 391.030689 | 12 | 17 | 11 | 0 | 0 | 0 | CHO | 392.037965 | SB | - |
| 391.051819 | 16 | 14 | 13 | 0 | 0 | 0 | CHO | 392.059095 | SB | - |
| 391.067074 | 16 | 18 | 10 | 0 | 0 | 0 | CHO | 392.07435 | SB | 1 |
| 391.103459 | 20 | 19 | 9 | 0 | 0 | 0 | CHO | 392.110735 | SB | - |
| 391.109502 | 16 | 18 | 1 | 8 | 1 | 0 | CHNOS | 392.116778 | SB | - |
| 391.124589 | 24 | 16 | 11 | 0 | 0 | 0 | CHO | 392.131865 | BB | 1 |
| 391.139844 | 24 | 20 | 8 | 0 | 0 | 0 | CHO | 392.14712 | BB | 4 |
| 391.176229 | 28 | 21 | 7 | 0 | 0 | 0 | CHO | 392.183505 | BB | 3 |
| 391.197359 | 32 | 18 | 9 | 0 | 0 | 0 | CHO | 392.204635 | BB | - |
| 391.233744 | 36 | 19 | 8 | 0 | 0 | 0 | CHO | 392.24102 | BB | - |
| 391.270129 | 40 | 20 | 7 | 0 | 0 | 0 | CHO | 392.277405 | BB | - |
| 391.321769 | 44 | 25 | 3 | 0 | 0 | 0 | CHO | 392.329045 | BB | - |
| 392.034016 | 15 | 11 | 7 | 5 | 2 | 0 | CHNOS | 393.041292 | SB | 1 |
| 392.049271 | 15 | 15 | 4 | 5 | 2 | 0 | CHNOS | 393.056547 | SB | - |
| 392.062323 | 15 | 17 | 10 | 1 | 0 | 0 | CHNO | 393.069599 | SB | - |
| 392.06703 | 15 | 15 | 6 | 5 | 1 | 0 | CHNOS | 393.074306 | SB | - |
| 392.106786 | 23 | 13 | 5 | 5 | 2 | 0 | CHNOS | 393.114062 | BB | - |
| 392.111277 | 15 | 18 | 4 | 7 | 0 | 0 | CHNO | 393.118553 | BB | - |
| 393.009954 | 10 | 16 | 12 | 0 | 0 | 0 | CHO | 394.01723 | SB | - |
| 393.046339 | 14 | 17 | 11 | 0 | 0 | 0 | CHO | 394.053615 | SB | - |
| 393.119109 | 22 | 19 | 9 | 0 | 0 | 0 | CHO | 394.126385 | BB | 4 |
| 393.140239 | 26 | 16 | 11 | 0 | 0 | 0 | CHO | 394.147515 | BB | - |
| 393.19525 | 34 | 18 | 7 | 0 | 1 | 0 | CHOS | 394.202526 | SB | - |
| 394.049666 | 17 | 11 | 7 | 5 | 2 | 0 | CHNOS | 395.056942 | SB | - |
| 394.114358 | 21 | 18 | 9 | 1 | 0 | 0 | CHNO | 395.121634 | SB | - |
| 394.122436 | 25 | 13 | 5 | 5 | 2 | 0 | CHNOS | 395.129712 | BB | 3 |
| 394.198361 | 29 | 19 | 6 | 3 | 0 | 0 | CHNO | 395.205637 | SB | - |
| 395.025604 | 12 | 16 | 12 | 0 | 0 | 0 | CHO | 396.03288 | SB | - |
| 395.043579 | 20 | 9 | 11 | 2 | 2 | 0 | CHNOS | 396.050855 | SB | - |
| 395.061989 | 16 | 17 | 11 | 0 | 0 | 0 | CHO | 396.069265 | SB | - |
| 395.079964 | 24 | 10 | 10 | 2 | 2 | 0 | CHNOS | 396.08724 | BB | - |
| 395.083119 | 20 | 14 | 13 | 0 | 0 | 0 | CHO | 396.090395 | SB | - |
| 395.134759 | 24 | 19 | 9 | 0 | 0 | 0 | CHO | 396.142035 | BB | - |
| 395.192274 | 32 | 17 | 10 | 0 | 0 | 0 | CHO | 396.19955 | BB | - |
| 395.2109 | 36 | 18 | 7 | 0 | 1 | 0 | CHOS | 396.218176 | SB | - |
| 395.228659 | 36 | 18 | 9 | 0 | 0 | 0 | CHO | 396.235935 | BB | - |
| 395.243914 | 36 | 22 | 6 | 0 | 0 | 0 | CHO | 396.25119 | BB | 2 |
| 395.280299 | 40 | 23 | 5 | 0 | 0 | 0 | CHO | 396.287575 | BB | 2 |
| 395.389454 | 52 | 26 | 2 | 0 | 0 | 0 | CHO | 396.39673 | BB | 11 |
| 396.028931 | 15 | 10 | 8 | 5 | 2 | 0 | CHNOS | 397.036207 | SB | - |
| 396.065316 | 19 | 11 | 7 | 5 | 2 | 0 | CHNOS | 397.072592 | SB | - |
| 396.086446 | 23 | 8 | 9 | 5 | 2 | 0 | CHNOS | 397.093722 | SB | - |
| 396.087964 | 23 | 17 | 2 | 3 | 3 | 0 | CHNOS | 397.09524 | BB | - |
| 396.093623 | 19 | 17 | 10 | 1 | 0 | 0 | CHNO | 397.100899 | SB | - |
| 396.130008 | 23 | 18 | 9 | 1 | 0 | 0 | CHNO | 397.137284 | SB | - |
| 396.138086 | 27 | 13 | 5 | 5 | 2 | 0 | CHNOS | 397.145362 | BB | - |
| 396.151138 | 27 | 15 | 11 | 1 | 0 | 0 | CHNO | 397.158414 | SB | - |
| 396.348318 | 47 | 24 | 3 | 1 | 0 | 0 | CHNO | 397.355594 | BB | 1 |
| 397.041254 | 14 | 16 | 12 | 0 | 0 | 0 | CHO | 398.04853 | SB | - |
| 397.077639 | 18 | 17 | 11 | 0 | 0 | 0 | CHO | 398.084915 | SB | - |
| 397.150409 | 26 | 19 | 9 | 0 | 0 | 0 | CHO | 398.157685 | BB | - |
| 397.223179 | 34 | 21 | 7 | 0 | 0 | 0 | CHO | 398.230455 | BB | - |
| 398.038706 | 13 | 17 | 3 | 5 | 2 | 0 | CHNOS | 399.045982 | SB | - |
| 398.044581 | 17 | 10 | 8 | 5 | 2 | 0 | CHNOS | 399.051857 | SB | - |
| 398.056465 | 13 | 17 | 5 | 5 | 1 | 0 | CHNOS | 399.063741 | SB | - |
| 398.080966 | 21 | 11 | 7 | 5 | 2 | 0 | CHNOS | 399.088242 | SB | - |
| 398.109273 | 21 | 17 | 10 | 1 | 0 | 0 | CHNO | 399.116549 | SB | - |
| 399.020519 | 12 | 15 | 13 | 0 | 0 | 0 | CHO | 400.027795 | SB | - |
| 399.056904 | 16 | 16 | 12 | 0 | 0 | 0 | CHO | 400.06418 | SB | - |
| 399.114419 | 24 | 14 | 13 | 0 | 0 | 0 | CHO | 400.121695 | SB | - |
| 399.150804 | 28 | 15 | 12 | 0 | 0 | 0 | CHO | 400.15808 | SB | - |
| 399.166059 | 28 | 19 | 9 | 0 | 0 | 0 | CHO | 400.173335 | SB | - |
| 399.238829 | 36 | 21 | 7 | 0 | 0 | 0 | CHO | 400.246105 | BB | - |
| 400.03573 | 11 | 16 | 6 | 5 | 1 | 0 | CHNOS | 401.043006 | SB | - |
| 400.060231 | 19 | 10 | 8 | 5 | 2 | 0 | CHNOS | 401.067507 | SB | - |
| 400.090741 | 19 | 18 | 2 | 5 | 2 | 0 | CHNOS | 401.098017 | BB | - |
| 400.124923 | 23 | 17 | 10 | 1 | 0 | 0 | CHNO | 401.132199 | SB | - |
| 400.133001 | 27 | 12 | 6 | 5 | 2 | 0 | CHNOS | 401.140277 | BB | - |
| 400.146053 | 27 | 14 | 12 | 1 | 0 | 0 | CHNO | 401.153329 | SB | - |
| 400.97887 | 14 | 10 | 9 | 2 | 3 | 0 | CHNOS | 401.986146 | SB | - |
| 401.015255 | 18 | 11 | 8 | 2 | 3 | 0 | CHNOS | 402.022531 | SB | - |
| 401.072554 | 18 | 16 | 12 | 0 | 0 | 0 | CHO | 402.07983 | SB | - |
| 401.087809 | 18 | 20 | 9 | 0 | 0 | 0 | CHO | 402.095085 | BB | 11 |
| 401.130069 | 26 | 14 | 13 | 0 | 0 | 0 | CHO | 402.137345 | SB | - |
| 401.160579 | 26 | 22 | 7 | 0 | 0 | 0 | CHO | 402.167855 | BB | 3 |
| 401.254479 | 38 | 21 | 7 | 0 | 0 | 0 | CHO | 402.261755 | BB | 1 |
| 402.018366 | 13 | 12 | 7 | 5 | 2 | 0 | CHNOS | 403.025642 | SB | - |
| 402.075881 | 21 | 10 | 8 | 5 | 2 | 0 | CHNOS | 403.083157 | SB | - |
| 402.083058 | 17 | 19 | 9 | 1 | 0 | 0 | CHNO | 403.090334 | SB | - |
| 402.091136 | 21 | 14 | 5 | 5 | 2 | 0 | CHNOS | 403.098412 | BB | 11 |
| 402.104188 | 21 | 16 | 11 | 1 | 0 | 0 | CHNO | 403.111464 | SB | - |
| 402.148651 | 29 | 12 | 6 | 5 | 2 | 0 | CHNOS | 403.155927 | BB | - |
| 402.99452 | 16 | 10 | 9 | 2 | 3 | 0 | CHNOS | 404.001796 | SB | 2 |
| 403.030689 | 12 | 18 | 11 | 0 | 0 | 0 | CHO | 404.037965 | SB | - |
| 403.051819 | 16 | 15 | 13 | 0 | 0 | 0 | CHO | 404.059095 | SB | - |
| 403.088204 | 20 | 16 | 12 | 0 | 0 | 0 | CHO | 404.09548 | SB | - |
| 403.103459 | 20 | 20 | 9 | 0 | 0 | 0 | CHO | 404.110735 | BB | 23 |
| 403.134003 | 24 | 13 | 3 | 8 | 2 | 0 | CHNOS | 404.141279 | SB | - |
| 403.139844 | 24 | 21 | 8 | 0 | 0 | 0 | CHO | 404.14712 | BB | 4 |
| 403.145719 | 28 | 14 | 13 | 0 | 0 | 0 | CHO | 404.152995 | SB | - |
| 403.233744 | 36 | 20 | 8 | 0 | 0 | 0 | CHO | 404.24102 | BB | - |
| 403.32514 | 48 | 23 | 3 | 0 | 1 | 0 | CHOS | 404.332416 | BB | - |
| 404.062323 | 15 | 18 | 10 | 1 | 0 | 0 | CHNO | 405.069599 | SB | - |
| 404.091531 | 23 | 10 | 8 | 5 | 2 | 0 | CHNOS | 405.098807 | SB | - |
| 404.098708 | 19 | 19 | 9 | 1 | 0 | 0 | CHNO | 405.105984 | SB | - |
| 404.119838 | 23 | 16 | 11 | 1 | 0 | 0 | CHNO | 405.127114 | SB | - |
| 405.01017 | 18 | 10 | 9 | 2 | 3 | 0 | CHNOS | 406.017446 | SB | - |
| 405.013325 | 14 | 14 | 12 | 0 | 1 | 0 | CHOS | 406.020601 | SB | - |
| 405.046339 | 14 | 18 | 11 | 0 | 0 | 0 | CHO | 406.053615 | SB | - |
| 405.067469 | 18 | 15 | 13 | 0 | 0 | 0 | CHO | 406.074745 | SB | - |
| 405.103854 | 22 | 16 | 12 | 0 | 0 | 0 | CHO | 406.11113 | SB | - |
| 405.119109 | 22 | 20 | 9 | 0 | 0 | 0 | CHO | 406.126385 | BB | 7 |
| 405.155494 | 26 | 21 | 8 | 0 | 0 | 0 | CHO | 406.16277 | BB | - |
| 405.176624 | 30 | 18 | 10 | 0 | 0 | 0 | CHO | 406.1839 | SB | - |
| 405.213009 | 34 | 19 | 9 | 0 | 0 | 0 | CHO | 406.220285 | BB | - |
| 406.035929 | 17 | 17 | 3 | 3 | 3 | 0 | CHNOS | 407.043205 | SB | - |
| 406.049666 | 17 | 12 | 7 | 5 | 2 | 0 | CHNOS | 407.056942 | SB | - |
| 406.064921 | 17 | 16 | 4 | 5 | 2 | 0 | CHNOS | 407.072197 | SB | - |
| 406.090542 | 13 | 18 | 5 | 7 | 0 | 0 | CHNO | 407.097818 | BB | - |
| 406.107181 | 25 | 10 | 8 | 5 | 2 | 0 | CHNOS | 407.114457 | SB | - |
| 407.025604 | 12 | 17 | 12 | 0 | 0 | 0 | CHO | 408.03288 | SB | - |
| 407.061989 | 16 | 18 | 11 | 0 | 0 | 0 | CHO | 408.069265 | SB | - |
| 407.08649 | 24 | 12 | 13 | 0 | 1 | 0 | CHOS | 408.093766 | SB | - |
| 407.134759 | 24 | 20 | 9 | 0 | 0 | 0 | CHO | 408.142035 | BB | 3 |
| 407.192274 | 32 | 18 | 10 | 0 | 0 | 0 | CHO | 408.19955 | BB | - |
| 408.028931 | 15 | 11 | 8 | 5 | 2 | 0 | CHNOS | 409.036207 | SB | - |
| 408.057238 | 15 | 17 | 11 | 1 | 0 | 0 | CHNO | 409.064514 | SB | - |
| 408.065316 | 19 | 12 | 7 | 5 | 2 | 0 | CHNOS | 409.072592 | SB | - |
| 408.093623 | 19 | 18 | 10 | 1 | 0 | 0 | CHNO | 409.100899 | SB | - |
| 408.103037 | 19 | 14 | 2 | 9 | 2 | 0 | CHNOS | 409.110313 | SB | - |
| 408.106192 | 15 | 18 | 5 | 7 | 0 | 0 | CHNO | 409.113468 | BB | - |
| 408.130008 | 23 | 19 | 9 | 1 | 0 | 0 | CHNO | 409.137284 | SB | - |
| 408.138086 | 27 | 14 | 5 | 5 | 2 | 0 | CHNOS | 409.145362 | BB | 3 |
| 408.151138 | 27 | 16 | 11 | 1 | 0 | 0 | CHNO | 409.158414 | SB | 2 |
| 409.004869 | 10 | 16 | 13 | 0 | 0 | 0 | CHO | 410.012145 | SB | - |
| 409.041254 | 14 | 17 | 12 | 0 | 0 | 0 | CHO | 410.04853 | SB | - |
| 409.065755 | 22 | 11 | 14 | 0 | 1 | 0 | CHOS | 410.073031 | SB | - |
| 409.077639 | 18 | 18 | 11 | 0 | 0 | 0 | CHO | 410.084915 | SB | - |
| 409.190165 | 34 | 18 | 8 | 0 | 1 | 0 | CHOS | 410.197441 | SB | - |
| 410.038706 | 13 | 18 | 3 | 5 | 2 | 0 | CHNOS | 411.045982 | SB | - |
| 410.044581 | 17 | 11 | 8 | 5 | 2 | 0 | CHNOS | 411.051857 | SB | - |
| 410.048603 | 17 | 16 | 6 | 3 | 2 | 0 | CHNOS | 411.055879 | BB | - |
| 410.080966 | 21 | 12 | 7 | 5 | 2 | 0 | CHNOS | 411.088242 | SB | - |
| 410.103614 | 25 | 18 | 2 | 3 | 3 | 0 | CHNOS | 411.11089 | BB | - |
| 411.020519 | 12 | 16 | 13 | 0 | 0 | 0 | CHO | 412.027795 | SB | - |
| 411.056904 | 16 | 17 | 12 | 0 | 0 | 0 | CHO | 412.06418 | SB | - |
| 411.093289 | 20 | 18 | 11 | 0 | 0 | 0 | CHO | 412.100565 | SB | - |
| 411.108544 | 20 | 22 | 8 | 0 | 0 | 0 | CHO | 412.11582 | BB | 4 |
| 411.129674 | 24 | 19 | 10 | 0 | 0 | 0 | CHO | 412.13695 | BB | - |
| 411.166059 | 28 | 20 | 9 | 0 | 0 | 0 | CHO | 412.173335 | BB | - |
| 411.232988 | 36 | 14 | 2 | 8 | 2 | 0 | CHNOS | 412.240264 | SB | - |
| 412.060231 | 19 | 11 | 8 | 5 | 2 | 0 | CHNOS | 413.067507 | SB | - |
| 412.064253 | 19 | 16 | 6 | 3 | 2 | 0 | CHNOS | 413.071529 | SB | - |
| 412.096616 | 23 | 12 | 7 | 5 | 2 | 0 | CHNOS | 413.103892 | SB | - |
| 413.036169 | 14 | 16 | 13 | 0 | 0 | 0 | CHO | 414.043445 | SB | - |
| 413.072554 | 18 | 17 | 12 | 0 | 0 | 0 | CHO | 414.07983 | SB | - |
| 413.087809 | 18 | 21 | 9 | 0 | 0 | 0 | CHO | 414.095085 | BB | 4 |
| 413.099909 | 22 | 21 | 3 | 2 | 2 | 0 | CHNOS | 414.107185 | SB | - |
| 413.166454 | 30 | 16 | 12 | 0 | 0 | 0 | CHO | 414.17373 | SB | - |
| 413.181709 | 30 | 20 | 9 | 0 | 0 | 0 | CHO | 414.188985 | BB | - |
| 413.290864 | 42 | 23 | 6 | 0 | 0 | 0 | CHO | 414.29814 | BB | - |
| 413.363634 | 50 | 25 | 4 | 0 | 0 | 0 | CHO | 414.37091 | BB | 2 |
| 414.039496 | 17 | 10 | 9 | 5 | 2 | 0 | CHNOS | 415.046772 | SB | - |
| 414.05138 | 13 | 17 | 6 | 5 | 1 | 0 | CHNOS | 415.058656 | SB | - |
| 414.075881 | 21 | 11 | 8 | 5 | 2 | 0 | CHNOS | 415.083157 | SB | - |
| 414.99452 | 16 | 11 | 9 | 2 | 3 | 0 | CHNOS | 416.001796 | SB | 4 |
| 415.030689 | 12 | 19 | 11 | 0 | 0 | 0 | CHO | 416.037965 | SB | - |
| 415.051819 | 16 | 16 | 13 | 0 | 0 | 0 | CHO | 416.059095 | SB | - |
| 415.05519 | 20 | 13 | 13 | 0 | 1 | 0 | CHOS | 416.062466 | BB | - |
| 415.088204 | 20 | 17 | 12 | 0 | 0 | 0 | CHO | 416.09548 | SB | - |
| 415.139844 | 24 | 22 | 8 | 0 | 0 | 0 | CHO | 416.14712 | BB | 3 |
| 415.182104 | 32 | 16 | 12 | 0 | 0 | 0 | CHO | 416.18938 | SB | - |
| 415.306514 | 44 | 23 | 6 | 0 | 0 | 0 | CHO | 416.31379 | BB | - |
| 416.034016 | 15 | 13 | 7 | 5 | 2 | 0 | CHNOS | 417.041292 | SB | - |
| 416.049271 | 15 | 17 | 4 | 5 | 2 | 0 | CHNOS | 417.056547 | SB | - |
| 416.055146 | 19 | 10 | 9 | 5 | 2 | 0 | CHNOS | 417.062422 | SB | - |
| 416.091531 | 23 | 11 | 8 | 5 | 2 | 0 | CHNOS | 417.098807 | SB | - |
| 416.098708 | 19 | 20 | 9 | 1 | 0 | 0 | CHNO | 417.105984 | SB | - |
| 416.103415 | 19 | 18 | 5 | 5 | 1 | 0 | CHNOS | 417.110691 | BB | - |
| 416.106786 | 23 | 15 | 5 | 5 | 2 | 0 | CHNOS | 417.114062 | BB | - |
| 416.143171 | 27 | 16 | 4 | 5 | 2 | 0 | CHNOS | 417.150447 | BB | 3 |
| 417.01017 | 18 | 11 | 9 | 2 | 3 | 0 | CHNOS | 418.017446 | SB | - |
| 417.046339 | 14 | 19 | 11 | 0 | 0 | 0 | CHO | 418.053615 | SB | - |
| 417.067469 | 18 | 16 | 13 | 0 | 0 | 0 | CHO | 418.074745 | SB | - |
| 417.155494 | 26 | 22 | 8 | 0 | 0 | 0 | CHO | 418.16277 | BB | 3 |
| 417.19525 | 34 | 20 | 7 | 0 | 1 | 0 | CHOS | 418.202526 | SB | - |
| 417.213009 | 34 | 20 | 9 | 0 | 0 | 0 | CHO | 418.220285 | BB | - |
| 417.249394 | 38 | 21 | 8 | 0 | 0 | 0 | CHO | 418.25667 | BB | - |
| 418.046295 | 13 | 16 | 7 | 5 | 1 | 0 | CHNOS | 419.053571 | SB | - |
| 418.049666 | 17 | 13 | 7 | 5 | 2 | 0 | CHNOS | 419.056942 | SB | - |
| 418.053688 | 17 | 18 | 5 | 3 | 2 | 0 | CHNOS | 419.060964 | SB | - |
| 418.064921 | 17 | 17 | 4 | 5 | 2 | 0 | CHNOS | 419.072197 | SB | - |
| 418.077973 | 17 | 19 | 10 | 1 | 0 | 0 | CHNO | 419.085249 | SB | - |
| 418.114358 | 21 | 20 | 9 | 1 | 0 | 0 | CHNO | 419.121634 | SB | - |
| 418.122436 | 25 | 15 | 5 | 5 | 2 | 0 | CHNOS | 419.129712 | BB | - |
| 418.135488 | 25 | 17 | 11 | 1 | 0 | 0 | CHNO | 419.142764 | SB | - |
| 419.025604 | 12 | 18 | 12 | 0 | 0 | 0 | CHO | 420.03288 | SB | - |
| 419.028975 | 16 | 15 | 12 | 0 | 1 | 0 | CHOS | 420.036251 | SB | - |
| 419.061989 | 16 | 19 | 11 | 0 | 0 | 0 | CHO | 420.069265 | SB | - |
| 419.08649 | 24 | 13 | 13 | 0 | 1 | 0 | CHOS | 420.093766 | SB | - |
| 419.140634 | 28 | 14 | 14 | 0 | 0 | 0 | CHO | 420.14791 | SB | - |
| 419.2109 | 36 | 20 | 7 | 0 | 1 | 0 | CHOS | 420.218176 | SB | - |
| 419.228659 | 36 | 20 | 9 | 0 | 0 | 0 | CHO | 420.235935 | BB | - |
| 419.265044 | 40 | 21 | 8 | 0 | 0 | 0 | CHO | 420.27232 | BB | - |
| 420.028931 | 15 | 12 | 8 | 5 | 2 | 0 | CHNOS | 421.036207 | SB | - |
| 420.065316 | 19 | 13 | 7 | 5 | 2 | 0 | CHNOS | 421.072592 | SB | - |
| 420.080571 | 19 | 17 | 4 | 5 | 2 | 0 | CHNOS | 421.087847 | SB | - |
| 420.093623 | 19 | 19 | 10 | 1 | 0 | 0 | CHNO | 421.100899 | SB | - |
| 420.116956 | 23 | 18 | 3 | 5 | 2 | 0 | CHNOS | 421.124232 | BB | - |
| 420.130008 | 23 | 20 | 9 | 1 | 0 | 0 | CHNO | 421.137284 | SB | - |
| 421.005085 | 18 | 10 | 10 | 2 | 3 | 0 | CHNOS | 422.012361 | SB | - |
| 421.041254 | 14 | 18 | 12 | 0 | 0 | 0 | CHO | 422.04853 | SB | - |
| 421.077639 | 18 | 19 | 11 | 0 | 0 | 0 | CHO | 422.084915 | SB | 8 |
| 421.10214 | 26 | 13 | 13 | 0 | 1 | 0 | CHOS | 422.109416 | SB | - |
| 421.150409 | 26 | 21 | 9 | 0 | 0 | 0 | CHO | 422.157685 | BB | - |
| 421.182772 | 30 | 17 | 10 | 2 | 0 | 0 | CHNO | 422.190048 | SB | - |
| 421.207924 | 34 | 19 | 10 | 0 | 0 | 0 | CHO | 422.2152 | BB | 1 |
| 421.22655 | 38 | 20 | 7 | 0 | 1 | 0 | CHOS | 422.233826 | SB | 1 |
| 422.044581 | 17 | 12 | 8 | 5 | 2 | 0 | CHNOS | 423.051857 | SB | - |
| 422.072888 | 17 | 18 | 11 | 1 | 0 | 0 | CHNO | 423.080164 | SB | - |
| 422.080966 | 21 | 13 | 7 | 5 | 2 | 0 | CHNOS | 423.088242 | SB | 6 |
| 422.103614 | 25 | 19 | 2 | 3 | 3 | 0 | CHNOS | 423.11089 | BB | - |
| 422.153736 | 29 | 15 | 5 | 5 | 2 | 0 | CHNOS | 423.161012 | BB | - |
| 422.229661 | 33 | 21 | 6 | 3 | 0 | 0 | CHNO | 423.236937 | SB | 1 |
| 423.020519 | 12 | 17 | 13 | 0 | 0 | 0 | CHO | 424.027795 | SB | - |
| 423.039145 | 16 | 18 | 10 | 0 | 1 | 0 | CHOS | 424.046421 | SB | 7 |
| 423.056904 | 16 | 18 | 12 | 0 | 0 | 0 | CHO | 424.06418 | SB | - |
| 423.072159 | 16 | 22 | 9 | 0 | 0 | 0 | CHO | 424.079435 | BB | 1 |
| 423.081405 | 24 | 12 | 14 | 0 | 1 | 0 | CHOS | 424.088681 | SB | - |
| 423.093289 | 20 | 19 | 11 | 0 | 0 | 0 | CHO | 424.100565 | SB | - |
| 423.108544 | 20 | 23 | 8 | 0 | 0 | 0 | CHO | 424.11582 | BB | 2 |
| 423.994854 | 15 | 12 | 8 | 3 | 3 | 0 | CHNOS | 425.00213 | SB | - |
| 424.046494 | 19 | 17 | 4 | 3 | 3 | 0 | CHNOS | 425.05377 | SB | - |
| 424.060231 | 19 | 12 | 8 | 5 | 2 | 0 | CHNOS | 425.067507 | SB | - |
| 424.088538 | 19 | 18 | 11 | 1 | 0 | 0 | CHNO | 425.095814 | SB | - |
| 424.096616 | 23 | 13 | 7 | 5 | 2 | 0 | CHNOS | 425.103892 | SB | - |
| 425.036169 | 14 | 17 | 13 | 0 | 0 | 0 | CHO | 426.043445 | SB | - |
| 425.072554 | 18 | 18 | 12 | 0 | 0 | 0 | CHO | 426.07983 | SB | - |
| 425.087809 | 18 | 22 | 9 | 0 | 0 | 0 | CHO | 426.095085 | BB | 2 |
| 425.18508 | 34 | 18 | 9 | 0 | 1 | 0 | CHOS | 426.192356 | SB | - |
| 425.202839 | 34 | 18 | 11 | 0 | 0 | 0 | CHO | 426.210115 | BB | - |
| 425.254479 | 38 | 23 | 7 | 0 | 0 | 0 | CHO | 426.261755 | BB | 9 |
| 426.033621 | 13 | 18 | 4 | 5 | 2 | 0 | CHNOS | 427.040897 | SB | - |
| 426.05138 | 13 | 18 | 6 | 5 | 1 | 0 | CHNOS | 427.058656 | SB | - |
| 426.062144 | 21 | 17 | 4 | 3 | 3 | 0 | CHNOS | 427.06942 | SB | - |
| 426.075881 | 21 | 12 | 8 | 5 | 2 | 0 | CHNOS | 427.083157 | SB | - |
| 426.080372 | 13 | 17 | 7 | 7 | 0 | 0 | CHNO | 427.087648 | SB | - |
| 426.098529 | 25 | 18 | 3 | 3 | 3 | 0 | CHNOS | 427.105805 | BB | - |
| 426.104188 | 21 | 18 | 11 | 1 | 0 | 0 | CHNO | 427.111464 | SB | - |
| 426.140573 | 25 | 19 | 10 | 1 | 0 | 0 | CHNO | 427.147849 | SB | 3 |
| 427.051819 | 16 | 17 | 13 | 0 | 0 | 0 | CHO | 428.059095 | SB | - |
| 427.088204 | 20 | 18 | 12 | 0 | 0 | 0 | CHO | 428.09548 | SB | - |
| 427.103459 | 20 | 22 | 9 | 0 | 0 | 0 | CHO | 428.110735 | BB | 3 |
| 427.20073 | 36 | 18 | 9 | 0 | 1 | 0 | CHOS | 428.208006 | SB | - |
| 428.049271 | 15 | 18 | 4 | 5 | 2 | 0 | CHNOS | 429.056547 | SB | - |
| 428.055146 | 19 | 11 | 9 | 5 | 2 | 0 | CHNOS | 429.062422 | SB | - |
| 428.077794 | 23 | 17 | 4 | 3 | 3 | 0 | CHNOS | 429.08507 | SB | - |
| 428.091531 | 23 | 12 | 8 | 5 | 2 | 0 | CHNOS | 429.098807 | SB | - |
| 428.103415 | 19 | 19 | 5 | 5 | 1 | 0 | CHNOS | 429.110691 | BB | - |
| 428.164301 | 31 | 14 | 6 | 5 | 2 | 0 | CHNOS | 429.171577 | BB | - |
| 429.01017 | 18 | 12 | 9 | 2 | 3 | 0 | CHNOS | 430.017446 | SB | - |
| 429.056921 | 18 | 11 | 12 | 4 | 1 | 0 | CHNOS | 430.064197 | SB | - |
| 429.067469 | 18 | 17 | 13 | 0 | 0 | 0 | CHO | 430.074745 | SB | - |
| 429.152339 | 30 | 19 | 5 | 2 | 2 | 0 | CHNOS | 430.159615 | SB | - |
| 429.155494 | 26 | 23 | 8 | 0 | 0 | 0 | CHO | 430.16277 | BB | 4 |
| 429.161369 | 30 | 16 | 13 | 0 | 0 | 0 | CHO | 430.168645 | SB | - |
| 429.176624 | 30 | 20 | 10 | 0 | 0 | 0 | CHO | 430.1839 | BB | - |
| 429.213009 | 34 | 21 | 9 | 0 | 0 | 0 | CHO | 430.220285 | BB | - |
| 429.21638 | 38 | 18 | 9 | 0 | 1 | 0 | CHOS | 430.223656 | SB | - |
| 430.013281 | 13 | 13 | 8 | 5 | 2 | 0 | CHNOS | 431.020557 | SB | - |
| 430.046295 | 13 | 17 | 7 | 5 | 1 | 0 | CHNOS | 431.053571 | SB | - |
| 430.049666 | 17 | 14 | 7 | 5 | 2 | 0 | CHNOS | 431.056942 | SB | 1 |
| 430.070796 | 21 | 11 | 9 | 5 | 2 | 0 | CHNOS | 431.078072 | SB | - |
| 430.119065 | 21 | 19 | 5 | 5 | 1 | 0 | CHNOS | 431.126341 | BB | - |
| 430.122436 | 25 | 16 | 5 | 5 | 2 | 0 | CHNOS | 431.129712 | BB | - |
| 430.135488 | 25 | 18 | 11 | 1 | 0 | 0 | CHNO | 431.142764 | SB | - |
| 431.025604 | 12 | 19 | 12 | 0 | 0 | 0 | CHO | 432.03288 | SB | - |
| 431.046734 | 16 | 16 | 14 | 0 | 0 | 0 | CHO | 432.05401 | SB | 1 |
| 431.061989 | 16 | 20 | 11 | 0 | 0 | 0 | CHO | 432.069265 | SB | - |
| 431.072571 | 20 | 11 | 12 | 4 | 1 | 0 | CHNOS | 432.079847 | SB | - |
| 431.083119 | 20 | 17 | 13 | 0 | 0 | 0 | CHO | 432.090395 | SB | - |
| 431.08649 | 24 | 14 | 13 | 0 | 1 | 0 | CHOS | 432.093766 | SB | - |
| 431.088477 | 16 | 23 | 7 | 2 | 0 | 0 | CHNO | 432.095753 | BB | - |
| 432.028931 | 15 | 13 | 8 | 5 | 2 | 0 | CHNOS | 433.036207 | SB | - |
| 432.051579 | 19 | 19 | 3 | 3 | 3 | 0 | CHNOS | 433.058855 | SB | - |
| 432.057238 | 15 | 19 | 11 | 1 | 0 | 0 | CHNO | 433.064514 | SB | 5 |
| 432.061945 | 15 | 17 | 7 | 5 | 1 | 0 | CHNOS | 433.069221 | SB | - |
| 432.065316 | 19 | 14 | 7 | 5 | 2 | 0 | CHNOS | 433.072592 | SB | - |
| 432.086446 | 23 | 11 | 9 | 5 | 2 | 0 | CHNOS | 433.093722 | SB | - |
| 432.093623 | 19 | 20 | 10 | 1 | 0 | 0 | CHNO | 433.100899 | SB | - |
| 432.151138 | 27 | 18 | 11 | 1 | 0 | 0 | CHNO | 433.158414 | SB | - |
| 433.041254 | 14 | 19 | 12 | 0 | 0 | 0 | CHO | 434.04853 | SB | - |
| 433.062384 | 18 | 16 | 14 | 0 | 0 | 0 | CHO | 434.06966 | SB | - |
| 433.077639 | 18 | 20 | 11 | 0 | 0 | 0 | CHO | 434.084915 | SB | 17 |
| 433.114024 | 22 | 21 | 10 | 0 | 0 | 0 | CHO | 434.1213 | BB | 33 |
| 433.119598 | 26 | 20 | 1 | 4 | 3 | 0 | CHNOS | 434.126874 | SB | - |
| 433.150409 | 26 | 22 | 9 | 0 | 0 | 0 | CHO | 434.157685 | BB | 8 |
| 433.156284 | 30 | 15 | 14 | 0 | 0 | 0 | CHO | 434.16356 | SB | 2 |
| 433.22655 | 38 | 21 | 7 | 0 | 1 | 0 | CHOS | 434.233826 | SB | - |
| 434.044581 | 17 | 13 | 8 | 5 | 2 | 0 | CHNOS | 435.051857 | SB | 1 |
| 434.048603 | 17 | 18 | 6 | 3 | 2 | 0 | CHNOS | 435.055879 | SB | - |
| 434.077595 | 17 | 17 | 7 | 5 | 1 | 0 | CHNOS | 435.084871 | SB | - |
| 434.080966 | 21 | 14 | 7 | 5 | 2 | 0 | CHNOS | 435.088242 | SB | 16 |
| 434.109273 | 21 | 20 | 10 | 1 | 0 | 0 | CHNO | 435.116549 | SB | 19 |
| 434.121842 | 17 | 20 | 5 | 7 | 0 | 0 | CHNO | 435.129118 | BB | - |
| 434.153736 | 29 | 16 | 5 | 5 | 2 | 0 | CHNOS | 435.161012 | BB | 9 |
| 435.020519 | 12 | 18 | 13 | 0 | 0 | 0 | CHO | 436.027795 | SB | - |
| 435.056904 | 16 | 19 | 12 | 0 | 0 | 0 | CHO | 436.06418 | SB | - |
| 435.081405 | 24 | 13 | 14 | 0 | 1 | 0 | CHOS | 436.088681 | SB | - |
| 435.129674 | 24 | 21 | 10 | 0 | 0 | 0 | CHO | 436.13695 | BB | 16 |
| 435.166059 | 28 | 22 | 9 | 0 | 0 | 0 | CHO | 436.173335 | BB | - |
| 435.205815 | 36 | 20 | 8 | 0 | 1 | 0 | CHOS | 436.213091 | SB | - |
| 436.023846 | 15 | 12 | 9 | 5 | 2 | 0 | CHNOS | 437.031122 | SB | - |
| 436.060231 | 19 | 13 | 8 | 5 | 2 | 0 | CHNOS | 437.067507 | SB | - |
| 436.088538 | 19 | 19 | 11 | 1 | 0 | 0 | CHNO | 437.095814 | SB | - |
| 437.036169 | 14 | 18 | 13 | 0 | 0 | 0 | CHO | 438.043445 | SB | - |
| 437.054144 | 22 | 11 | 12 | 2 | 2 | 0 | CHNOS | 438.06142 | SB | - |
| 437.06067 | 22 | 12 | 15 | 0 | 1 | 0 | CHOS | 438.067946 | SB | - |
| 437.072554 | 18 | 19 | 12 | 0 | 0 | 0 | CHO | 438.07983 | SB | - |
| 437.145324 | 26 | 21 | 10 | 0 | 0 | 0 | CHO | 438.1526 | BB | 3 |
| 437.221465 | 38 | 20 | 8 | 0 | 1 | 0 | CHOS | 438.228741 | SB | - |
| 437.239224 | 38 | 20 | 10 | 0 | 0 | 0 | CHO | 438.2465 | BB | - |
| 437.254479 | 38 | 24 | 7 | 0 | 0 | 0 | CHO | 438.261755 | BB | 1 |
| 438.039496 | 17 | 12 | 9 | 5 | 2 | 0 | CHNOS | 439.046772 | SB | - |
| 438.05138 | 13 | 19 | 6 | 5 | 1 | 0 | CHNOS | 439.058656 | SB | 1 |
| 438.075881 | 21 | 13 | 8 | 5 | 2 | 0 | CHNOS | 439.083157 | SB | - |
| 438.104188 | 21 | 19 | 11 | 1 | 0 | 0 | CHNO | 439.111464 | SB | - |
| 438.116757 | 17 | 19 | 6 | 7 | 0 | 0 | CHNO | 439.124033 | BB | - |
| 438.161703 | 29 | 17 | 12 | 1 | 0 | 0 | CHNO | 439.168979 | SB | - |
| 439.051819 | 16 | 18 | 13 | 0 | 0 | 0 | CHO | 440.059095 | SB | - |
| 439.07632 | 24 | 12 | 15 | 0 | 1 | 0 | CHOS | 440.083596 | SB | - |
| 439.0857 | 20 | 23 | 7 | 0 | 1 | 0 | CHOS | 440.092976 | SB | - |
| 439.088204 | 20 | 19 | 12 | 0 | 0 | 0 | CHO | 440.09548 | SB | - |
| 439.109334 | 24 | 16 | 14 | 0 | 0 | 0 | CHO | 440.11661 | SB | - |
| 439.160974 | 28 | 21 | 10 | 0 | 0 | 0 | CHO | 440.16825 | BB | - |
| 439.182104 | 32 | 18 | 12 | 0 | 0 | 0 | CHO | 440.18938 | BB | - |
| 439.218489 | 36 | 19 | 11 | 0 | 0 | 0 | CHO | 440.225765 | BB | - |
| 439.254874 | 40 | 20 | 10 | 0 | 0 | 0 | CHO | 440.26215 | BB | - |
| 439.379284 | 52 | 27 | 4 | 0 | 0 | 0 | CHO | 440.38656 | BB | 2 |
| 440.055146 | 19 | 12 | 9 | 5 | 2 | 0 | CHNOS | 441.062422 | SB | - |
| 440.089027 | 23 | 17 | 3 | 5 | 3 | 0 | CHNOS | 441.096303 | SB | - |
| 440.091531 | 23 | 13 | 8 | 5 | 2 | 0 | CHNOS | 441.098807 | SB | - |
| 440.112661 | 27 | 10 | 10 | 5 | 2 | 0 | CHNOS | 441.119937 | SB | - |
| 440.140968 | 27 | 16 | 13 | 1 | 0 | 0 | CHNO | 441.148244 | SB | - |
| 440.164301 | 31 | 15 | 6 | 5 | 2 | 0 | CHNOS | 441.171577 | BB | - |
| 441.031084 | 14 | 17 | 14 | 0 | 0 | 0 | CHO | 442.03836 | SB | - |
| 441.067469 | 18 | 18 | 13 | 0 | 0 | 0 | CHO | 442.074745 | SB | - |
| 441.072176 | 18 | 16 | 9 | 4 | 1 | 0 | CHNOS | 442.079452 | SB | - |
| 441.09197 | 26 | 12 | 15 | 0 | 1 | 0 | CHOS | 442.099246 | SB | - |
| 441.103854 | 22 | 19 | 12 | 0 | 0 | 0 | CHO | 442.11113 | SB | - |
| 441.119109 | 22 | 23 | 9 | 0 | 0 | 0 | CHO | 442.126385 | BB | 3 |
| 441.249394 | 38 | 23 | 8 | 0 | 0 | 0 | CHO | 442.25667 | BB | - |
| 442.064921 | 17 | 19 | 4 | 5 | 2 | 0 | CHNOS | 443.072197 | SB | - |
| 442.070796 | 21 | 12 | 9 | 5 | 2 | 0 | CHNOS | 443.078072 | SB | - |
| 442.101306 | 21 | 20 | 3 | 5 | 2 | 0 | CHNOS | 443.108582 | BB | - |
| 442.107181 | 25 | 13 | 8 | 5 | 2 | 0 | CHNOS | 443.114457 | SB | - |
| 442.989435 | 16 | 12 | 10 | 2 | 3 | 0 | CHNOS | 443.996711 | SB | - |
| 443.02582 | 20 | 13 | 9 | 2 | 3 | 0 | CHNOS | 444.033096 | SB | - |
| 443.046734 | 16 | 17 | 14 | 0 | 0 | 0 | CHO | 444.05401 | SB | - |
| 443.061989 | 16 | 21 | 11 | 0 | 0 | 0 | CHO | 444.069265 | SB | - |
| 443.083119 | 20 | 18 | 13 | 0 | 0 | 0 | CHO | 444.090395 | SB | - |
| 443.101745 | 24 | 19 | 10 | 0 | 1 | 0 | CHOS | 444.109021 | SB | - |
| 443.134759 | 24 | 23 | 9 | 0 | 0 | 0 | CHO | 444.142035 | BB | 7 |
| 443.195645 | 36 | 18 | 10 | 0 | 1 | 0 | CHOS | 444.202921 | SB | - |
| 443.228659 | 36 | 22 | 9 | 0 | 0 | 0 | CHO | 444.235935 | BB | - |
| 443.265044 | 40 | 23 | 8 | 0 | 0 | 0 | CHO | 444.27232 | BB | 2 |
| 444.028931 | 15 | 14 | 8 | 5 | 2 | 0 | CHNOS | 445.036207 | SB | - |
| 444.061945 | 15 | 18 | 7 | 5 | 1 | 0 | CHNOS | 445.069221 | SB | - |
| 444.080102 | 27 | 19 | 3 | 1 | 4 | 0 | CHNOS | 445.087378 | SB | - |
| 444.086446 | 23 | 12 | 9 | 5 | 2 | 0 | CHNOS | 445.093722 | SB | - |
| 444.101701 | 23 | 16 | 6 | 5 | 2 | 0 | CHNOS | 445.108977 | BB | - |
| 445.005085 | 18 | 12 | 10 | 2 | 3 | 0 | CHNOS | 446.012361 | SB | 4 |
| 445.041254 | 14 | 20 | 12 | 0 | 0 | 0 | CHO | 446.04853 | SB | - |
| 445.062384 | 18 | 17 | 14 | 0 | 0 | 0 | CHO | 446.06966 | SB | - |
| 445.098769 | 22 | 18 | 13 | 0 | 0 | 0 | CHO | 446.106045 | SB | - |
| 445.150409 | 26 | 23 | 9 | 0 | 0 | 0 | CHO | 446.157685 | BB | 3 |
| 445.171539 | 30 | 20 | 11 | 0 | 0 | 0 | CHO | 446.178815 | BB | - |
| 445.280694 | 42 | 23 | 8 | 0 | 0 | 0 | CHO | 446.28797 | BB | - |
| 445.317079 | 46 | 24 | 7 | 0 | 0 | 0 | CHO | 446.324355 | BB | - |
| 446.030844 | 17 | 19 | 4 | 3 | 3 | 0 | CHNOS | 447.03812 | SB | - |
| 446.044581 | 17 | 14 | 8 | 5 | 2 | 0 | CHNOS | 447.051857 | SB | - |
| 446.067229 | 21 | 20 | 3 | 3 | 3 | 0 | CHNOS | 447.074505 | SB | - |
| 446.11398 | 21 | 19 | 6 | 5 | 1 | 0 | CHNOS | 447.121256 | BB | - |
| 446.117351 | 25 | 16 | 6 | 5 | 2 | 0 | CHNOS | 447.124627 | BB | - |
| 447.020519 | 12 | 19 | 13 | 0 | 0 | 0 | CHO | 448.027795 | SB | - |
| 447.078034 | 20 | 17 | 14 | 0 | 0 | 0 | CHO | 448.08531 | SB | - |
| 447.129674 | 24 | 22 | 10 | 0 | 0 | 0 | CHO | 448.13695 | BB | 23 |
| 447.166059 | 28 | 23 | 9 | 0 | 0 | 0 | CHO | 448.173335 | BB | 2 |
| 448.023846 | 15 | 13 | 9 | 5 | 2 | 0 | CHNOS | 449.031122 | SB | - |
| 448.075486 | 19 | 18 | 5 | 5 | 2 | 0 | CHNOS | 449.082762 | SB | - |
| 448.088538 | 19 | 20 | 11 | 1 | 0 | 0 | CHNO | 449.095814 | SB | - |
| 448.124923 | 23 | 21 | 10 | 1 | 0 | 0 | CHNO | 449.132199 | SB | - |
| 448.133001 | 27 | 16 | 6 | 5 | 2 | 0 | CHNOS | 449.140277 | BB | 22 |
| 449.036169 | 14 | 19 | 13 | 0 | 0 | 0 | CHO | 450.043445 | SB | - |
| 449.051424 | 14 | 23 | 10 | 0 | 0 | 0 | CHO | 450.0587 | BB | - |
| 449.072554 | 18 | 20 | 12 | 0 | 0 | 0 | CHO | 450.07983 | SB | 5 |
| 449.093684 | 22 | 17 | 14 | 0 | 0 | 0 | CHO | 450.10096 | SB | - |
| 449.097055 | 26 | 14 | 14 | 0 | 1 | 0 | CHOS | 450.104331 | SB | - |
| 450.039496 | 17 | 13 | 9 | 5 | 2 | 0 | CHNOS | 451.046772 | SB | - |
| 450.062144 | 21 | 19 | 4 | 3 | 3 | 0 | CHNOS | 451.06942 | SB | - |
| 450.075881 | 21 | 14 | 8 | 5 | 2 | 0 | CHNOS | 451.083157 | SB | 6 |
| 450.169781 | 33 | 13 | 8 | 5 | 2 | 0 | CHNOS | 451.177057 | BB | - |
| 451.030905 | 20 | 15 | 8 | 2 | 3 | 0 | CHNOS | 452.038181 | SB | - |
| 451.051819 | 16 | 19 | 13 | 0 | 0 | 0 | CHO | 452.059095 | SB | - |
| 451.069794 | 24 | 12 | 12 | 2 | 2 | 0 | CHNOS | 452.07707 | SB | 2 |
| 451.088204 | 20 | 20 | 12 | 0 | 0 | 0 | CHO | 452.09548 | SB | - |
| 451.160974 | 28 | 22 | 10 | 0 | 0 | 0 | CHO | 452.16825 | BB | - |
| 451.237115 | 40 | 21 | 8 | 0 | 1 | 0 | CHOS | 452.244391 | SB | - |
| 451.291259 | 44 | 22 | 9 | 0 | 0 | 0 | CHO | 452.298535 | SB | - |
| 452.083453 | 19 | 19 | 12 | 1 | 0 | 0 | CHNO | 453.090729 | SB | - |
| 452.091531 | 23 | 14 | 8 | 5 | 2 | 0 | CHNOS | 453.098807 | SB | - |
| 452.140968 | 27 | 17 | 13 | 1 | 0 | 0 | CHNO | 453.148244 | SB | - |
| 453.031084 | 14 | 18 | 14 | 0 | 0 | 0 | CHO | 454.03836 | SB | - |
| 453.056921 | 18 | 13 | 12 | 4 | 1 | 0 | CHNOS | 454.064197 | SB | - |
| 453.067469 | 18 | 19 | 13 | 0 | 0 | 0 | CHO | 454.074745 | SB | - |
| 453.082724 | 18 | 23 | 10 | 0 | 0 | 0 | CHO | 454.09 | BB | - |
| 453.103854 | 22 | 20 | 12 | 0 | 0 | 0 | CHO | 454.11113 | SB | - |
| 453.119109 | 22 | 24 | 9 | 0 | 0 | 0 | CHO | 454.126385 | BB | - |
| 453.21638 | 38 | 20 | 9 | 0 | 1 | 0 | CHOS | 454.223656 | SB | - |
| 453.301034 | 42 | 29 | 4 | 0 | 0 | 0 | CHO | 454.30831 | BB | 3 |
| 454.046295 | 13 | 19 | 7 | 5 | 1 | 0 | CHNOS | 455.053571 | SB | - |
| 454.070796 | 21 | 13 | 9 | 5 | 2 | 0 | CHNOS | 455.078072 | SB | - |
| 454.096599 | 21 | 23 | 7 | 1 | 1 | 0 | CHNOS | 455.103875 | SB | - |
| 454.135488 | 25 | 20 | 11 | 1 | 0 | 0 | CHNO | 455.142764 | SB | - |
| 454.989435 | 16 | 13 | 10 | 2 | 3 | 0 | CHNOS | 455.996711 | SB | - |
| 455.046734 | 16 | 18 | 14 | 0 | 0 | 0 | CHO | 456.05401 | SB | - |
| 455.072571 | 20 | 13 | 12 | 4 | 1 | 0 | CHNOS | 456.079847 | SB | - |
| 455.083119 | 20 | 19 | 13 | 0 | 0 | 0 | CHO | 456.090395 | SB | - |
| 455.098374 | 20 | 23 | 10 | 0 | 0 | 0 | CHO | 456.10565 | BB | 5 |
| 455.214271 | 40 | 20 | 7 | 0 | 2 | 0 | CHOS | 456.221547 | BB | - |
| 455.23203 | 40 | 20 | 9 | 0 | 1 | 0 | CHOS | 456.239306 | SB | - |
| 455.316684 | 44 | 29 | 4 | 0 | 0 | 0 | CHO | 456.32396 | BB | 10 |
| 456.086446 | 23 | 13 | 9 | 5 | 2 | 0 | CHNOS | 457.093722 | SB | - |
| 456.09833 | 19 | 20 | 6 | 5 | 1 | 0 | CHNOS | 457.105606 | BB | - |
| 456.320011 | 47 | 23 | 0 | 5 | 2 | 0 | CHNOS | 457.327287 | BB | 10 |
| 457.005085 | 18 | 13 | 10 | 2 | 3 | 0 | CHNOS | 458.012361 | SB | - |
| 457.041254 | 14 | 21 | 12 | 0 | 0 | 0 | CHO | 458.04853 | SB | - |
| 457.062384 | 18 | 18 | 14 | 0 | 0 | 0 | CHO | 458.06966 | SB | - |
| 457.086885 | 26 | 12 | 16 | 0 | 1 | 0 | CHOS | 458.094161 | SB | - |
| 457.098769 | 22 | 19 | 13 | 0 | 0 | 0 | CHO | 458.106045 | SB | - |
| 457.114024 | 22 | 23 | 10 | 0 | 0 | 0 | CHO | 458.1213 | BB | 3 |
| 457.144568 | 26 | 16 | 4 | 8 | 2 | 0 | CHNOS | 458.151844 | BB | - |
| 457.150409 | 26 | 24 | 9 | 0 | 0 | 0 | CHO | 458.157685 | BB | 1 |
| 457.192669 | 34 | 18 | 13 | 0 | 0 | 0 | CHO | 458.199945 | BB | - |
| 457.24768 | 42 | 20 | 9 | 0 | 1 | 0 | CHOS | 458.254956 | SB | 1 |
| 458.044581 | 17 | 15 | 8 | 5 | 2 | 0 | CHNOS | 459.051857 | SB | - |
| 458.059836 | 17 | 19 | 5 | 5 | 2 | 0 | CHNOS | 459.067112 | SB | - |
| 458.102096 | 25 | 13 | 9 | 5 | 2 | 0 | CHNOS | 459.109372 | SB | - |
| 458.174866 | 33 | 15 | 7 | 5 | 2 | 0 | CHNOS | 459.182142 | BB | - |
| 459.020735 | 20 | 13 | 10 | 2 | 3 | 0 | CHNOS | 460.028011 | SB | - |
| 459.056904 | 16 | 21 | 12 | 0 | 0 | 0 | CHO | 460.06418 | SB | - |
| 459.078034 | 20 | 18 | 14 | 0 | 0 | 0 | CHO | 460.08531 | SB | - |
| 459.223574 | 36 | 22 | 10 | 0 | 0 | 0 | CHO | 460.23085 | BB | - |
| 460.023846 | 15 | 14 | 9 | 5 | 2 | 0 | CHNOS | 461.031122 | SB | - |
| 460.046494 | 19 | 20 | 4 | 3 | 3 | 0 | CHNOS | 461.05377 | SB | - |
| 460.060231 | 19 | 15 | 8 | 5 | 2 | 0 | CHNOS | 461.067507 | SB | - |
| 460.075486 | 19 | 19 | 5 | 5 | 2 | 0 | CHNOS | 461.082762 | SB | - |
| 460.081361 | 23 | 12 | 10 | 5 | 2 | 0 | CHNOS | 461.088637 | SB | - |
| 460.088538 | 19 | 21 | 11 | 1 | 0 | 0 | CHNO | 461.095814 | SB | - |
| 460.093245 | 19 | 19 | 7 | 5 | 1 | 0 | CHNOS | 461.100521 | SB | - |
| 461.036169 | 14 | 20 | 13 | 0 | 0 | 0 | CHO | 462.043445 | SB | - |
| 461.058166 | 22 | 18 | 10 | 0 | 2 | 0 | CHOS | 462.065442 | BB | - |
| 461.072554 | 18 | 21 | 12 | 0 | 0 | 0 | CHO | 462.07983 | SB | 17 |
| 461.093684 | 22 | 18 | 14 | 0 | 0 | 0 | CHO | 462.10096 | SB | - |
| 461.145324 | 26 | 23 | 10 | 0 | 0 | 0 | CHO | 462.1526 | BB | 10 |
| 461.239224 | 38 | 22 | 10 | 0 | 0 | 0 | CHO | 462.2465 | BB | - |
| 461.275609 | 42 | 23 | 9 | 0 | 0 | 0 | CHO | 462.282885 | BB | - |
| 461.348379 | 50 | 25 | 7 | 0 | 0 | 0 | CHO | 462.355655 | BB | 1 |
| 462.039496 | 17 | 14 | 9 | 5 | 2 | 0 | CHNOS | 463.046772 | SB | - |
| 462.097011 | 25 | 12 | 10 | 5 | 2 | 0 | CHNOS | 463.104287 | SB | - |
| 462.104188 | 21 | 21 | 11 | 1 | 0 | 0 | CHNO | 463.111464 | SB | - |
| 462.116288 | 25 | 21 | 5 | 3 | 2 | 0 | CHNOS | 463.123564 | BB | - |
| 462.133396 | 29 | 13 | 9 | 5 | 2 | 0 | CHNOS | 463.140672 | BB | - |
| 463.051819 | 16 | 20 | 13 | 0 | 0 | 0 | CHO | 464.059095 | SB | - |
| 463.088204 | 20 | 21 | 12 | 0 | 0 | 0 | CHO | 464.09548 | SB | 2 |
| 463.130632 | 20 | 21 | 3 | 8 | 1 | 0 | CHNOS | 464.137908 | SB | - |
| 463.160974 | 28 | 23 | 10 | 0 | 0 | 0 | CHO | 464.16825 | BB | 7 |
| 463.254874 | 40 | 22 | 10 | 0 | 0 | 0 | CHO | 464.26215 | BB | - |
| 464.055146 | 19 | 14 | 9 | 5 | 2 | 0 | CHNOS | 465.062422 | SB | - |
| 464.070401 | 19 | 18 | 6 | 5 | 2 | 0 | CHNOS | 465.077677 | BB | - |
| 464.077794 | 23 | 20 | 4 | 3 | 3 | 0 | CHNOS | 465.08507 | SB | - |
| 464.091531 | 23 | 15 | 8 | 5 | 2 | 0 | CHNOS | 465.098807 | SB | 52 |
| 464.119838 | 23 | 21 | 11 | 1 | 0 | 0 | CHNO | 465.127114 | SB | - |
| 465.031084 | 14 | 19 | 14 | 0 | 0 | 0 | CHO | 466.03836 | SB | - |
| 465.067469 | 18 | 20 | 13 | 0 | 0 | 0 | CHO | 466.074745 | SB | - |
| 465.082724 | 18 | 24 | 10 | 0 | 0 | 0 | CHO | 466.09 | BB | - |
| 465.103854 | 22 | 21 | 12 | 0 | 0 | 0 | CHO | 466.11113 | SB | 6 |
| 465.119109 | 22 | 25 | 9 | 0 | 0 | 0 | CHO | 466.126385 | BB | 7 |
| 465.176624 | 30 | 23 | 10 | 0 | 0 | 0 | CHO | 466.1839 | BB | - |
| 465.234139 | 38 | 21 | 11 | 0 | 0 | 0 | CHO | 466.241415 | BB | - |
| 466.057059 | 21 | 19 | 5 | 3 | 3 | 0 | CHNOS | 467.064335 | SB | - |
| 466.070796 | 21 | 14 | 9 | 5 | 2 | 0 | CHNOS | 467.078072 | SB | - |
| 466.074818 | 21 | 19 | 7 | 3 | 2 | 0 | CHNOS | 467.082094 | SB | - |
| 466.099103 | 21 | 20 | 12 | 1 | 0 | 0 | CHNO | 467.106379 | SB | - |
| 467.046734 | 16 | 19 | 14 | 0 | 0 | 0 | CHO | 468.05401 | SB | - |
| 467.083119 | 20 | 20 | 13 | 0 | 0 | 0 | CHO | 468.090395 | SB | - |
| 467.10762 | 28 | 14 | 15 | 0 | 1 | 0 | CHOS | 468.114896 | SB | - |
| 467.119504 | 24 | 21 | 12 | 0 | 0 | 0 | CHO | 468.12678 | SB | 4 |
| 467.213404 | 36 | 20 | 12 | 0 | 0 | 0 | CHO | 468.22068 | BB | - |
| 468.044186 | 15 | 20 | 5 | 5 | 2 | 0 | CHNOS | 469.051462 | SB | - |
| 468.050061 | 19 | 13 | 10 | 5 | 2 | 0 | CHNOS | 469.057337 | SB | - |
| 468.072709 | 23 | 19 | 5 | 3 | 3 | 0 | CHNOS | 469.079985 | SB | - |
| 468.086446 | 23 | 14 | 9 | 5 | 2 | 0 | CHNOS | 469.093722 | SB | - |
| 468.143961 | 31 | 12 | 10 | 5 | 2 | 0 | CHNOS | 469.151237 | BB | - |
| 469.062384 | 18 | 19 | 14 | 0 | 0 | 0 | CHO | 470.06966 | SB | - |
| 469.098769 | 22 | 20 | 13 | 0 | 0 | 0 | CHO | 470.106045 | SB | - |
| 469.114024 | 22 | 24 | 10 | 0 | 0 | 0 | CHO | 470.1213 | BB | 2 |
| 469.150409 | 26 | 25 | 9 | 0 | 0 | 0 | CHO | 470.157685 | BB | 1 |
| 470.065711 | 21 | 13 | 10 | 5 | 2 | 0 | CHNOS | 471.072987 | SB | - |
| 470.077595 | 17 | 20 | 7 | 5 | 1 | 0 | CHNOS | 471.084871 | SB | - |
| 470.102096 | 25 | 14 | 9 | 5 | 2 | 0 | CHNOS | 471.109372 | SB | - |
| 470.151533 | 29 | 17 | 14 | 1 | 0 | 0 | CHNO | 471.158809 | SB | 1 |
| 470.174866 | 33 | 16 | 7 | 5 | 2 | 0 | CHNOS | 471.182142 | BB | - |
| 471.020735 | 20 | 14 | 10 | 2 | 3 | 0 | CHNOS | 472.028011 | SB | - |
| 471.041649 | 16 | 18 | 15 | 0 | 0 | 0 | CHO | 472.048925 | SB | - |
| 471.056904 | 16 | 22 | 12 | 0 | 0 | 0 | CHO | 472.06418 | SB | - |
| 471.067486 | 20 | 13 | 13 | 4 | 1 | 0 | CHNOS | 472.074762 | SB | - |
| 471.078034 | 20 | 19 | 14 | 0 | 0 | 0 | CHO | 472.08531 | SB | - |
| 471.129674 | 24 | 24 | 10 | 0 | 0 | 0 | CHO | 472.13695 | BB | 1 |
| 471.187189 | 32 | 22 | 11 | 0 | 0 | 0 | CHO | 472.194465 | BB | - |
| 471.226945 | 40 | 20 | 10 | 0 | 1 | 0 | CHOS | 472.234221 | SB | - |
| 472.023846 | 15 | 15 | 9 | 5 | 2 | 0 | CHNOS | 473.031122 | SB | - |
| 472.05686 | 15 | 19 | 8 | 5 | 1 | 0 | CHNOS | 473.064136 | SB | - |
| 472.060231 | 19 | 16 | 8 | 5 | 2 | 0 | CHNOS | 473.067507 | SB | - |
| 472.081361 | 23 | 13 | 10 | 5 | 2 | 0 | CHNOS | 473.088637 | SB | - |
| 472.111871 | 23 | 21 | 4 | 5 | 2 | 0 | CHNOS | 473.119147 | BB | - |
| 472.133001 | 27 | 18 | 6 | 5 | 2 | 0 | CHNOS | 473.140277 | SB | 1 |
| 472.154131 | 31 | 15 | 8 | 5 | 2 | 0 | CHNOS | 473.161407 | BB | - |
| 473 | 18 | 13 | 11 | 2 | 3 | 0 | CHNOS | 474.007276 | SB | - |
| 473.036169 | 14 | 21 | 13 | 0 | 0 | 0 | CHO | 474.043445 | SB | - |
| 473.057299 | 18 | 18 | 15 | 0 | 0 | 0 | CHO | 474.064575 | SB | - |
| 473.072554 | 18 | 22 | 12 | 0 | 0 | 0 | CHO | 474.07983 | SB | 2 |
| 473.083136 | 22 | 13 | 13 | 4 | 1 | 0 | CHNOS | 474.090412 | SB | - |
| 473.093684 | 22 | 19 | 14 | 0 | 0 | 0 | CHO | 474.10096 | SB | - |
| 473.145324 | 26 | 24 | 10 | 0 | 0 | 0 | CHO | 474.1526 | BB | 1 |
| 473.187584 | 34 | 18 | 14 | 0 | 0 | 0 | CHO | 474.19486 | SB | - |
| 474.039496 | 17 | 15 | 9 | 5 | 2 | 0 | CHNOS | 475.046772 | SB | - |
| 474.091136 | 21 | 20 | 5 | 5 | 2 | 0 | CHNOS | 475.098412 | SB | - |
| 474.097011 | 25 | 13 | 10 | 5 | 2 | 0 | CHNOS | 475.104287 | SB | - |
| 474.104188 | 21 | 22 | 11 | 1 | 0 | 0 | CHNO | 475.111464 | SB | - |
| 474.148651 | 29 | 18 | 6 | 5 | 2 | 0 | CHNOS | 475.155927 | BB | 2 |
| 474.169781 | 33 | 15 | 8 | 5 | 2 | 0 | CHNOS | 475.177057 | SB | - |
| 475.01565 | 20 | 13 | 11 | 2 | 3 | 0 | CHNOS | 476.022926 | SB | - |
| 475.051819 | 16 | 21 | 13 | 0 | 0 | 0 | CHO | 476.059095 | SB | - |
| 475.088204 | 20 | 22 | 12 | 0 | 0 | 0 | CHO | 476.09548 | SB | 17 |
| 475.130163 | 28 | 22 | 2 | 4 | 3 | 0 | CHNOS | 476.137439 | SB | - |
| 475.160974 | 28 | 24 | 10 | 0 | 0 | 0 | CHO | 476.16825 | BB | 1 |
| 475.20073 | 36 | 22 | 9 | 0 | 1 | 0 | CHOS | 476.208006 | SB | - |
| 476.041409 | 19 | 20 | 5 | 3 | 3 | 0 | CHNOS | 477.048685 | SB | - |
| 476.055146 | 19 | 15 | 9 | 5 | 2 | 0 | CHNOS | 477.062422 | SB | - |
| 476.077794 | 23 | 21 | 4 | 3 | 3 | 0 | CHNOS | 477.08507 | SB | - |
| 476.091531 | 23 | 16 | 8 | 5 | 2 | 0 | CHNOS | 477.098807 | SB | 16 |
| 476.119838 | 23 | 22 | 11 | 1 | 0 | 0 | CHNO | 477.127114 | SB | 3 |
| 477.031084 | 14 | 20 | 14 | 0 | 0 | 0 | CHO | 478.03836 | SB | - |
| 477.067469 | 18 | 21 | 13 | 0 | 0 | 0 | CHO | 478.074745 | SB | 10 |
| 477.103854 | 22 | 22 | 12 | 0 | 0 | 0 | CHO | 478.11113 | SB | 46 |
| 477.140239 | 26 | 23 | 11 | 0 | 0 | 0 | CHO | 478.147515 | BB | 9 |
| 477.21638 | 38 | 22 | 9 | 0 | 1 | 0 | CHOS | 478.223656 | SB | - |
| 477.270524 | 42 | 23 | 10 | 0 | 0 | 0 | CHO | 478.2778 | BB | - |
| 478.034411 | 17 | 14 | 10 | 5 | 2 | 0 | CHNOS | 479.041687 | SB | - |
| 478.038433 | 17 | 19 | 8 | 3 | 2 | 0 | CHNOS | 479.045709 | SB | - |
| 478.057059 | 21 | 20 | 5 | 3 | 3 | 0 | CHNOS | 479.064335 | SB | - |
| 478.070796 | 21 | 15 | 9 | 5 | 2 | 0 | CHNOS | 479.078072 | SB | 10 |
| 478.099103 | 21 | 21 | 12 | 1 | 0 | 0 | CHNO | 479.106379 | SB | - |
| 478.107181 | 25 | 16 | 8 | 5 | 2 | 0 | CHNOS | 479.114457 | SB | 46 |
| 478.219491 | 33 | 23 | 8 | 3 | 0 | 0 | CHNO | 479.226767 | SB | - |
| 479.02582 | 20 | 16 | 9 | 2 | 3 | 0 | CHNOS | 480.033096 | BB | - |
| 479.046734 | 16 | 20 | 14 | 0 | 0 | 0 | CHO | 480.05401 | SB | - |
| 479.056148 | 16 | 16 | 6 | 8 | 2 | 0 | CHNOS | 480.063424 | SB | - |
| 479.083119 | 20 | 21 | 13 | 0 | 0 | 0 | CHO | 480.090395 | SB | 18 |
| 479.249789 | 40 | 22 | 11 | 0 | 0 | 0 | CHO | 480.257065 | BB | - |
| 479.286174 | 44 | 23 | 10 | 0 | 0 | 0 | CHO | 480.29345 | BB | - |
| 480.050061 | 19 | 14 | 10 | 5 | 2 | 0 | CHNOS | 481.057337 | SB | - |
| 480.065316 | 19 | 18 | 7 | 5 | 2 | 0 | CHNOS | 481.072592 | BB | - |
| 480.071842 | 19 | 19 | 10 | 3 | 1 | 0 | CHNOS | 481.079118 | SB | - |
| 480.086446 | 23 | 15 | 9 | 5 | 2 | 0 | CHNOS | 481.093722 | SB | 18 |
| 480.109094 | 27 | 21 | 4 | 3 | 3 | 0 | CHNOS | 481.11637 | BB | 1 |
| 480.143961 | 31 | 13 | 10 | 5 | 2 | 0 | CHNOS | 481.151237 | BB | - |
| 481.025999 | 14 | 19 | 15 | 0 | 0 | 0 | CHO | 482.033275 | SB | - |
| 481.062384 | 18 | 20 | 14 | 0 | 0 | 0 | CHO | 482.06966 | SB | - |
| 481.098769 | 22 | 21 | 13 | 0 | 0 | 0 | CHO | 482.106045 | SB | 1 |
| 481.114024 | 22 | 25 | 10 | 0 | 0 | 0 | CHO | 482.1213 | BB | 3 |
| 482.059836 | 17 | 21 | 5 | 5 | 2 | 0 | CHNOS | 483.067112 | SB | - |
| 482.077595 | 17 | 21 | 7 | 5 | 1 | 0 | CHNOS | 483.084871 | SB | - |
| 482.088359 | 25 | 20 | 5 | 3 | 3 | 0 | CHNOS | 483.095635 | SB | - |
| 482.102096 | 25 | 15 | 9 | 5 | 2 | 0 | CHNOS | 483.109372 | SB | 1 |
| 482.117351 | 25 | 19 | 6 | 5 | 2 | 0 | CHNOS | 483.124627 | BB | 5 |
| 482.159611 | 33 | 13 | 10 | 5 | 2 | 0 | CHNOS | 483.166887 | BB | - |
| 482.174866 | 33 | 17 | 7 | 5 | 2 | 0 | CHNOS | 483.182142 | BB | - |
| 483.093289 | 20 | 24 | 11 | 0 | 0 | 0 | CHO | 484.100565 | BB | 2 |
| 483.166059 | 28 | 26 | 9 | 0 | 0 | 0 | CHO | 484.173335 | BB | - |
| 484.05686 | 15 | 20 | 8 | 5 | 1 | 0 | CHNOS | 485.064136 | SB | - |
| 484.111871 | 23 | 22 | 4 | 5 | 2 | 0 | CHNOS | 485.119147 | BB | - |
| 485 | 18 | 14 | 11 | 2 | 3 | 0 | CHNOS | 486.007276 | SB | - |
| 485.036169 | 14 | 22 | 13 | 0 | 0 | 0 | CHO | 486.043445 | SB | - |
| 485.057299 | 18 | 19 | 15 | 0 | 0 | 0 | CHO | 486.064575 | SB | - |
| 485.093684 | 22 | 20 | 14 | 0 | 0 | 0 | CHO | 486.10096 | SB | - |
| 485.166454 | 30 | 22 | 12 | 0 | 0 | 0 | CHO | 486.17373 | BB | - |
| 485.327249 | 46 | 30 | 5 | 0 | 0 | 0 | CHO | 486.334525 | SB | 3 |
| 486.054751 | 17 | 20 | 6 | 5 | 2 | 0 | CHNOS | 487.062027 | SB | - |
| 486.060626 | 21 | 13 | 11 | 5 | 2 | 0 | CHNOS | 487.067902 | SB | - |
| 487.051819 | 16 | 22 | 13 | 0 | 0 | 0 | CHO | 488.059095 | SB | - |
| 487.072949 | 20 | 19 | 15 | 0 | 0 | 0 | CHO | 488.080225 | SB | - |
| 487.160974 | 28 | 25 | 10 | 0 | 0 | 0 | CHO | 488.16825 | BB | - |
| 488.091531 | 23 | 17 | 8 | 5 | 2 | 0 | CHNOS | 489.098807 | SB | - |
| 489.031084 | 14 | 21 | 14 | 0 | 0 | 0 | CHO | 490.03836 | SB | - |
| 489.049059 | 22 | 14 | 13 | 2 | 2 | 0 | CHNOS | 490.056335 | SB | - |
| 489.067469 | 18 | 22 | 13 | 0 | 0 | 0 | CHO | 490.074745 | SB | - |
| 489.088599 | 22 | 19 | 15 | 0 | 0 | 0 | CHO | 490.095875 | SB | - |
| 489.129691 | 26 | 18 | 10 | 4 | 1 | 0 | CHNOS | 490.136967 | SB | - |
| 489.140239 | 26 | 24 | 11 | 0 | 0 | 0 | CHO | 490.147515 | BB | 11 |
| 489.176624 | 30 | 25 | 10 | 0 | 0 | 0 | CHO | 490.1839 | BB | 1 |
| 489.182499 | 34 | 18 | 15 | 0 | 0 | 0 | CHO | 490.189775 | SB | - |
| 489.21638 | 38 | 23 | 9 | 0 | 1 | 0 | CHOS | 490.223656 | SB | - |
| 490.034411 | 17 | 15 | 10 | 5 | 2 | 0 | CHNOS | 491.041687 | SB | - |
| 490.070796 | 21 | 16 | 9 | 5 | 2 | 0 | CHNOS | 491.078072 | SB | - |
| 490.099103 | 21 | 22 | 12 | 1 | 0 | 0 | CHNO | 491.106379 | SB | - |
| 490.111203 | 25 | 22 | 6 | 3 | 2 | 0 | CHNOS | 491.118479 | BB | 5 |
| 490.137691 | 25 | 25 | 2 | 5 | 2 | 0 | CHNOS | 491.144967 | SB | 1 |
| 490.143566 | 29 | 18 | 7 | 5 | 2 | 0 | CHNOS | 491.150842 | BB | 10 |
| 490.219491 | 33 | 24 | 8 | 3 | 0 | 0 | CHNO | 491.226767 | SB | - |
| 491.046734 | 16 | 21 | 14 | 0 | 0 | 0 | CHO | 492.05401 | SB | - |
| 491.064709 | 24 | 14 | 13 | 2 | 2 | 0 | CHNOS | 492.071985 | SB | - |
| 491.083119 | 20 | 22 | 13 | 0 | 0 | 0 | CHO | 492.090395 | SB | 10 |
| 491.155889 | 28 | 24 | 11 | 0 | 0 | 0 | CHO | 492.163165 | BB | 3 |
| 491.161463 | 32 | 23 | 2 | 4 | 3 | 0 | CHNOS | 492.168739 | SB | - |
| 491.23203 | 40 | 23 | 9 | 0 | 1 | 0 | CHOS | 492.239306 | SB | - |
| 492.050061 | 19 | 15 | 10 | 5 | 2 | 0 | CHNOS | 493.057337 | SB | - |
| 492.086446 | 23 | 16 | 9 | 5 | 2 | 0 | CHNOS | 493.093722 | SB | 10 |
| 492.114753 | 23 | 22 | 12 | 1 | 0 | 0 | CHNO | 493.122029 | SB | - |
| 492.143961 | 31 | 14 | 10 | 5 | 2 | 0 | CHNOS | 493.151237 | BB | - |
| 492.159216 | 31 | 18 | 7 | 5 | 2 | 0 | CHNOS | 493.166492 | BB | 3 |
| 492.235141 | 35 | 24 | 8 | 3 | 0 | 0 | CHNO | 493.242417 | SB | - |
| 493.025999 | 14 | 20 | 15 | 0 | 0 | 0 | CHO | 494.033275 | SB | - |
| 493.04147 | 22 | 17 | 9 | 2 | 3 | 0 | CHNOS | 494.048746 | BB | - |
| 493.062384 | 18 | 21 | 14 | 0 | 0 | 0 | CHO | 494.06966 | SB | 4 |
| 493.098769 | 22 | 22 | 13 | 0 | 0 | 0 | CHO | 494.106045 | SB | 32 |
| 493.24768 | 42 | 23 | 9 | 0 | 1 | 0 | CHOS | 494.254956 | SB | - |
| 494.065711 | 21 | 15 | 10 | 5 | 2 | 0 | CHNOS | 495.072987 | SB | 4 |
| 494.088359 | 25 | 21 | 5 | 3 | 3 | 0 | CHNOS | 495.095635 | SB | - |
| 494.102096 | 25 | 16 | 9 | 5 | 2 | 0 | CHNOS | 495.109372 | SB | 31 |
| 494.159611 | 33 | 14 | 10 | 5 | 2 | 0 | CHNOS | 495.166887 | BB | - |
| 495.078034 | 20 | 21 | 14 | 0 | 0 | 0 | CHO | 496.08531 | SB | 2 |
| 495.244704 | 40 | 22 | 12 | 0 | 0 | 0 | CHO | 496.25198 | BB | - |
| 496.081361 | 23 | 15 | 10 | 5 | 2 | 0 | CHNOS | 497.088637 | SB | 2 |
| 496.175261 | 35 | 14 | 10 | 5 | 2 | 0 | CHNOS | 497.182537 | SB | 1 |
| 497.036169 | 14 | 23 | 13 | 0 | 0 | 0 | CHO | 498.043445 | SB | - |
| 497.057299 | 18 | 20 | 15 | 0 | 0 | 0 | CHO | 498.064575 | SB | - |
| 497.093684 | 22 | 21 | 14 | 0 | 0 | 0 | CHO | 498.10096 | SB | - |
| 497.108939 | 22 | 25 | 11 | 0 | 0 | 0 | CHO | 498.116215 | BB | - |
| 497.223969 | 38 | 21 | 13 | 0 | 0 | 0 | CHO | 498.231245 | BB | - |
| 498.07251 | 17 | 21 | 8 | 5 | 1 | 0 | CHNOS | 499.079786 | SB | - |
| 498.097011 | 25 | 15 | 10 | 5 | 2 | 0 | CHNOS | 499.104287 | SB | - |
| 498.294191 | 45 | 24 | 2 | 5 | 2 | 0 | CHNOS | 499.301467 | SB | - |
| 499.01565 | 20 | 15 | 11 | 2 | 3 | 0 | CHNOS | 500.022926 | SB | - |
| 499.051819 | 16 | 23 | 13 | 0 | 0 | 0 | CHO | 500.059095 | SB | - |
| 499.072949 | 20 | 20 | 15 | 0 | 0 | 0 | CHO | 500.080225 | SB | - |
| 499.109334 | 24 | 21 | 14 | 0 | 0 | 0 | CHO | 500.11661 | SB | - |
| 499.124589 | 24 | 25 | 11 | 0 | 0 | 0 | CHO | 500.131865 | BB | 4 |
| 499.12796 | 28 | 22 | 11 | 0 | 1 | 0 | CHOS | 500.135236 | SB | - |
| 500.08816 | 19 | 21 | 8 | 5 | 1 | 0 | CHNOS | 501.095436 | SB | - |
| 500.112661 | 27 | 15 | 10 | 5 | 2 | 0 | CHNOS | 501.119937 | SB | - |
| 500.127916 | 27 | 19 | 7 | 5 | 2 | 0 | CHNOS | 501.135192 | SB | 3 |
| 501.031084 | 14 | 22 | 14 | 0 | 0 | 0 | CHO | 502.03836 | SB | - |
| 501.067469 | 18 | 23 | 13 | 0 | 0 | 0 | CHO | 502.074745 | SB | - |
| 501.073043 | 22 | 22 | 4 | 4 | 3 | 0 | CHNOS | 502.080319 | SB | - |
| 501.088599 | 22 | 20 | 15 | 0 | 0 | 0 | CHO | 502.095875 | SB | - |
| 501.140239 | 26 | 25 | 11 | 0 | 0 | 0 | CHO | 502.147515 | BB | - |
| 501.176624 | 30 | 26 | 10 | 0 | 0 | 0 | CHO | 502.1839 | SB | 1 |
| 502.034411 | 17 | 16 | 10 | 5 | 2 | 0 | CHNOS | 503.041687 | SB | - |
| 502.070796 | 21 | 17 | 9 | 5 | 2 | 0 | CHNOS | 503.078072 | SB | - |
| 502.086051 | 21 | 21 | 6 | 5 | 2 | 0 | CHNOS | 503.093327 | SB | - |
| 502.091926 | 25 | 14 | 11 | 5 | 2 | 0 | CHNOS | 503.099202 | SB | - |
| 502.128311 | 29 | 15 | 10 | 5 | 2 | 0 | CHNOS | 503.135587 | SB | - |
| 503.046734 | 16 | 22 | 14 | 0 | 0 | 0 | CHO | 504.05401 | SB | - |
| 503.067864 | 20 | 19 | 16 | 0 | 0 | 0 | CHO | 504.07514 | SB | - |
| 503.083119 | 20 | 23 | 13 | 0 | 0 | 0 | CHO | 504.090395 | SB | 3 |
| 503.104249 | 24 | 20 | 15 | 0 | 0 | 0 | CHO | 504.111525 | SB | - |
| 503.155889 | 28 | 25 | 11 | 0 | 0 | 0 | CHO | 504.163165 | BB | 5 |
| 503.23203 | 40 | 24 | 9 | 0 | 1 | 0 | CHOS | 504.239306 | SB | - |
| 503.301429 | 44 | 29 | 7 | 0 | 0 | 0 | CHO | 504.308705 | SB | - |
| 504.050061 | 19 | 16 | 10 | 5 | 2 | 0 | CHNOS | 505.057337 | SB | - |
| 504.086446 | 23 | 17 | 9 | 5 | 2 | 0 | CHNOS | 505.093722 | SB | 3 |
| 505.025999 | 14 | 21 | 15 | 0 | 0 | 0 | CHO | 506.033275 | SB | - |
| 505.062384 | 18 | 22 | 14 | 0 | 0 | 0 | CHO | 506.06966 | SB | - |
| 505.098769 | 22 | 23 | 13 | 0 | 0 | 0 | CHO | 506.106045 | SB | 13 |
| 505.140728 | 30 | 23 | 3 | 4 | 3 | 0 | CHNOS | 506.148004 | SB | - |
| 505.171539 | 30 | 25 | 11 | 0 | 0 | 0 | CHO | 506.178815 | BB | 2 |
| 505.24768 | 42 | 24 | 9 | 0 | 1 | 0 | CHOS | 506.254956 | SB | - |
| 506.065711 | 21 | 16 | 10 | 5 | 2 | 0 | CHNOS | 507.072987 | SB | - |
| 506.102096 | 25 | 17 | 9 | 5 | 2 | 0 | CHNOS | 507.109372 | SB | 13 |
| 506.250791 | 37 | 25 | 8 | 3 | 0 | 0 | CHNO | 507.258067 | SB | - |
| 507.041649 | 16 | 21 | 15 | 0 | 0 | 0 | CHO | 508.048925 | SB | - |
| 507.056904 | 16 | 25 | 12 | 0 | 0 | 0 | CHO | 508.06418 | BB | 2 |
| 507.078034 | 20 | 22 | 14 | 0 | 0 | 0 | CHO | 508.08531 | SB | 1 |
| 507.093289 | 20 | 26 | 11 | 0 | 0 | 0 | CHO | 508.100565 | BB | - |
| 507.099164 | 24 | 19 | 16 | 0 | 0 | 0 | CHO | 508.10644 | SB | - |
| 507.114419 | 24 | 23 | 13 | 0 | 0 | 0 | CHO | 508.121695 | SB | 26 |
| 507.156847 | 24 | 23 | 4 | 8 | 1 | 0 | CHNOS | 508.164123 | BB | - |
| 507.242885 | 40 | 20 | 5 | 6 | 2 | 0 | CHNOS | 508.250161 | SB | - |
| 507.26333 | 44 | 24 | 9 | 0 | 1 | 0 | CHOS | 508.270606 | SB | - |
| 508.067624 | 23 | 21 | 6 | 3 | 3 | 0 | CHNOS | 509.0749 | SB | - |
| 508.081361 | 23 | 16 | 10 | 5 | 2 | 0 | CHNOS | 509.088637 | SB | 1 |
| 509.057299 | 18 | 21 | 15 | 0 | 0 | 0 | CHO | 510.064575 | SB | - |
| 509.093684 | 22 | 22 | 14 | 0 | 0 | 0 | CHO | 510.10096 | SB | - |
| 509.108939 | 22 | 26 | 11 | 0 | 0 | 0 | CHO | 510.116215 | BB | - |
| 509.145324 | 26 | 27 | 10 | 0 | 0 | 0 | CHO | 510.1526 | BB | - |
| 509.202839 | 34 | 25 | 11 | 0 | 0 | 0 | CHO | 510.210115 | BB | - |
| 510.075881 | 21 | 19 | 8 | 5 | 2 | 0 | CHNOS | 511.083157 | BB | - |
| 510.154526 | 33 | 14 | 11 | 5 | 2 | 0 | CHNOS | 511.161802 | BB | - |
| 511.01565 | 20 | 16 | 11 | 2 | 3 | 0 | CHNOS | 512.022926 | SB | - |
| 511.072949 | 20 | 21 | 15 | 0 | 0 | 0 | CHO | 512.080225 | SB | - |
| 511.09745 | 28 | 15 | 17 | 0 | 1 | 0 | CHOS | 512.104726 | SB | - |
| 511.109334 | 24 | 22 | 14 | 0 | 0 | 0 | CHO | 512.11661 | SB | - |
| 511.166849 | 32 | 20 | 15 | 0 | 0 | 0 | CHO | 512.174125 | BB | - |
| 511.312389 | 48 | 24 | 11 | 0 | 0 | 0 | CHO | 512.319665 | SB | - |
| 512.076276 | 23 | 15 | 11 | 5 | 2 | 0 | CHNOS | 513.083552 | SB | - |
| 512.112661 | 27 | 16 | 10 | 5 | 2 | 0 | CHNOS | 513.119937 | SB | - |
| 512.127916 | 27 | 20 | 7 | 5 | 2 | 0 | CHNOS | 513.135192 | BB | - |
| 512.185431 | 35 | 18 | 8 | 5 | 2 | 0 | CHNOS | 513.192707 | BB | - |
| 513.031084 | 14 | 23 | 14 | 0 | 0 | 0 | CHO | 514.03836 | SB | - |
| 513.067469 | 18 | 24 | 13 | 0 | 0 | 0 | CHO | 514.074745 | SB | - |
| 513.088599 | 22 | 21 | 15 | 0 | 0 | 0 | CHO | 514.095875 | SB | - |
| 514.107181 | 25 | 19 | 8 | 5 | 2 | 0 | CHNOS | 515.114457 | SB | - |
| 515.046734 | 16 | 23 | 14 | 0 | 0 | 0 | CHO | 516.05401 | SB | - |
| 515.067864 | 20 | 20 | 16 | 0 | 0 | 0 | CHO | 516.07514 | SB | - |
| 515.104249 | 24 | 21 | 15 | 0 | 0 | 0 | CHO | 516.111525 | SB | - |
| 515.155889 | 28 | 26 | 11 | 0 | 0 | 0 | CHO | 516.163165 | BB | 2 |
| 516.086446 | 23 | 18 | 9 | 5 | 2 | 0 | CHNOS | 517.093722 | SB | - |
| 517.025999 | 14 | 22 | 15 | 0 | 0 | 0 | CHO | 518.033275 | SB | - |
| 517.062384 | 18 | 23 | 14 | 0 | 0 | 0 | CHO | 518.06966 | SB | - |
| 517.083514 | 22 | 20 | 16 | 0 | 0 | 0 | CHO | 518.09079 | SB | - |
| 517.098769 | 22 | 24 | 13 | 0 | 0 | 0 | CHO | 518.106045 | SB | 9 |
| 517.119899 | 26 | 21 | 15 | 0 | 0 | 0 | CHO | 518.127175 | SB | - |
| 517.135154 | 26 | 25 | 12 | 0 | 0 | 0 | CHO | 518.14243 | BB | 4 |
| 517.171539 | 30 | 26 | 11 | 0 | 0 | 0 | CHO | 518.178815 | BB | 4 |
| 517.280694 | 42 | 29 | 8 | 0 | 0 | 0 | CHO | 518.28797 | SB | - |
| 518.123226 | 29 | 15 | 11 | 5 | 2 | 0 | CHNOS | 519.130502 | SB | - |
| 519.041649 | 16 | 22 | 15 | 0 | 0 | 0 | CHO | 520.048925 | SB | - |
| 519.078034 | 20 | 23 | 14 | 0 | 0 | 0 | CHO | 520.08531 | SB | 1 |
| 519.099164 | 24 | 20 | 16 | 0 | 0 | 0 | CHO | 520.10644 | SB | - |
| 519.150804 | 28 | 25 | 12 | 0 | 0 | 0 | CHO | 520.15808 | BB | 6 |
| 519.187189 | 32 | 26 | 11 | 0 | 0 | 0 | CHO | 520.194465 | BB | 3 |
| 519.26333 | 44 | 25 | 9 | 0 | 1 | 0 | CHOS | 520.270606 | SB | 1 |
| 520.081361 | 23 | 17 | 10 | 5 | 2 | 0 | CHNOS | 521.088637 | SB | 2 |
| 520.117746 | 27 | 18 | 9 | 5 | 2 | 0 | CHNOS | 521.125022 | SB | - |
| 521.057299 | 18 | 22 | 15 | 0 | 0 | 0 | CHO | 522.064575 | SB | - |
| 521.093684 | 22 | 23 | 14 | 0 | 0 | 0 | CHO | 522.10096 | SB | 4 |
| 521.114814 | 26 | 20 | 16 | 0 | 0 | 0 | CHO | 522.12209 | SB | - |
| 521.151199 | 30 | 21 | 15 | 0 | 0 | 0 | CHO | 522.158475 | BB | - |
| 521.166454 | 30 | 25 | 12 | 0 | 0 | 0 | CHO | 522.17373 | BB | 1 |
| 521.172497 | 26 | 24 | 4 | 8 | 1 | 0 | CHNOS | 522.179773 | SB | - |
| 521.202839 | 34 | 26 | 11 | 0 | 0 | 0 | CHO | 522.210115 | BB | 2 |
| 522.060626 | 21 | 16 | 11 | 5 | 2 | 0 | CHNOS | 523.067902 | SB | - |
| 523.052035 | 24 | 18 | 10 | 2 | 3 | 0 | CHNOS | 524.059311 | BB | - |
| 523.072949 | 20 | 22 | 15 | 0 | 0 | 0 | CHO | 524.080225 | SB | - |
| 523.109334 | 24 | 23 | 14 | 0 | 0 | 0 | CHO | 524.11661 | SB | 1 |
| 524.076276 | 23 | 16 | 11 | 5 | 2 | 0 | CHNOS | 525.083552 | SB | - |
| 525.030433 | 18 | 16 | 16 | 2 | 1 | 0 | CHNOS | 526.037709 | SB | - |
| 525.052214 | 18 | 21 | 16 | 0 | 0 | 0 | CHO | 526.05949 | SB | - |
| 525.088599 | 22 | 22 | 15 | 0 | 0 | 0 | CHO | 526.095875 | SB | - |
| 527.046734 | 16 | 24 | 14 | 0 | 0 | 0 | CHO | 528.05401 | SB | - |
| 527.067864 | 20 | 21 | 16 | 0 | 0 | 0 | CHO | 528.07514 | SB | - |
| 527.083119 | 20 | 25 | 13 | 0 | 0 | 0 | CHO | 528.090395 | SB | - |
| 527.104249 | 24 | 22 | 15 | 0 | 0 | 0 | CHO | 528.111525 | SB | - |
| 527.161764 | 32 | 20 | 16 | 0 | 0 | 0 | CHO | 528.16904 | BB | - |
| 528.107576 | 27 | 16 | 11 | 5 | 2 | 0 | CHNOS | 529.114852 | SB | - |
| 529.025999 | 14 | 23 | 15 | 0 | 0 | 0 | CHO | 530.033275 | SB | - |
| 529.062384 | 18 | 24 | 14 | 0 | 0 | 0 | CHO | 530.06966 | SB | - |
| 529.083514 | 22 | 21 | 16 | 0 | 0 | 0 | CHO | 530.09079 | SB | - |
| 529.104343 | 26 | 24 | 4 | 4 | 3 | 0 | CHNOS | 530.111619 | SB | - |
| 529.119899 | 26 | 22 | 15 | 0 | 0 | 0 | CHO | 530.127175 | SB | - |
| 529.171539 | 30 | 27 | 11 | 0 | 0 | 0 | CHO | 530.178815 | BB | 5 |
| 530.065711 | 21 | 18 | 10 | 5 | 2 | 0 | CHNOS | 531.072987 | SB | - |
| 530.123226 | 29 | 16 | 11 | 5 | 2 | 0 | CHNOS | 531.130502 | SB | - |
| 531.041649 | 16 | 23 | 15 | 0 | 0 | 0 | CHO | 532.048925 | SB | - |
| 531.078034 | 20 | 24 | 14 | 0 | 0 | 0 | CHO | 532.08531 | SB | - |
| 531.099164 | 24 | 21 | 16 | 0 | 0 | 0 | CHO | 532.10644 | SB | - |
| 532.081361 | 23 | 18 | 10 | 5 | 2 | 0 | CHNOS | 533.088637 | SB | - |
| 532.154131 | 31 | 20 | 8 | 5 | 2 | 0 | CHNOS | 533.161407 | SB | - |
| 533.057299 | 18 | 23 | 15 | 0 | 0 | 0 | CHO | 534.064575 | SB | - |
| 533.093684 | 22 | 24 | 14 | 0 | 0 | 0 | CHO | 534.10096 | SB | 10 |
| 533.172028 | 34 | 25 | 3 | 4 | 3 | 0 | CHNOS | 534.179304 | BB | - |
| 534.060626 | 21 | 17 | 11 | 5 | 2 | 0 | CHNOS | 535.067902 | SB | - |
| 534.083274 | 25 | 23 | 6 | 3 | 3 | 0 | CHNOS | 535.09055 | SB | - |
| 534.097011 | 25 | 18 | 10 | 5 | 2 | 0 | CHNOS | 535.104287 | SB | 10 |
| 534.133396 | 29 | 19 | 9 | 5 | 2 | 0 | CHNOS | 535.140672 | SB | - |
| 534.154526 | 33 | 16 | 11 | 5 | 2 | 0 | CHNOS | 535.161802 | SB | - |
| 535.072949 | 20 | 23 | 15 | 0 | 0 | 0 | CHO | 536.080225 | SB | - |
| 535.109334 | 24 | 24 | 14 | 0 | 0 | 0 | CHO | 536.11661 | SB | 4 |
| 535.151293 | 32 | 24 | 4 | 4 | 3 | 0 | CHNOS | 536.158569 | SB | 1 |
| 536.076276 | 23 | 17 | 11 | 5 | 2 | 0 | CHNOS | 537.083552 | SB | - |
| 537.052214 | 18 | 22 | 16 | 0 | 0 | 0 | CHO | 538.05949 | SB | - |
| 537.067469 | 18 | 26 | 13 | 0 | 0 | 0 | CHO | 538.074745 | BB | - |
| 537.088599 | 22 | 23 | 15 | 0 | 0 | 0 | CHO | 538.095875 | SB | - |
| 537.124984 | 26 | 24 | 14 | 0 | 0 | 0 | CHO | 538.13226 | SB | 4 |
| 538.164696 | 33 | 19 | 9 | 5 | 2 | 0 | CHNOS | 539.171972 | BB | - |
| 539.067864 | 20 | 22 | 16 | 0 | 0 | 0 | CHO | 540.07514 | SB | - |
| 539.104249 | 24 | 23 | 15 | 0 | 0 | 0 | CHO | 540.111525 | SB | - |
| 539.128918 | 24 | 23 | 4 | 8 | 2 | 0 | CHNOS | 540.136194 | SB | 7 |
| 539.177019 | 32 | 25 | 13 | 0 | 0 | 0 | CHO | 540.184295 | BB | 4 |
| 539.183062 | 28 | 24 | 5 | 8 | 1 | 0 | CHNOS | 540.190338 | SB | - |
| 540.107576 | 27 | 17 | 11 | 5 | 2 | 0 | CHNOS | 541.114852 | SB | - |
| 540.122831 | 27 | 21 | 8 | 5 | 2 | 0 | CHNOS | 541.130107 | BB | - |
| 541.026215 | 22 | 17 | 12 | 2 | 3 | 0 | CHNOS | 542.033491 | SB | - |
| 541.083514 | 22 | 22 | 16 | 0 | 0 | 0 | CHO | 542.09079 | SB | - |
| 541.119899 | 26 | 23 | 15 | 0 | 0 | 0 | CHO | 542.127175 | SB | - |
| 541.135154 | 26 | 27 | 12 | 0 | 0 | 0 | CHO | 542.14243 | BB | - |
| 541.177414 | 34 | 21 | 16 | 0 | 0 | 0 | CHO | 542.18469 | BB | - |
| 542.159611 | 33 | 18 | 10 | 5 | 2 | 0 | CHNOS | 543.166887 | BB | - |
| 543.041649 | 16 | 24 | 15 | 0 | 0 | 0 | CHO | 544.048925 | SB | - |
| 543.078034 | 20 | 25 | 14 | 0 | 0 | 0 | CHO | 544.08531 | SB | - |
| 543.099164 | 24 | 22 | 16 | 0 | 0 | 0 | CHO | 544.10644 | SB | - |
| 543.150804 | 28 | 27 | 12 | 0 | 0 | 0 | CHO | 544.15808 | BB | 1 |
| 544.081361 | 23 | 19 | 10 | 5 | 2 | 0 | CHNOS | 545.088637 | SB | - |
| 544.117746 | 27 | 20 | 9 | 5 | 2 | 0 | CHNOS | 545.125022 | SB | - |
| 544.154131 | 31 | 21 | 8 | 5 | 2 | 0 | CHNOS | 545.161407 | SB | 1 |
| 545.020914 | 14 | 23 | 16 | 0 | 0 | 0 | CHO | 546.02819 | SB | - |
| 545.057299 | 18 | 24 | 15 | 0 | 0 | 0 | CHO | 546.064575 | SB | - |
| 545.093684 | 22 | 25 | 14 | 0 | 0 | 0 | CHO | 546.10096 | SB | - |
| 545.114814 | 26 | 22 | 16 | 0 | 0 | 0 | CHO | 546.12209 | SB | - |
| 545.166454 | 30 | 27 | 12 | 0 | 0 | 0 | CHO | 546.17373 | BB | 2 |
| 546.097011 | 25 | 19 | 10 | 5 | 2 | 0 | CHNOS | 547.104287 | SB | - |
| 547.072949 | 20 | 24 | 15 | 0 | 0 | 0 | CHO | 548.080225 | SB | - |
| 547.109334 | 24 | 25 | 14 | 0 | 0 | 0 | CHO | 548.11661 | SB | 6 |
| 547.130464 | 28 | 22 | 16 | 0 | 0 | 0 | CHO | 548.13774 | SB | - |
| 547.291259 | 44 | 30 | 9 | 0 | 0 | 0 | CHO | 548.298535 | SB | 1 |
| 547.327644 | 48 | 31 | 8 | 0 | 0 | 0 | CHO | 548.33492 | SB | 2 |
| 548.076276 | 23 | 18 | 11 | 5 | 2 | 0 | CHNOS | 549.083552 | SB | - |
| 549.052214 | 18 | 23 | 16 | 0 | 0 | 0 | CHO | 550.05949 | SB | - |
| 549.088599 | 22 | 24 | 15 | 0 | 0 | 0 | CHO | 550.095875 | SB | 5 |
| 549.124984 | 26 | 25 | 14 | 0 | 0 | 0 | CHO | 550.13226 | SB | 6 |
| 549.166943 | 34 | 25 | 4 | 4 | 3 | 0 | CHNOS | 550.174219 | BB | - |
| 549.306909 | 46 | 30 | 9 | 0 | 0 | 0 | CHO | 550.314185 | SB | - |
| 550.091926 | 25 | 18 | 11 | 5 | 2 | 0 | CHNOS | 551.099202 | SB | 5 |
| 550.128311 | 29 | 19 | 10 | 5 | 2 | 0 | CHNOS | 551.135587 | SB | 6 |
| 551.067864 | 20 | 23 | 16 | 0 | 0 | 0 | CHO | 552.07514 | SB | - |
| 551.104249 | 24 | 24 | 15 | 0 | 0 | 0 | CHO | 552.111525 | SB | - |
| 551.213404 | 36 | 27 | 12 | 0 | 0 | 0 | CHO | 552.22068 | BB | - |
| 551.307304 | 48 | 26 | 12 | 0 | 0 | 0 | CHO | 552.31458 | SB | - |
| 552.107576 | 27 | 18 | 11 | 5 | 2 | 0 | CHNOS | 553.114852 | SB | - |
| 553.083514 | 22 | 23 | 16 | 0 | 0 | 0 | CHO | 554.09079 | SB | - |
| 553.099454 | 22 | 20 | 11 | 6 | 1 | 0 | CHNOS | 554.10673 | SB | - |
| 553.119899 | 26 | 24 | 15 | 0 | 0 | 0 | CHO | 554.127175 | SB | - |
| 553.177414 | 34 | 22 | 16 | 0 | 0 | 0 | CHO | 554.18469 | BB | - |
| 553.322954 | 50 | 26 | 12 | 0 | 0 | 0 | CHO | 554.33023 | SB | - |
| 554.123226 | 29 | 18 | 11 | 5 | 2 | 0 | CHNOS | 555.130502 | SB | - |
| 554.159611 | 33 | 19 | 10 | 5 | 2 | 0 | CHNOS | 555.166887 | SB | - |
| 555.041649 | 16 | 25 | 15 | 0 | 0 | 0 | CHO | 556.048925 | SB | - |
| 555.062779 | 20 | 22 | 17 | 0 | 0 | 0 | CHO | 556.070055 | SB | - |
| 555.099164 | 24 | 23 | 16 | 0 | 0 | 0 | CHO | 556.10644 | SB | - |
| 555.150804 | 28 | 28 | 12 | 0 | 0 | 0 | CHO | 556.15808 | SB | 1 |
| 555.156679 | 32 | 21 | 17 | 0 | 0 | 0 | CHO | 556.163955 | BB | - |
| 556.102491 | 27 | 17 | 12 | 5 | 2 | 0 | CHNOS | 557.109767 | SB | - |
| 557.057299 | 18 | 25 | 15 | 0 | 0 | 0 | CHO | 558.064575 | SB | - |
| 557.078429 | 22 | 22 | 17 | 0 | 0 | 0 | CHO | 558.085705 | SB | - |
| 557.114814 | 26 | 23 | 16 | 0 | 0 | 0 | CHO | 558.12209 | SB | - |
| 557.135643 | 30 | 26 | 4 | 4 | 3 | 0 | CHNOS | 558.142919 | SB | - |
| 557.172329 | 34 | 21 | 17 | 0 | 0 | 0 | CHO | 558.179605 | BB | - |
| 558.097011 | 25 | 20 | 10 | 5 | 2 | 0 | CHNOS | 559.104287 | SB | - |
| 559.072949 | 20 | 25 | 15 | 0 | 0 | 0 | CHO | 560.080225 | SB | - |
| 559.094079 | 24 | 22 | 17 | 0 | 0 | 0 | CHO | 560.101355 | SB | - |
| 559.109334 | 24 | 26 | 14 | 0 | 0 | 0 | CHO | 560.11661 | SB | 1 |
| 559.130464 | 28 | 23 | 16 | 0 | 0 | 0 | CHO | 560.13774 | SB | - |
| 559.182104 | 32 | 28 | 12 | 0 | 0 | 0 | CHO | 560.18938 | BB | - |
| 560.112661 | 27 | 20 | 10 | 5 | 2 | 0 | CHNOS | 561.119937 | SB | 1 |
| 560.149046 | 31 | 21 | 9 | 5 | 2 | 0 | CHNOS | 561.156322 | SB | - |
| 561.088599 | 22 | 25 | 15 | 0 | 0 | 0 | CHO | 562.095875 | SB | - |
| 561.124984 | 26 | 26 | 14 | 0 | 0 | 0 | CHO | 562.13226 | SB | - |
| 562.091926 | 25 | 19 | 11 | 5 | 2 | 0 | CHNOS | 563.099202 | SB | - |
| 563.067864 | 20 | 24 | 16 | 0 | 0 | 0 | CHO | 564.07514 | SB | - |
| 563.104249 | 24 | 25 | 15 | 0 | 0 | 0 | CHO | 564.111525 | SB | 2 |
| 564.107576 | 27 | 19 | 11 | 5 | 2 | 0 | CHNOS | 565.114852 | SB | 2 |
| 565.047129 | 18 | 23 | 17 | 0 | 0 | 0 | CHO | 566.054405 | SB | - |
| 565.083514 | 22 | 24 | 16 | 0 | 0 | 0 | CHO | 566.09079 | SB | - |
| 565.119899 | 26 | 25 | 15 | 0 | 0 | 0 | CHO | 566.127175 | SB | 8 |
| 566.123226 | 29 | 19 | 11 | 5 | 2 | 0 | CHNOS | 567.130502 | SB | 8 |
| 567.062779 | 20 | 23 | 17 | 0 | 0 | 0 | CHO | 568.070055 | SB | - |
| 567.07825 | 28 | 20 | 11 | 2 | 3 | 0 | CHNOS | 568.085526 | BB | - |
| 567.078719 | 20 | 20 | 12 | 6 | 1 | 0 | CHNOS | 568.085995 | BB | - |
| 567.099164 | 24 | 24 | 16 | 0 | 0 | 0 | CHO | 568.10644 | SB | - |
| 567.114419 | 24 | 28 | 13 | 0 | 0 | 0 | CHO | 568.121695 | BB | 3 |
| 567.135549 | 28 | 25 | 15 | 0 | 0 | 0 | CHO | 568.142825 | SB | 1 |
| 567.156679 | 32 | 22 | 17 | 0 | 0 | 0 | CHO | 568.163955 | BB | - |
| 567.208319 | 36 | 27 | 13 | 0 | 0 | 0 | CHO | 568.215595 | BB | - |
| 568.102491 | 27 | 18 | 12 | 5 | 2 | 0 | CHNOS | 569.109767 | SB | - |
| 568.138876 | 31 | 19 | 11 | 5 | 2 | 0 | CHNOS | 569.146152 | SB | 1 |
| 569.077946 | 18 | 21 | 6 | 10 | 2 | 0 | CHNOS | 570.085222 | SB | - |
| 569.078429 | 22 | 23 | 17 | 0 | 0 | 0 | CHO | 570.085705 | SB | - |
| 569.114814 | 26 | 24 | 16 | 0 | 0 | 0 | CHO | 570.12209 | SB | - |
| 569.172329 | 34 | 22 | 17 | 0 | 0 | 0 | CHO | 570.179605 | BB | - |
| 570.118141 | 29 | 18 | 12 | 5 | 2 | 0 | CHNOS | 571.125417 | SB | - |
| 570.155862 | 29 | 20 | 7 | 9 | 2 | 0 | CHNOS | 571.163138 | SB | - |
| 571.036564 | 16 | 25 | 16 | 0 | 0 | 0 | CHO | 572.04384 | SB | - |
| 571.072949 | 20 | 26 | 15 | 0 | 0 | 0 | CHO | 572.080225 | SB | - |
| 571.094079 | 24 | 23 | 17 | 0 | 0 | 0 | CHO | 572.101355 | SB | - |
| 571.130464 | 28 | 24 | 16 | 0 | 0 | 0 | CHO | 572.13774 | SB | - |
| 571.187979 | 36 | 22 | 17 | 0 | 0 | 0 | CHO | 572.195255 | BB | - |
| 573.052214 | 18 | 25 | 16 | 0 | 0 | 0 | CHO | 574.05949 | SB | - |
| 573.088599 | 22 | 26 | 15 | 0 | 0 | 0 | CHO | 574.095875 | SB | - |
| 573.109729 | 26 | 23 | 17 | 0 | 0 | 0 | CHO | 574.117005 | SB | - |
| 573.130558 | 30 | 26 | 5 | 4 | 3 | 0 | CHNOS | 574.137834 | SB | - |
| 573.167244 | 34 | 21 | 18 | 0 | 0 | 0 | CHO | 574.17452 | BB | - |
| 574.128311 | 29 | 21 | 10 | 5 | 2 | 0 | CHNOS | 575.135587 | SB | - |
| 574.164696 | 33 | 22 | 9 | 5 | 2 | 0 | CHNOS | 575.171972 | BB | - |
| 575.067864 | 20 | 25 | 16 | 0 | 0 | 0 | CHO | 576.07514 | SB | - |
| 575.104249 | 24 | 26 | 15 | 0 | 0 | 0 | CHO | 576.111525 | SB | 1 |
| 575.125379 | 28 | 23 | 17 | 0 | 0 | 0 | CHO | 576.132655 | SB | - |
| 576.143961 | 31 | 21 | 10 | 5 | 2 | 0 | CHNOS | 577.151237 | SB | - |
| 577.047129 | 18 | 24 | 17 | 0 | 0 | 0 | CHO | 578.054405 | SB | - |
| 577.083514 | 22 | 25 | 16 | 0 | 0 | 0 | CHO | 578.09079 | SB | - |
| 578.086841 | 25 | 19 | 12 | 5 | 2 | 0 | CHNOS | 579.094117 | SB | - |
| 578.123226 | 29 | 20 | 11 | 5 | 2 | 0 | CHNOS | 579.130502 | SB | - |
| 578.159611 | 33 | 21 | 10 | 5 | 2 | 0 | CHNOS | 579.166887 | SB | - |
| 579.062779 | 20 | 24 | 17 | 0 | 0 | 0 | CHO | 580.070055 | SB | - |
| 579.099164 | 24 | 25 | 16 | 0 | 0 | 0 | CHO | 580.10644 | SB | - |
| 579.193064 | 36 | 24 | 16 | 0 | 0 | 0 | CHO | 580.20034 | BB | - |
| 580.138876 | 31 | 20 | 11 | 5 | 2 | 0 | CHNOS | 581.146152 | SB | - |
| 581.078429 | 22 | 24 | 17 | 0 | 0 | 0 | CHO | 582.085705 | SB | - |
| 581.114814 | 26 | 25 | 16 | 0 | 0 | 0 | CHO | 582.12209 | SB | - |
| 581.172329 | 34 | 23 | 17 | 0 | 0 | 0 | CHO | 582.179605 | BB | - |
| 581.223969 | 38 | 28 | 13 | 0 | 0 | 0 | CHO | 582.231245 | BB | - |
| 582.118141 | 29 | 19 | 12 | 5 | 2 | 0 | CHNOS | 583.125417 | SB | - |
| 583.072949 | 20 | 27 | 15 | 0 | 0 | 0 | CHO | 584.080225 | SB | - |
| 583.094079 | 24 | 24 | 17 | 0 | 0 | 0 | CHO | 584.101355 | SB | - |
| 583.130464 | 28 | 25 | 16 | 0 | 0 | 0 | CHO | 584.13774 | SB | - |
| 584.170176 | 35 | 20 | 11 | 5 | 2 | 0 | CHNOS | 585.177452 | SB | - |
| 585.146114 | 30 | 25 | 16 | 0 | 0 | 0 | CHO | 586.15339 | SB | - |
| 585.166761 | 30 | 20 | 7 | 10 | 2 | 0 | CHNOS | 586.174037 | SB | - |
| 587.104249 | 24 | 27 | 15 | 0 | 0 | 0 | CHO | 588.111525 | SB | - |
| 587.125379 | 28 | 24 | 17 | 0 | 0 | 0 | CHO | 588.132655 | SB | - |
| 587.177019 | 32 | 29 | 13 | 0 | 0 | 0 | CHO | 588.184295 | BB | 3 |
| 588.107576 | 27 | 21 | 11 | 5 | 2 | 0 | CHNOS | 589.114852 | SB | - |
| 589.047129 | 18 | 25 | 17 | 0 | 0 | 0 | CHO | 590.054405 | SB | - |
| 589.083514 | 22 | 26 | 16 | 0 | 0 | 0 | CHO | 590.09079 | SB | - |
| 589.119899 | 26 | 27 | 15 | 0 | 0 | 0 | CHO | 590.127175 | SB | 1 |
| 589.213799 | 38 | 26 | 15 | 0 | 0 | 0 | CHO | 590.221075 | SB | 2 |
| 591.062779 | 20 | 25 | 17 | 0 | 0 | 0 | CHO | 592.070055 | SB | - |
| 591.099164 | 24 | 26 | 16 | 0 | 0 | 0 | CHO | 592.10644 | SB | - |
| 591.229449 | 40 | 26 | 15 | 0 | 0 | 0 | CHO | 592.236725 | SB | - |
| 591.317474 | 48 | 32 | 10 | 0 | 0 | 0 | CHO | 592.32475 | BB | 1 |
| 593.042044 | 18 | 24 | 18 | 0 | 0 | 0 | CHO | 594.04932 | SB | - |
| 593.078429 | 22 | 25 | 17 | 0 | 0 | 0 | CHO | 594.085705 | SB | - |
| 593.114814 | 26 | 26 | 16 | 0 | 0 | 0 | CHO | 594.12209 | SB | - |
| 594.081756 | 25 | 19 | 13 | 5 | 2 | 0 | CHNOS | 595.089032 | SB | - |
| 594.118141 | 29 | 20 | 12 | 5 | 2 | 0 | CHNOS | 595.125417 | SB | - |
| 594.154526 | 33 | 21 | 11 | 5 | 2 | 0 | CHNOS | 595.161802 | SB | - |
| 595.057694 | 20 | 24 | 18 | 0 | 0 | 0 | CHO | 596.06497 | SB | - |
| 595.072949 | 20 | 28 | 15 | 0 | 0 | 0 | CHO | 596.080225 | SB | - |
| 595.109334 | 24 | 29 | 14 | 0 | 0 | 0 | CHO | 596.11661 | SB | - |
| 595.130464 | 28 | 26 | 16 | 0 | 0 | 0 | CHO | 596.13774 | SB | 24 |
| 595.187979 | 36 | 24 | 17 | 0 | 0 | 0 | CHO | 596.195255 | BB | - |
| 596.097406 | 27 | 19 | 13 | 5 | 2 | 0 | CHNOS | 597.104682 | SB | - |
| 596.133791 | 31 | 20 | 12 | 5 | 2 | 0 | CHNOS | 597.141067 | SB | 24 |
| 597.073344 | 22 | 24 | 18 | 0 | 0 | 0 | CHO | 598.08062 | SB | - |
| 597.109729 | 26 | 25 | 17 | 0 | 0 | 0 | CHO | 598.117005 | SB | - |
| 597.146114 | 30 | 26 | 16 | 0 | 0 | 0 | CHO | 598.15339 | SB | - |
| 598.113056 | 29 | 19 | 13 | 5 | 2 | 0 | CHNOS | 599.120332 | SB | - |
| 598.185826 | 37 | 21 | 11 | 5 | 2 | 0 | CHNOS | 599.193102 | SB | - |
| 599.088994 | 24 | 24 | 18 | 0 | 0 | 0 | CHO | 600.09627 | SB | - |
| 599.104249 | 24 | 28 | 15 | 0 | 0 | 0 | CHO | 600.111525 | SB | 7 |
| 599.125379 | 28 | 25 | 17 | 0 | 0 | 0 | CHO | 600.132655 | SB | - |
| 599.126064 | 28 | 18 | 15 | 6 | 1 | 0 | CHNOS | 600.13334 | BB | - |
| 601.047129 | 18 | 26 | 17 | 0 | 0 | 0 | CHO | 602.054405 | SB | - |
| 601.083514 | 22 | 27 | 16 | 0 | 0 | 0 | CHO | 602.09079 | SB | - |
| 601.104644 | 26 | 24 | 18 | 0 | 0 | 0 | CHO | 602.11192 | SB | - |
| 601.119899 | 26 | 28 | 15 | 0 | 0 | 0 | CHO | 602.127175 | SB | 1 |
| 602.086841 | 25 | 21 | 12 | 5 | 2 | 0 | CHNOS | 603.094117 | SB | - |
| 602.123226 | 29 | 22 | 11 | 5 | 2 | 0 | CHNOS | 603.130502 | SB | 1 |
| 603.120294 | 28 | 24 | 18 | 0 | 0 | 0 | CHO | 604.12757 | SB | - |
| 605.078429 | 22 | 26 | 17 | 0 | 0 | 0 | CHO | 606.085705 | SB | - |
| 605.114814 | 26 | 27 | 16 | 0 | 0 | 0 | CHO | 606.12209 | SB | - |
| 605.135944 | 30 | 24 | 18 | 0 | 0 | 0 | CHO | 606.14322 | SB | - |
| 605.171846 | 30 | 23 | 6 | 10 | 2 | 0 | CHNOS | 606.179122 | SB | - |
| 606.081756 | 25 | 20 | 13 | 5 | 2 | 0 | CHNOS | 607.089032 | SB | - |
| 606.118141 | 29 | 21 | 12 | 5 | 2 | 0 | CHNOS | 607.125417 | SB | - |
| 607.094079 | 24 | 26 | 17 | 0 | 0 | 0 | CHO | 608.101355 | SB | - |
| 607.130464 | 28 | 27 | 16 | 0 | 0 | 0 | CHO | 608.13774 | SB | 6 |
| 608.133791 | 31 | 21 | 12 | 5 | 2 | 0 | CHNOS | 609.141067 | SB | 6 |
| 609.073344 | 22 | 25 | 18 | 0 | 0 | 0 | CHO | 610.08062 | SB | - |
| 609.109729 | 26 | 26 | 17 | 0 | 0 | 0 | CHO | 610.117005 | SB | - |
| 609.146114 | 30 | 27 | 16 | 0 | 0 | 0 | CHO | 610.15339 | SB | 88 |
| 611.067864 | 20 | 28 | 16 | 0 | 0 | 0 | CHO | 612.07514 | SB | - |
| 611.088994 | 24 | 25 | 18 | 0 | 0 | 0 | CHO | 612.09627 | SB | - |
| 611.125379 | 28 | 26 | 17 | 0 | 0 | 0 | CHO | 612.132655 | SB | 1 |
| 611.161764 | 32 | 27 | 16 | 0 | 0 | 0 | CHO | 612.16904 | SB | 4 |
| 611.177019 | 32 | 31 | 13 | 0 | 0 | 0 | CHO | 612.184295 | SB | - |
| 611.182894 | 36 | 24 | 18 | 0 | 0 | 0 | CHO | 612.19017 | BB | - |
| 611.198149 | 36 | 28 | 15 | 0 | 0 | 0 | CHO | 612.205425 | BB | - |
| 613.084199 | 22 | 21 | 14 | 6 | 1 | 0 | CHNOS | 614.091475 | SB | - |
| 613.119899 | 26 | 29 | 15 | 0 | 0 | 0 | CHO | 614.127175 | SB | - |
| 613.141029 | 30 | 26 | 17 | 0 | 0 | 0 | CHO | 614.148305 | SB | - |
| 613.156284 | 30 | 30 | 14 | 0 | 0 | 0 | CHO | 614.16356 | SB | 1 |
| 615.099164 | 24 | 28 | 16 | 0 | 0 | 0 | CHO | 616.10644 | SB | 9 |
| 615.135549 | 28 | 29 | 15 | 0 | 0 | 0 | CHO | 616.142825 | SB | - |
| 615.28446 | 48 | 30 | 11 | 0 | 1 | 0 | CHOS | 616.291736 | SB | - |
| 616.287571 | 43 | 31 | 10 | 3 | 0 | 0 | CHNO | 617.294847 | SB | - |
| 617.042044 | 18 | 26 | 18 | 0 | 0 | 0 | CHO | 618.04932 | SB | - |
| 617.114814 | 26 | 28 | 16 | 0 | 0 | 0 | CHO | 618.12209 | SB | - |
| 618.176307 | 33 | 28 | 11 | 3 | 1 | 0 | CHNOS | 619.183583 | SB | - |
| 619.130464 | 28 | 28 | 16 | 0 | 0 | 0 | CHO | 620.13774 | SB | - |
| 621.036959 | 18 | 25 | 19 | 0 | 0 | 0 | CHO | 622.044235 | SB | - |
| 621.073344 | 22 | 26 | 18 | 0 | 0 | 0 | CHO | 622.08062 | SB | - |
| 621.109729 | 26 | 27 | 17 | 0 | 0 | 0 | CHO | 622.117005 | SB | 2 |
| 621.219569 | 38 | 23 | 12 | 6 | 1 | 0 | CHNOS | 622.226845 | SB | - |
| 622.113056 | 29 | 21 | 13 | 5 | 2 | 0 | CHNOS | 623.120332 | SB | 2 |
| 622.185826 | 37 | 23 | 11 | 5 | 2 | 0 | CHNOS | 623.193102 | SB | - |
| 623.088994 | 24 | 26 | 18 | 0 | 0 | 0 | CHO | 624.09627 | SB | - |
| 623.125379 | 28 | 27 | 17 | 0 | 0 | 0 | CHO | 624.132655 | SB | 6 |
| 625.084199 | 22 | 22 | 14 | 6 | 1 | 0 | CHNOS | 626.091475 | SB | - |
| 625.119899 | 26 | 30 | 15 | 0 | 0 | 0 | CHO | 626.127175 | SB | 7 |
| 625.141029 | 30 | 27 | 17 | 0 | 0 | 0 | CHO | 626.148305 | SB | 37 |
| 625.213799 | 38 | 29 | 15 | 0 | 0 | 0 | CHO | 626.221075 | BB | 2 |
| 627.136234 | 28 | 23 | 13 | 6 | 1 | 0 | CHNOS | 628.14351 | SB | - |
| 629.114814 | 26 | 29 | 16 | 0 | 0 | 0 | CHO | 630.12209 | SB | 3 |
| 629.187584 | 34 | 31 | 14 | 0 | 0 | 0 | CHO | 630.19486 | BB | - |
| 629.188269 | 34 | 24 | 12 | 6 | 1 | 0 | CHNOS | 630.195545 | SB | - |
| 631.094079 | 24 | 28 | 17 | 0 | 0 | 0 | CHO | 632.101355 | SB | 5 |
| 631.130464 | 28 | 29 | 16 | 0 | 0 | 0 | CHO | 632.13774 | SB | - |
| 631.151594 | 32 | 26 | 18 | 0 | 0 | 0 | CHO | 632.15887 | SB | - |
| 631.279375 | 48 | 30 | 12 | 0 | 1 | 0 | CHOS | 632.286651 | SB | - |
| 633.073344 | 22 | 27 | 18 | 0 | 0 | 0 | CHO | 634.08062 | SB | 2 |
| 633.167244 | 34 | 26 | 18 | 0 | 0 | 0 | CHO | 634.17452 | SB | - |
| 635.052609 | 20 | 26 | 19 | 0 | 0 | 0 | CHO | 636.059885 | SB | - |
| 635.126064 | 28 | 21 | 15 | 6 | 1 | 0 | CHNOS | 636.13334 | BB | - |
| 635.343689 | 52 | 34 | 11 | 0 | 0 | 0 | CHO | 636.350965 | SB | - |
| 636.128706 | 31 | 22 | 13 | 5 | 2 | 0 | CHNOS | 637.135982 | SB | - |
| 637.068259 | 22 | 26 | 19 | 0 | 0 | 0 | CHO | 638.075535 | SB | - |
| 637.104644 | 26 | 27 | 18 | 0 | 0 | 0 | CHO | 638.11192 | SB | 9 |
| 637.141029 | 30 | 28 | 17 | 0 | 0 | 0 | CHO | 638.148305 | SB | 3 |
| 637.198544 | 38 | 26 | 18 | 0 | 0 | 0 | CHO | 638.20582 | BB | - |
| 639.119643 | 32 | 19 | 20 | 2 | 1 | 0 | CHNOS | 640.126919 | SB | - |
| 639.156679 | 32 | 28 | 17 | 0 | 0 | 0 | CHO | 640.163955 | SB | 39 |
| 639.193749 | 36 | 22 | 14 | 6 | 1 | 0 | CHNOS | 640.201025 | SB | - |
| 640.123621 | 31 | 21 | 14 | 5 | 2 | 0 | CHNOS | 641.130897 | SB | - |
| 641.078429 | 22 | 29 | 17 | 0 | 0 | 0 | CHO | 642.085705 | SB | - |
| 641.099559 | 26 | 26 | 19 | 0 | 0 | 0 | CHO | 642.106835 | SB | - |
| 643.130464 | 28 | 30 | 16 | 0 | 0 | 0 | CHO | 644.13774 | SB | - |
| 643.151594 | 32 | 27 | 18 | 0 | 0 | 0 | CHO | 644.15887 | SB | - |
| 645.073344 | 22 | 28 | 18 | 0 | 0 | 0 | CHO | 646.08062 | SB | - |
| 645.109729 | 26 | 29 | 17 | 0 | 0 | 0 | CHO | 646.117005 | SB | - |
| 645.146114 | 30 | 30 | 16 | 0 | 0 | 0 | CHO | 646.15339 | SB | - |
| 645.146799 | 30 | 23 | 14 | 6 | 1 | 0 | CHNOS | 646.154075 | BB | - |
| 647.088994 | 24 | 28 | 18 | 0 | 0 | 0 | CHO | 648.09627 | SB | - |
| 647.125379 | 28 | 29 | 17 | 0 | 0 | 0 | CHO | 648.132655 | SB | - |
| 647.161764 | 32 | 30 | 16 | 0 | 0 | 0 | CHO | 648.16904 | SB | - |
| 647.198149 | 36 | 31 | 15 | 0 | 0 | 0 | CHO | 648.205425 | SB | 1 |
| 649.068259 | 22 | 27 | 19 | 0 | 0 | 0 | CHO | 650.075535 | SB | - |
| 649.213799 | 38 | 31 | 15 | 0 | 0 | 0 | CHO | 650.221075 | SB | 1 |
| 649.359339 | 54 | 35 | 11 | 0 | 0 | 0 | CHO | 650.366615 | SB | - |
| 651.083909 | 24 | 27 | 19 | 0 | 0 | 0 | CHO | 652.091185 | SB | - |
| 651.120294 | 28 | 28 | 18 | 0 | 0 | 0 | CHO | 652.12757 | SB | 4 |
| 651.193064 | 36 | 30 | 16 | 0 | 0 | 0 | CHO | 652.20034 | BB | 7 |
| 653.135944 | 30 | 28 | 18 | 0 | 0 | 0 | CHO | 654.14322 | SB | 1 |
| 653.208231 | 34 | 28 | 5 | 10 | 2 | 0 | CHNOS | 654.215507 | SB | - |
| 653.208714 | 38 | 30 | 16 | 0 | 0 | 0 | CHO | 654.21599 | BB | - |
| 655.130464 | 28 | 31 | 16 | 0 | 0 | 0 | CHO | 656.13774 | SB | 3 |
| 655.187979 | 36 | 29 | 17 | 0 | 0 | 0 | CHO | 656.195255 | SB | 1 |
| 655.188664 | 36 | 22 | 15 | 6 | 1 | 0 | CHNOS | 656.19594 | BB | - |
| 657.073344 | 22 | 29 | 18 | 0 | 0 | 0 | CHO | 658.08062 | SB | - |
| 657.109729 | 26 | 30 | 17 | 0 | 0 | 0 | CHO | 658.117005 | SB | 1 |
| 657.130859 | 30 | 27 | 19 | 0 | 0 | 0 | CHO | 658.138135 | SB | - |
| 657.146114 | 30 | 31 | 16 | 0 | 0 | 0 | CHO | 658.15339 | SB | - |
| 659.125379 | 28 | 30 | 17 | 0 | 0 | 0 | CHO | 660.132655 | SB | - |
| 659.161764 | 32 | 31 | 16 | 0 | 0 | 0 | CHO | 660.16904 | SB | - |
| 659.198834 | 36 | 25 | 13 | 6 | 1 | 0 | CHNOS | 660.20611 | BB | - |
| 661.104644 | 26 | 29 | 18 | 0 | 0 | 0 | CHO | 662.11192 | SB | - |
| 661.141029 | 30 | 30 | 17 | 0 | 0 | 0 | CHO | 662.148305 | SB | - |
| 661.177414 | 34 | 31 | 16 | 0 | 0 | 0 | CHO | 662.18469 | SB | 2 |
| 661.178099 | 34 | 24 | 14 | 6 | 1 | 0 | CHNOS | 662.185375 | BB | - |
| 661.183457 | 30 | 30 | 8 | 8 | 1 | 0 | CHNOS | 662.190733 | SB | - |
| 663.083909 | 24 | 28 | 19 | 0 | 0 | 0 | CHO | 664.091185 | SB | - |
| 663.193064 | 36 | 31 | 16 | 0 | 0 | 0 | CHO | 664.20034 | BB | 3 |
| 665.135944 | 30 | 29 | 18 | 0 | 0 | 0 | CHO | 666.14322 | SB | - |
| 667.094079 | 24 | 31 | 17 | 0 | 0 | 0 | CHO | 668.101355 | SB | - |
| 667.115209 | 28 | 28 | 19 | 0 | 0 | 0 | CHO | 668.122485 | SB | - |
| 667.151594 | 32 | 29 | 18 | 0 | 0 | 0 | CHO | 668.15887 | SB | 3 |
| 667.369904 | 56 | 35 | 12 | 0 | 0 | 0 | CHO | 668.37718 | SB | - |
| 668.154921 | 35 | 23 | 14 | 5 | 2 | 0 | CHNOS | 669.162197 | SB | 3 |
| 669.204314 | 38 | 23 | 15 | 6 | 1 | 0 | CHNOS | 670.21159 | SB | - |
| 670.134186 | 33 | 22 | 15 | 5 | 2 | 0 | CHNOS | 671.141462 | SB | - |
| 671.088343 | 28 | 22 | 20 | 2 | 1 | 0 | CHNOS | 672.095619 | SB | - |
| 671.125379 | 28 | 31 | 17 | 0 | 0 | 0 | CHO | 672.132655 | SB | - |
| 671.126064 | 28 | 24 | 15 | 6 | 1 | 0 | CHNOS | 672.13334 | BB | - |
| 671.146509 | 32 | 28 | 19 | 0 | 0 | 0 | CHO | 672.153785 | SB | - |
| 671.161764 | 32 | 32 | 16 | 0 | 0 | 0 | CHO | 672.16904 | SB | - |
| 671.182894 | 36 | 29 | 18 | 0 | 0 | 0 | CHO | 672.19017 | SB | - |
| 671.183579 | 36 | 22 | 16 | 6 | 1 | 0 | CHNOS | 672.190855 | BB | - |
| 673.141029 | 30 | 31 | 17 | 0 | 0 | 0 | CHO | 674.148305 | SB | - |
| 673.162159 | 34 | 28 | 19 | 0 | 0 | 0 | CHO | 674.169435 | SB | - |
| 673.177414 | 34 | 32 | 16 | 0 | 0 | 0 | CHO | 674.18469 | SB | - |
| 675.083909 | 24 | 29 | 19 | 0 | 0 | 0 | CHO | 676.091185 | SB | - |
| 675.156679 | 32 | 31 | 17 | 0 | 0 | 0 | CHO | 676.163955 | SB | - |
| 677.099559 | 26 | 29 | 19 | 0 | 0 | 0 | CHO | 678.106835 | SB | - |
| 677.135944 | 30 | 30 | 18 | 0 | 0 | 0 | CHO | 678.14322 | SB | - |
| 677.354254 | 54 | 36 | 12 | 0 | 0 | 0 | CHO | 678.36153 | SB | - |
| 678.102886 | 29 | 23 | 15 | 5 | 2 | 0 | CHNOS | 679.110162 | SB | - |
| 678.139271 | 33 | 24 | 14 | 5 | 2 | 0 | CHNOS | 679.146547 | SB | - |
| 679.115209 | 28 | 29 | 19 | 0 | 0 | 0 | CHO | 680.122485 | SB | - |
| 679.333519 | 52 | 35 | 13 | 0 | 0 | 0 | CHO | 680.340795 | SB | - |
| 681.094474 | 26 | 28 | 20 | 0 | 0 | 0 | CHO | 682.10175 | SB | - |
| 681.130208 | 34 | 21 | 21 | 2 | 1 | 0 | CHNOS | 682.137484 | SB | - |
| 681.181848 | 38 | 26 | 17 | 2 | 1 | 0 | CHNOS | 682.189124 | BB | - |
| 681.385554 | 58 | 36 | 12 | 0 | 0 | 0 | CHO | 682.39283 | SB | 1 |
| 683.088994 | 24 | 31 | 18 | 0 | 0 | 0 | CHO | 684.09627 | SB | - |
| 683.100912 | 24 | 23 | 15 | 8 | 1 | 0 | CHNOS | 684.108188 | BB | - |
| 683.146509 | 32 | 29 | 19 | 0 | 0 | 0 | CHO | 684.153785 | SB | - |
| 683.162449 | 32 | 26 | 14 | 6 | 1 | 0 | CHNOS | 684.169725 | SB | - |
| 685.104644 | 26 | 31 | 18 | 0 | 0 | 0 | CHO | 686.11192 | SB | - |
| 685.140378 | 34 | 24 | 19 | 2 | 1 | 0 | CHNOS | 686.147654 | SB | - |
| 685.162159 | 34 | 29 | 19 | 0 | 0 | 0 | CHO | 686.169435 | SB | - |
| 687.083909 | 24 | 30 | 19 | 0 | 0 | 0 | CHO | 688.091185 | SB | - |
| 687.140941 | 28 | 26 | 9 | 10 | 2 | 0 | CHNOS | 688.148217 | SB | - |
| 689.135944 | 30 | 31 | 18 | 0 | 0 | 0 | CHO | 690.14322 | SB | - |
| 689.172329 | 34 | 32 | 17 | 0 | 0 | 0 | CHO | 690.179605 | SB | - |
| 691.151594 | 32 | 31 | 18 | 0 | 0 | 0 | CHO | 692.15887 | SB | - |
| 693.130859 | 30 | 30 | 19 | 0 | 0 | 0 | CHO | 694.138135 | SB | 1 |
| 693.349169 | 54 | 36 | 13 | 0 | 0 | 0 | CHO | 694.356445 | SB | - |
| 695.110124 | 28 | 29 | 20 | 0 | 0 | 0 | CHO | 696.1174 | SB | - |
| 695.146509 | 32 | 30 | 19 | 0 | 0 | 0 | CHO | 696.153785 | SB | 3 |
| 695.364819 | 56 | 36 | 13 | 0 | 0 | 0 | CHO | 696.372095 | SB | - |
| 696.368146 | 59 | 30 | 9 | 5 | 2 | 0 | CHNOS | 697.375422 | SB | - |
| 697.380469 | 58 | 36 | 13 | 0 | 0 | 0 | CHO | 698.387745 | SB | - |
| 698.383796 | 61 | 30 | 9 | 5 | 2 | 0 | CHNOS | 699.391072 | SB | - |
| 699.120294 | 28 | 32 | 18 | 0 | 0 | 0 | CHO | 700.12757 | SB | - |
| 699.193064 | 36 | 34 | 16 | 0 | 0 | 0 | CHO | 700.20034 | BB | - |
| 699.387089 | 60 | 39 | 5 | 2 | 2 | 0 | CHNOS | 700.394365 | SB | - |
| 699.396119 | 60 | 36 | 13 | 0 | 0 | 0 | CHO | 700.403395 | SB | - |
| 701.098908 | 30 | 23 | 21 | 2 | 1 | 0 | CHNOS | 702.106184 | SB | - |
| 701.135293 | 34 | 24 | 20 | 2 | 1 | 0 | CHNOS | 702.142569 | SB | - |
| 701.193459 | 38 | 30 | 19 | 0 | 0 | 0 | CHO | 702.200735 | SB | - |
| 703.115209 | 28 | 31 | 19 | 0 | 0 | 0 | CHO | 704.122485 | SB | - |
| 703.187979 | 36 | 33 | 17 | 0 | 0 | 0 | CHO | 704.195255 | BB | - |
| 705.094474 | 26 | 30 | 20 | 0 | 0 | 0 | CHO | 706.10175 | SB | - |
| 705.130859 | 30 | 31 | 19 | 0 | 0 | 0 | CHO | 706.138135 | SB | - |
| 705.167244 | 34 | 32 | 18 | 0 | 0 | 0 | CHO | 706.17452 | SB | 2 |
| 707.110124 | 28 | 30 | 20 | 0 | 0 | 0 | CHO | 708.1174 | SB | - |
| 707.219964 | 40 | 26 | 15 | 6 | 1 | 0 | CHNOS | 708.22724 | BB | - |
| 709.125774 | 30 | 30 | 20 | 0 | 0 | 0 | CHO | 710.13305 | SB | - |
| 709.162159 | 34 | 31 | 19 | 0 | 0 | 0 | CHO | 710.169435 | SB | 5 |
| 711.119643 | 32 | 25 | 20 | 2 | 1 | 0 | CHNOS | 712.126919 | SB | - |
| 711.177809 | 36 | 31 | 19 | 0 | 0 | 0 | CHO | 712.185085 | SB | 3 |
| 713.375384 | 58 | 36 | 14 | 0 | 0 | 0 | CHO | 714.38266 | SB | - |
| 714.378711 | 61 | 30 | 10 | 5 | 2 | 0 | CHNOS | 715.385987 | SB | - |
| 715.151594 | 32 | 33 | 18 | 0 | 0 | 0 | CHO | 716.15887 | SB | - |
| 715.187979 | 36 | 34 | 17 | 0 | 0 | 0 | CHO | 716.195255 | BB | - |
| 717.094474 | 26 | 31 | 20 | 0 | 0 | 0 | CHO | 718.10175 | SB | - |
| 717.167244 | 34 | 33 | 18 | 0 | 0 | 0 | CHO | 718.17452 | SB | - |
| 719.110124 | 28 | 31 | 20 | 0 | 0 | 0 | CHO | 720.1174 | SB | - |
| 721.162844 | 34 | 25 | 17 | 6 | 1 | 0 | CHNOS | 722.17012 | SB | 4 |
| 723.178494 | 36 | 25 | 17 | 6 | 1 | 0 | CHNOS | 724.18577 | SB | 2 |
| 725.157759 | 34 | 24 | 18 | 6 | 1 | 0 | CHNOS | 726.165035 | SB | - |
| 725.173014 | 34 | 28 | 15 | 6 | 1 | 0 | CHNOS | 726.18029 | SB | - |
| 725.193459 | 38 | 32 | 19 | 0 | 0 | 0 | CHO | 726.200735 | SB | 17 |
| 725.375384 | 58 | 37 | 14 | 0 | 0 | 0 | CHO | 726.38266 | SB | - |
| 727.151594 | 32 | 34 | 18 | 0 | 0 | 0 | CHO | 728.15887 | SB | - |
| 729.204314 | 38 | 28 | 15 | 6 | 1 | 0 | CHNOS | 730.21159 | SB | - |
| 729.225444 | 42 | 25 | 17 | 6 | 1 | 0 | CHNOS | 730.23272 | BB | - |
| 729.406684 | 62 | 37 | 14 | 0 | 0 | 0 | CHO | 730.41396 | SB | - |
| 731.146509 | 32 | 33 | 19 | 0 | 0 | 0 | CHO | 732.153785 | SB | - |
| 733.125774 | 30 | 32 | 20 | 0 | 0 | 0 | CHO | 734.13305 | SB | - |
| 737.121374 | 30 | 24 | 19 | 6 | 1 | 0 | CHNOS | 738.12865 | SB | - |
| 739.173409 | 36 | 25 | 18 | 6 | 1 | 0 | CHNOS | 740.180685 | SB | - |
| 739.391034 | 60 | 38 | 14 | 0 | 0 | 0 | CHO | 740.39831 | SB | - |
| 745.126459 | 30 | 26 | 18 | 6 | 1 | 0 | CHNOS | 746.133735 | SB | - |
| 747.105039 | 28 | 32 | 21 | 0 | 0 | 0 | CHO | 748.112315 | SB | - |
| 749.157759 | 34 | 26 | 18 | 6 | 1 | 0 | CHNOS | 750.165035 | SB | - |
| 751.209794 | 40 | 27 | 17 | 6 | 1 | 0 | CHNOS | 752.21707 | BB | 2 |
| 761.157074 | 34 | 34 | 20 | 0 | 0 | 0 | CHO | 762.16435 | SB | 1 |
| 765.151989 | 34 | 33 | 21 | 0 | 0 | 0 | CHO | 766.159265 | SB | - |
| 801.152674 | 34 | 29 | 19 | 6 | 1 | 0 | CHNOS | 802.15995 | SB | - |
| 805.183974 | 38 | 29 | 19 | 6 | 1 | 0 | CHNOS | 806.19125 | SB | - |
| 819.324034 | 52 | 37 | 13 | 6 | 1 | 0 | CHNOS | 820.33131 | SB | - |
| 875.428209 | 68 | 42 | 19 | 0 | 0 | 0 | CHO | 876.435485 | BB | - |
